# DMARS_WGO: a deep reinforcement-driven hybrid metaheuristic for intelligent adaptive optimization

**DOI:** 10.1038/s41598-026-46134-4

**Published:** 2026-04-22

**Authors:** Nada R. Yousif, Eman M. El-Gendy, Amira Y. Haikal

**Affiliations:** 1 Computers and Control Systems Engineering Department, Faculty of Engineering, Mansoura University, Mansoura, Egypt; 2 Electronics and Communications Engineering Department, Mansoura Higher Institute for Engineering and Technology, Mansoura, Egypt

**Keywords:** Metaheuristic optimization, Walrus–Gazelle optimizer, Reinforcement learning, Dual-mode Q-learning, Deep Q-network, Exploration–exploitation balance, DMARS_WGO algorithm, Engineering, Mathematics and computing

## Abstract

Metaheuristics optimization algorithms have established themselves as a new cornerstone in the area of complex, nonlinear, and high-dimensional problems of science and engineering. Most existing algorithms, however, still do not manage to balance exploration and exploitation and still experience stagnation and premature convergence. Though there have been many developments, most of the available algorithms are not yet optimized in terms of premature convergence and adaptive decision-making ability. This paper presents two smart reinforcement-based schemes, one Adaptive Intelligent Reinforced Walrus-Gazelle Optimizer (AIRE_WGO), and another Dual-Mode Adaptive Reinforced Switching Walrus-Gazelle Optimizer (DMARS_WGO). The suggested AIRE_WGO steps up the classical Walrus gazelle hybrid, where it implements Q-learning-based control of the agent behavior, adjusts its parameters in response to the current environment, and considers diversity-informed mutations in order to dynamically switch between global exploration and local exploitation. However, DMARS_WGO incorporates a dual-agent reinforcement framework that combines tabular Q-learning and deep Q-network (DQN) reasoning. When interacting with diverse populations, DMARS_WGO autonomously switches between its learning dominance as a result of real-time feedback about the population structure, the rate of improvement, and the level of stagnation. Moreover, the cross-agent knowledge-sharing process provides a two-way experience transfer between Q-learning and DQN modules, which strengthens cooperative intelligence and stability. Extensive tests of CEC2017 and CEC2022 benchmark suites and six engineering design problems prove that the proposed algorithms, especially DMARS_WGO, are more successful compared to nine recent state-of-the-art optimizers including Golden Jackal Optimization (GJO), Osprey Optimization Algorithm (OOA), Pelican Optimization Algorithm (POA), Rat Swarm Optimizer (RSO), Smell Agent Optimization (SAO), Channa argus Optimizer (CAO), Rüppell’s Fox Optimizer (RFO), Mantis Shrimp Optimization Algorithm (MShOA), and Adaptive L-SHADE (ALSHADE). On CEC2017, DMARS_WGO achieved first rank in 26 out of 29 benchmark functions and obtained the best overall Friedman mean rank. On CEC2022, it ranked first in 8 out of 12 functions and achieved the best overall ranking among all compared algorithms. Similarly, across the six engineering design problems, DMARS_WGO secured first position in 4 out of 6 cases and achieved the best overall ranking performance. Wilcoxon Signed-Rank and Friedman mean-rank statistical tests prove that DMARS_WGO is significantly superior. Its ability to smartly self-adapt its search dynamics is pointed out in the results, and makes it a strong and robust optimizer of real-world engineering problems.

## Introduction

**Fig. 1:**
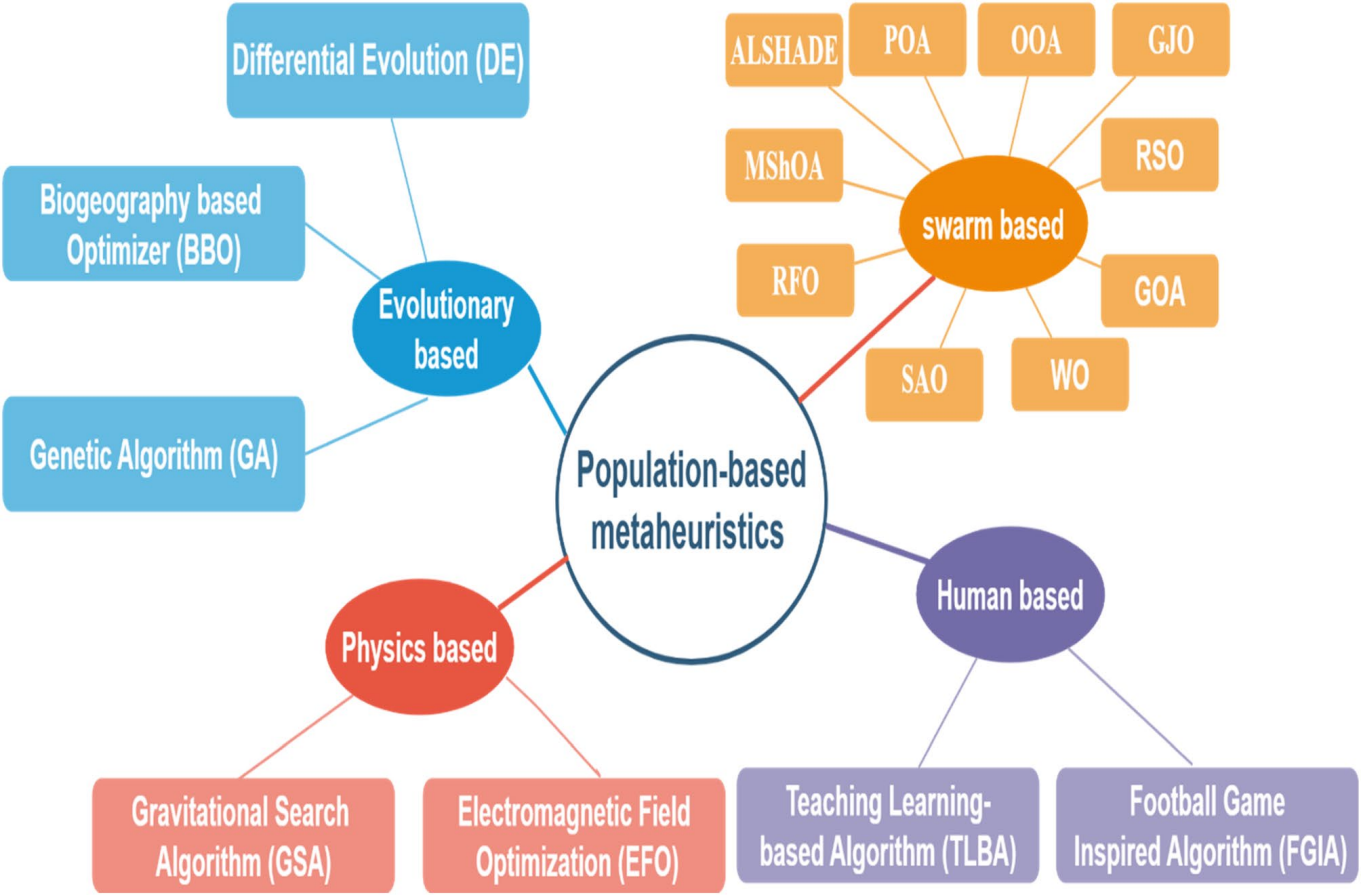
Standardization of metaheuristic methods.

Science and engineering optimization problems can be multimodal, nonlinear, and high-dimensional, making them hard to find solutions to with classical optimization algorithms^1^. Based on derivative information and convex landscapes, these classical optimization algorithms, which include gradient descent^2^, linear programming^3^, and Newton-based methods^4^, are only applicable on small scale or smooth problems, and instead become easily stuck and lose global optimality when presented with irregular search space, discontinuities, and multiple local optima^5^. In order to overcome these shortfalls, heuristic and metaheuristic algorithms are introduced to introduce an interesting and useful optimization system^6^. The heuristic algorithms are applied to provide a rough solution to the complex optimization problems on the basis of the experience-based approaches, which typically rely on the concepts of trial-and-error and local search^7^. They aim to obtain satisfactory, not necessarily optimal solutions in a reasonable time, and may thus be handy when certain analytical processes are not practicable^8^. Metaheuristic algorithms take these concepts to a more abstract level and bring in adaptive processes that balance between exploration and exploitation in the search space^9,10^. The popularity of metaheuristics compared to precise techniques of finding solutions to optimization problems is due to the simplicity and strength of the solutions that they offer in various disciplines such as engineering, commerce, transportation, and the social sciences^11^. The idea of a metaheuristic is to solve diverse and difficult optimization problems without significant problem mapping^12^. The suffix meta connotes more than heuristics and the fact that they are capable of generalizing the strategies of tackling problems as opposed to making use of the domain-specific rules^13,14^. There are numerous subclasses of metaheuristic algorithms based on the theory they are based on, as illustrated in Fig. 1. The theory of evolution by Darwin led to the development of Evolutionary Algorithms (EA)^15^. They are based on the natural evolution process. They are based on the theory of survival of the fittest, reproduction, and mutation. Genetic Algorithm (GA)^16^, Differential Evolution (DE)^17^, and Biogeography-based Optimizer^18^. Physics-based algorithms (PA) are based on physical laws and principles^19^. Some such algorithms include the Gravitational Search Algorithm (GSA)^20^ and the Electromagnetic Field Optimization (EFO)^21^. Human-based algorithms (HA) are based on human knowledge, intelligence, and experience^22^. Examples of these algorithms are the Teaching Learning based Algorithm (TLBA)^23^ and Football Game Inspired Algorithm (FGIA)^24^. Swarm-based algorithms (SA) are based on the behavior of social animals like birds, bees, and ants^25^. Examples of these algorithms are Golden Jackal Optimization (GJO)^26^, Osprey Optimization Algorithm (OOA)^27^, Pelican Optimization Algorithm (POA)^28^, Mantis Shrimp Optimization Algorithm (MShOA)^29^, ALSHADE^30^, and sparrow search algorithm (SSA)^31^. Also there are other classes of metaheuristic algorithms based on human behavior, animals, plants, ecological environments, and global natural inspiration^32–34^. Trajectory-based algorithms, such as Simulated Annealing (SA)^35^, iteratively refine a single candidate solution as it moves through the search space. However, since they focus on one trajectory at a time, these algorithms are likely to get stuck in local optima, which curtails their capability of achieving the global optimum^36^. In contrast, population-based algorithms develop several candidate solutions at the same time, including Particle Swarm Optimization (PSO)^37^, Grey Wolf Optimizer (GWO)^38^, and Whale Optimization Algorithm (WOA)^39,40^. This collective search behavior improves the exploration and ensures that the algorithm is more effective in escaping the local traps. Numerous adaptive population-based algorithms are inspired by natural processes, including selection, mutation, and recombination processes, in which the fittest individuals are selectively retained to steer the population to global optimality^41,42^.

Recent research has focused on enhancing metaheuristic performance through hybridization and adaptive learning mechanisms such as improved variants of Particle Swarm Optimization (PSO) incorporate quantum-inspired operators and adaptive mutation strategies^43,44^, improved variants of Salp Swarm Algorithm (SSA)^45–47^, and improved variant of sparrow search algorithm (SSA) for solving the multiobjective optimization problems^48^.

Among swarm-based metaheuristic optimization algorithms, the Walrus Optimizer (WO)^49^ and the Gazelle Optimization Algorithm (GOA)^50^ are two of the most recent swarm-based metaheuristic optimization algorithms whose optimization behaviors are complementary to each other. WO has a strong local exploitation, whereas GOA has a high global exploration. Although each of them has its own advantages, all the algorithms have difficulties in preserving a constant balance between exploration and exploitation at various levels of optimization. Thus, strategies to combine the benefits of WO and GOA have been created, which can be considered a more balanced and robust performance in addressing complex optimization problems.

However, recent research has tried to improve swarm-based optimizers with diversity-conserving and adaptive learning schemes. As an example, Hussien and Amin^51^ developed a better implementation of the Harris Hawks Optimization algorithm (IHHO) that incorporates Opposition-Based Learning (OBL), Chaotic Local Search (CLS) and a self-adaptive control strategy to enhance convergence rate and resilience. The suggested IHHO framework increases diversity of the population during the period of its initializing and updating and reinforces the local exploitation of the promising areas. Large-scale tests of the IEEE CEC 2017 benchmark collection and practical engineering issues showed large advances on both the classical HHO and various state-of-the-art algorithms. Nevertheless, with all these developments, these hybrid enhancement schemes are mainly rule-based and lack cooperative multi-agent reinforcement to actively control structural search behaviors in various optimization landscapes. This drawback inspires the creation of smarter reinforcement-based hybrid models, as the one suggested in this study. The other more recent tendency on the way to better swarm intelligence is to include self-learning mechanisms which can dynamically control various search strategies. Yang et al.^52^ suggested the Self-Learning Salp Swarm Algorithm (SLSSA) that combines four separate search strategies into a probability-based adaptive framework. The execution of every search strategy in SLSSA is updated with the help of a reward system where the extent of fitness improvement in the previous steps is measured. In order to increase the exploration ability, the algorithm adds multiple food source search strategy based on the attraction dynamics of PSOs, and generalized oppositional learning to enrich population diversity. The benchmark suite of experimental validation of the CEC2014 showed better convergence accuracy and stability than some state-of-the-art optimizers. However, even with adaptive self-learning mechanism, SLSSA takes the probabilistic direct learning scheme instead of a reinforcement-based decision framework and does not involve cooperative multi-agent learning and adaptive policy switching depending on population states. This encourages more intelligent reinforcement-based hybrid frameworks to be developed like the proposed DMARS_WGO.

In this study, two reinforcement-driven hybrid metaheuristic approaches are proposed to improve the performance of WO and GOA. The first model, AIRE_WGO, introduces a Q-learning^53^ based control policy to dynamically adjust the trade-off between exploration and exploitation balance. With this integration, parameter tuning will become adaptive, and convergence will become more stable in a wide range of optimization problems. On the same basis, a second model, DMARS_WGO, further develops the reinforcement mechanism by integrating a dual agent learning setup that integrates conventional Q-learning with a Deep Q Network (DQN)^54^. This dual-mode design allows adaptive switching between discrete and continuous learning modes according to contextual feedback such as population diversity, improvement rate, and stagnation.

Extensive comparisons with AIRE_WGO, WO, GOA, nine additional 9 state-of-the-art algorithms, namely Golden Jackal Optimization (GJO)^26^, Osprey Optimization Algorithm (OOA)^27^, Pelican Optimization Algorithm (POA)^28^, Rat Swarm Optimizer (RSO)^55^, Smell Agent Optimization (SAO)^56^, Channa argus optimizer (CAO)^57^, Rüppell’s Fox Optimizer (RFO)^58^, Mantis Shrimp Optimization Algorithm (MShOA)^29^, and Adaptive L-SHADE algorithm (ALSHADE)^30^ demonstrate that DMARS_WGO achieves superior robustness, scalability, and convergence efficiency across benchmark and engineering design problems.

## Research gap

Although nature-inspired and hybrid metaheuristic optimizers are rapidly evolving, most current methods are essentially controlled by a fixed set of search equations or fixed schedules of parameters. Even the adaptive variants do not tend to maintain the dominance of the operators of exploration and exploitation structurally, but often control the scalar coefficients. This weakness often leads to premature convergence, oscillatory switching, or poor adaptation to search in very multimodal and high-dimensional landscapes.

In spite of the recent introduction of reinforcement learning-assisted optimizers in order to promote adaptability, the majority of them are based on single-agent learning mechanisms that optimize parameters or choose operators without mutual interaction of the learning paradigms of heterogeneous agents. Also, state abstraction based on diversity consciousness and knowledge transfer between agents are poorly studied among existing RL-based optimization methods.

This means that the field does not have a cooperatively dual-mode reinforcement architecture that can dynamically control structural search behavior and be stable and robust over a variety of optimization landscapes.

## Research motivation

In the complex engineering optimization problems, converting the exploration and exploitation balance to be reliable and adaptive is critical and in the unstable case the design might not be optimal, the cost of operation might be high or the system might be underperforming. Optimization problems become more dimensional and structurally complex, and the demands of search strategies to be static and loosely adaptive fail to achieve robustness under different search phases. Thus, there is a high demand of a framework of optimization that would automatically control its search dynamics based on real-time feedbacks of the population state. This is inspiring the creation of dual-mode reinforcement-based evolutionary architecture that unites complementary learning paradigms to improve adaptability, stability, and scalability in harmful optimization settings. The classical metaheuristic algorithms usually use constant or heuristically determined mechanisms as a measure of exploration and exploitation. Nonetheless, those methods are not responsive to changing search conditions and can lead to untimely convergence or ineffective exploration. Reinforcement learning offers a principled framework of adaptive control that can make context-sensitive decisions informed by real time population feedbacks. Thus, RL enables autonomous behavioral control in the optimization procedure to enhance adaptability and stability in the search environments of high complexity.

The main contributions of the proposed algorithms can be summarized as:

1. Using Q-learning through $\varepsilon$ greedy policy and deep Q-learning to balance exploration and exploitation.
2. Proposing a modified algorithm AIRE_WGO through introducing adaptive parameters $\beta \left( t \right),\phi \left( t \right),\eta \left( t \right)$ to gazelle exploration equations rather than constant parameters and the exploitation phase is enhanced by combining WO and GOA.
3. Proposing another modified algorithm DMARS_WGO which introduces a dual-mode reinforcement mechanism that allows the algorithm to dynamically alternate between tabular Q learning and deep neural Q-value estimation (DQN) depending on the search space to balance exploration and exploitation and improve convergence behavior in complex optimization problems.
4. Using an adaptive mutation and soft-switching strategy that sustains population diversity and prevents premature convergence throughout the optimization process.

The remainder of this paper is organized as follows. Section “Research Gap” provides a literature review of the state-of-the-art algorithms. Section “Background” presents the background of WO and GOA. Section “The proposed work” presents the mathematical formulation and workflow of the proposed AIRE_WGO and the proposed DMARS_WGO algorithm. Section “Experimental results and analysis” discusses the simulation results of DMARS_WGO and AIRE_WGO as compared to other algorithms on several benchmarks. Section “Conclusion and Future Work” shows the conclusion of the work and future work.

## Related work

The ability of metaheuristic optimization to resolve complex, nonlinear, and multimodal problems has garnered a lot of interest in recent years. Osprey Optimization Algorithm (OOA)^27^, Rat Swarm Optimizer (RSO)^55^, Smell Agent Optimization (SAO)^56^, and Rüppell’s Fox Optimizer (RFO)^58^ are just a few of the bio-inspired algorithms that have been created with the goal of finding a balance between exploration and exploitation. Despite their impressive performance on benchmark and real-world problems, these algorithms frequently exhibit similar drawbacks, such as susceptibility to parameter adjustment, premature convergence, and stagnation in local optima. This section reviews related works on other state-of-the-art algorithms.

According to Gaurav Dhiman et al.^55^ Rat Swarm Optimizer (RSO) is inspired by the chasing and fighting behaviors of rats. The algorithm incorporates two main techniques: the chasing technique and the fighting technique. These behaviors are adaptively controlled by two parameters, enabling a balance between diversification and intensification throughout the optimization process. RSO achieves competitive results compared to well-known algorithms such as PSO, GWO, and GA.

According to Salawudeen et al.^56^ the Smell Agent Optimization (SAO) algorithm is described as a bio-inspired metaheuristic that mimics the behavior of biological agents, the agents are likely to detect smells and follow the movement of the diffusing smoke molecules. The algorithm represents three important behavioral modes that entail sniffing, trailing, and random. When sniffing, the agents identify the odorant molecules that are diffused as the laws of Brownian motion and calculate the new positions on the basis of the equations of kinetic velocity of the mass and temperature, to guarantee successful global exploration. During the trailing phase, all agents control the appropriate attraction of the best and repulsion of the less promising individuals in an adaptive way by using an olfaction coefficient in order to enter zones with higher odor concentration in the trailing phase to achieve a balance between the exploration and exploitation activities. The random mode, which adds random step length (SL) perturbations that re-diversify the population and help in overcoming local optima, takes place when the agents lose the odor trail. This algorithm generates a naturally dynamic balance between intensification and diversification by simulating odor diffusion through temperature, mass of molecules and kinetic energy.

According to Pavel Trojovsky et al.^28^ Pelican Optimization Algorithm (POA) simulates the coordination behavior of pelicans when hunting and catching prey. With a stochastic movement equation, every pelican selects a random location of the prey during exploration and adjusts its position in the direction of the prey. After the identification of potential prey sites, the algorithm proceeds to the exploitation step, in which the pelicans narrow down their paths in response to the direction of a diminishing search radius. It is an adaptive term that dynamically decreases the step size of search as the iterations approach one another, creating a smooth balance between exploration and exploitation. Using adaptive search contraction, stochastic motion control, and random prey generation, POA can attain healthy optimization convergence and can avoid early convergence effectively.

According to Nitish Chopra et al.^26^ the Golden Jackal Optimization (GJO) algorithm, which is a metaheuristic that mimics the hunting behavior of the golden jackal in cooperation. The algorithm employs the adaptive regulation of the evasive energy and the escape energy coefficient to replicate the two major stages, i.e., exploration and exploitation. As the evasive energy is comparatively high, it is the stage of exploration, when the jackals search a large area by changing their locations. The algorithm enters the stage of exploitation when the energy of the evasion drops. At this stage jackals optimize their positions, surround the prey, and attack it. The two most superior solutions in each generation direct the rest of the population to the best prey locations. The GJO would get good results as compared to PSO, GWO, WOA and other algorithms.

According to Yintong Li et al.^30^state that Adaptive L-SHADE (AL-SHADE) algorithm is a better version of Success-History based Adaptive Differential Evolution (L-SHADE). The algorithm takes the current-to-Amean/1 and current-to-pbest/1 method as complementary to each other and thus should be used simultaneously to provide a dynamic balance between the exploration and exploitation phases. The latter operator assists to guide the search to the promising regions but maintain the diversity of the population by the weighted mean of elite individuals that are stored in an external archive. Probabilistic control mechanism is an adaptive parameter PSP that determines the method that is used at every iteration and depends on their success rates in the past. Due to this adaptive switching, the algorithm is able to start with exploration in the initial iterations, and progressively do more local refinement as the algorithm nears convergence.

According to Mohammad Dehghan et al.^27^ the Osprey Optimization Algorithm (OOA) is a bio-inspired metaheuristic that is based on the intelligent hunting process of ospreys in the wild. At the exploration stage, ospreys employ stochastic flight equations to revise their locations and select a target randomly to search the area diversely and locate prey (fish). In the exploitation stage, the algorithm applies adaptive movement equations to dynamically adjust positions to the best solutions, which is analogous to precision diving and catching the prey used by the osprey. The two-phase methods enable OOA to effectively trade off between exploration and exploitation and to attain high convergence accuracy and stability.

According to Braik and Al-Hiary^58^ Rüppell’s Fox Optimizer (RFO) is a recent swarm-based metaheuristic inspired by the communal foraging behavior of Rüppell’s foxes in desert habitats and mountainous. The algorithm simulates five primary hunting patterns that include hearing-based localization, sight-based search, smell-based tracking, and rotational sensory motions of ears and eyes. Each solution is updated using stochastic movement equations, which incorporate random prey generation as well as time-varying sensory weighting. RFO is a combination of a cognitive coefficient and an adaptive term that is used to control the shrinking search radius. With such adaptive techniques that combine radius contracting, circular rotations updates, and sensory-based randomization, RFO is able to escape local optima.

According to Sánchez Cortez et al.^29^ state that the Mantis Shrimp Optimization Algorithm (MShOA) is a bio-inspired metaheuristic which simulates the extraordinary visual perception and multimodal hunting behavior of mantis shrimps. The algorithm is a mixture of three behavioral phases; foraging, attacking, and defending. The phases are governed by stochastic equations of motion which model Brownian and circular dynamics. The Polarization Type Identifier (PTI) is an imitation of the polarized vision of the shrimp to alterively switch between the two phases in response to environmental conditions, which regulates the tradeoff between exploration and exploitation. The algorithm is adaptive because it will automatically adjust its behavioral strategy based on the feedback of the search process by including an adaptive mechanism based on perception other than relying on a set of predefined control parameters. This design improves population diversity, maintains a strong balance between exploration and exploitation, and avoids premature convergence.

Azizi et al. introduced Fire Hawk Optimizer (FHO)^59^ which is nature-inspired metaheuristic, grounded on foraging behavior of fire hawks, and confirmed its efficacy on large benchmark functions and CEC 2020 real-world optimization problems. Ghasemi et al. proposed the Kirchhoff Law Algorithm (KLA)^60^, a physics-inspired, which uses Kirchhoff Law, and where solution interactions are represented by electrical currents flowing through resistance-defined branches. Dehghani et al. introduced Coati Optimization Algorithm (COA)^61^ a bio-inspired population-based metaheuristic that represents the hunting and escaping behavior of coatis, and using separate exploration and exploitation stages. Wang et al. introduced Octopus Optimization Algorithm (OOA)^62^ that simulates the octopus behaviors during hunting and mating by using a recoil based stochastic feedback system and multi-range exploitation strategies.

Recent studies demonstrate on ongoing developments of adaptive, hybrid, and application-oriented metaheuristic algorithms with a focus on structural improvement, learning incorporation, and a better optimization output in a wide variety of problems^63–69^.

## Background

This section explains briefly the standard algorithms Walrus Optimizer (WO)^49^ and Gazelle Optimization Algorithm^50^ which are the behavioral and mathematical core of the proposed AIRE_WGO and DMARS_WGO algorithms.

### Walrus optimizer (WO)

WO is one of the recent optimization algorithms that simulate the behavior of the walrus. It was introduced by Muxuan Han et al.^49^ in 2024. The walruses are the largest marine mammals other than whales. They live mainly in temperate seas in or close to the Arctic^70^. The life cycle of the walrus consists of phases: migrating, breeding, roosting, and foraging^71^. The exploration and exploitation depend on 2 signals: The Danger signal and the Safety signal^49^. If the danger signal is greater than or equal to 1, Herds of walruses will move to places more suited for population survival. The walrus position is updated in this phase (Migration) in the manner described by Eq. 1:

1 $$ X_{i,j}^{t + 1} = X_{i,j}^{t} + Migration\_step $$

2 $$ Migration\_step = \left( {X_{m}^{t} - X_{n}^{t} } \right) \cdot \beta \cdot r_{3}^{2} $$

3 $$ \beta = 1 - \frac{1}{{1 + {\mathrm{exp}}\left( { - \frac{{t - \frac{T}{2}}}{T} \times 10} \right)}} $$

where $$Migration\_step$$ is the walrus movement’s step size, $$X_{i,j}^{t}$$ represents its existing location on the $$j$$th dimension, $$X_{i,j}^{t + 1}$$ represents the $$i $$ th walrus’s new location on the $$j$$th dimension. Two vigilantes are randomly selected from the population, and the positions of the vigilantes correspond to $$X_{m}^{t}$$ and $$X_{n}^{t}$$, $$\beta$$ is the migration step control factor, which changes as a smooth curve with each iteration; $$r_{3}$$ is a random number that falls between 0 and 1; $$T$$ is the total iterations, and $$t$$ is the current iteration.

Herds of walruses typically breed in currents with low danger factors. The two primary actions during reproduction are underwater foraging and onshore roosting. If the safety signal is more than 0.5, in this phase of roosting, the male walrus updates their position by using the Halton sequence method^72^. Female walruses are influenced by the lead walrus ($X_{best}^{t}$) and male walruses ($male_{i,j}^{t}$). The female walrus is progressively affected more by the leader and less by the male over the iteration phase as described by Eq. 4:

4 $$ female_{i,j}^{t + 1} = female_{i,j}^{t} + \alpha \cdot \left( {male_{i,j}^{t} - female_{i,j}^{t} } \right) + \left( {1 - \alpha } \right) \cdot \left( {X_{best}^{t} - female_{i,j}^{t} } \right) $$

where $$male_{i,j}^{t}$$ and $$female_{i,j}^{t} $$ are the positions of the $$i$$ th male and female walruses on the $$j$$ th dimension, $$female_{i,j}^{t + 1} $$ donates the new position for the $$i$$ th female walrus on the $$j$$ th dimension, $$\alpha$$ parameter decreases from 1 first to 0 with the number of iterations $$t$$.

Juvenile walruses update their current position to avoid predators based on $L\mathop e\limits^{\prime } vy$ distribution as illustrated by Eq. 5:

5 $$ Juvenile_{i,j}^{t + 1} = \left( {o - Juvenile_{i,j}^{t} } \right) \cdot P $$

6 $$ o = X_{best}^{t} + Juvenile_{i,j}^{t} \cdot LF $$

where$$Juvenile_{i,j}^{t} $$ is the position of the $$i$$ th juvenile walrus on the $$j$$ th dimension, $$Juvenile_{i,j}^{t + 1}$$ is the new position for the $$i$$th juvenile walrus on the $$j$$ th dimension, $$o$$ is the reference safety position, $$P$$ is the distress coefficient of juvenile walrus and is a random number of (0,1), $$LF$$ is a vector of random numbers based on $$L\mathop e\limits^{\prime } vy$$ distribution representing $$L\mathop e\limits^{\prime } vy$$ movement represented by Eq. 7:

7 $$ L\mathop e\limits^{\prime } vy\left( a \right) = 0.05 \times \frac{x}{{\left| y \right|^{\frac{1}{a}} }} $$

where $x$ and $y$ are two variables with normal distributions: $x\sim N\left( {0,\sigma_{x}^{2} } \right)$ and $y\sim N\left( {0,\sigma_{y}^{2} } \right)$ where:

8 $$ \sigma_{x} = \left[ {\frac{{\Gamma \left( {1 + \alpha \sin \left( {\frac{\pi \alpha }{2}} \right)} \right)}}{{ \Gamma \left( {\frac{1 + \alpha }{2}} \right)\alpha 2^{{\frac{{\left( {\alpha - 1} \right)}}{2}}} }}} \right]^{\frac{1}{a}} ,\,\,\sigma_{y} = 1, \alpha = 1.5 $$

where $\Gamma \left( x \right) = \left( {x + 1} \right)$ and $\sigma_{x}$ and $\sigma_{y}$ are the standard deviations.

During underwater foraging, walruses are also assaulted by natural predators. If their peers warn them of danger, they will leave their current area and update their position as regarded by Eq. 9:

9 $$ X_{i,j}^{t + 1} = X_{i,j}^{t} \cdot R - \left| {X_{best}^{t} - X_{i,j}^{t} } \right| \cdot r_{4}^{2} $$

where $\left| {X_{best}^{t} - X_{i,j}^{t} } \right|$ represents the distance between the current walrus and the best walrus, $r_{4}$ is a random number that falls between 0 and 1, R is a danger factor.

All of the walrus herd may locate the sea area with a higher food abundance by exchanging location information, and walruses can work together to forage and move in line with the locations of other walruses in the community as Eq. 10:

10 $$ X_{i,j}^{t + 1} = \left( {X_{1} + X_{2} } \right)/2 $$

11 $$ \left\{ {\begin{array}{*{20}l} {X_{1} = X_{best}^{t} - a_{1} \times b_{1} \times \left| {X_{best}^{t} - X_{i,j}^{t} } \right|} \hfill \\ {X_{2 = } X_{second}^{t} - a_{2} \times b_{2} \times \left| {X_{second}^{t} - X_{i,j}^{t} } \right|} \hfill \\ \end{array} } \right. $$

12 $$ a = \beta \times r_{5} - \beta $$

13 $$ b = \tan \left( \theta \right) $$

where the two weights $$X_{1}$$ and $$X_{2}$$ influence the walrus's gathering behavior, $$X_{second}^{t}$$ indicates where the second walrus is in the current iteration, and $$\left| {X_{second}^{t} - X_{i,j}^{t} } \right|$$ indicates the separation between the current walrus and the second walrus. The gathering coefficients for the second walrus are $$a$$ and $$b$$, $$r_{5} $$ is a random number between 0 and 1, and θ accepts values between 0 and π.

Algorithm 1 illustrates the pseudo-code^49^.

**Algorithm 1:**
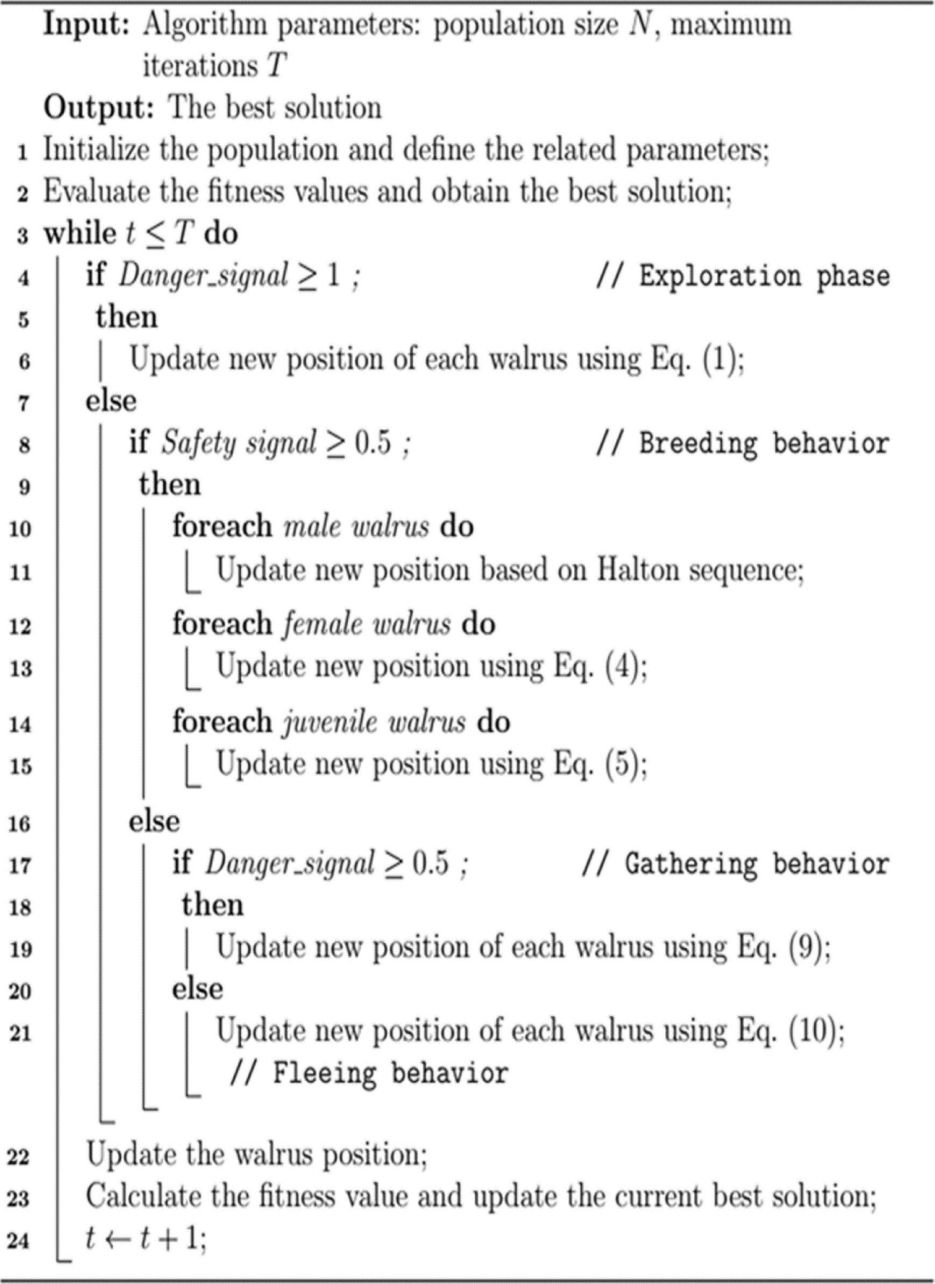
Pseudo-code of Walrus optimization algorithm (WOA)

### Gazelle optimization algorithm (GOA)

GOA is one of the recent optimization algorithms that simulates how gazelles might behave in order to survive. It was introduced by Jeffrey O. Agushaka et al.^50^ in 2022. The gazelles live in the arid regions that range throughout the Arabian Peninsula, sections of Asia, including China, and the northern African Sahara desert. The life cycle of a gazelle consists of two phases, which are running from a sighted predator to a haven and grazing in the absence of a predator; both are parts of the optimizing process.

In the exploitation phase, it is assumed that the gazelles are either stalked by a predator or are grazing contentedly in the absence of a predator. During this phase, neighborhood portions of the domain were efficiently covered using Brownian motion^73^, which is characterized by regular and controlled steps. When grazing, it is supposed that the gazelles move in a Brownian motion as in Eq. 14:

14 $$ gazelle_{i + 1} = gazelle_{i} + s.R*.R_{B} *.\left( {Elite_{i} - R_{B} *.gazelle_{i} } \right) $$

where $$gazelle_{i + 1}$$ is the next iteration’s solution, $$gazelle_{i}$$ is the current iteration’s solution, $$s $$ is the grazing speed of the gazelle, $$\vec{R}_{B} $$ is a vector containing random numbers representing the Brownian motion, $$R $$ is the vector of uniform random numbers [0,1], $${\mathrm{Elite}}_{{\mathrm{i}}}$$ is a matrix which constructed by top gazelles and used for searching and finding the next step for the gazelles.

The exploration phase starts at the moment a predator is seen. When the predator is spotted, the gazelle flees, and the predator follows. The sudden turn in direction that characterizes both runs is $\mu$. The gazelle responds first, so it uses Levy flight^74^. The predator responds later, so it runs using the Brownian motion, then changes the motion to the Levy flight. Equation 15 illustrates the gazelle's behavior when it detects the predator.

15 $$ \overrightarrow {{gazelle_{i + 1} }} = \overrightarrow {{gazelle_{i} }} + S.\mu .\vec{R}*.\vec{R}_{L} *.\left( {\overrightarrow {{Elite_{i} }} - \overrightarrow {{R_{L} }} *.\overrightarrow {{gazelle_{i} }} } \right) $$

where $S$ is the fastest speed the gazelle can get, $\vec{R}_{L}$ is a vector of random numbers based on Levy distributions.

Equation 16 illustrates the predator's behavior as it chases the gazelle.

16 $$ \overrightarrow {{gazelle_{i + 1} }} = \overrightarrow {{gazelle_{i} }} + S.\mu .CF* \cdot \vec{R}_{B} * \cdot \left( {\overrightarrow {{Elite_{i} }} - \overrightarrow {{R_{L} }} * \cdot \overrightarrow {{gazelle_{i} }} } \right) $$

where $CF = \left( {1 - \frac{iter}{{Max\_iter}}} \right)^{{\left( {2\frac{iter}{{Max\_iter}}} \right)}}$ indicates the predator’s cumulative effect.

In this algorithm, it is supposed that gazelles had an annual survival rate of 0.66, meaning that predators only succeed in 0.34 of these cases^75^. Predator success rates (PSRs) have an effect on the gazelle's capacity to flee. Equation 17 models the PSRs effect.

17 $$ \overrightarrow {{gazelle_{i + 1} }} = \left\{ {\begin{array}{*{20}l} {\overrightarrow {{gazelle_{i + 1} }} + CF\left[ {\overrightarrow {LB} + \vec{R}*.\left( {\overrightarrow {UB} - \overrightarrow {LB} } \right)} \right]*.\vec{U}} \hfill & {if r \le PSRs} \hfill \\ {\overrightarrow {{gazelle_{i + 1} }} + \left[ {PSRs\left( {1 - r} \right) + r} \right]\left( {\overrightarrow {{gazelle_{r1} }} - \overrightarrow {{gazelle_{r2} }} } \right)} \hfill & {otherwise} \hfill \\ \end{array} } \right. $$

where $\vec{U}$ is a binary vector that is created by producing a random number r in [0, 1] such that

$$ \vec{U} = \left\{ {\begin{array}{*{20}l} {0,} \hfill & {if\,\, r < 0.34} \hfill \\ {1,} \hfill & {otherwise} \hfill \\ \end{array} } \right. $$

$r_{1}$ and $r_{2}$ are random indices of the gazelle matrix.

The pseudo code of GOA is described in Algorithm 2^50^.

**Algorithm 2:**
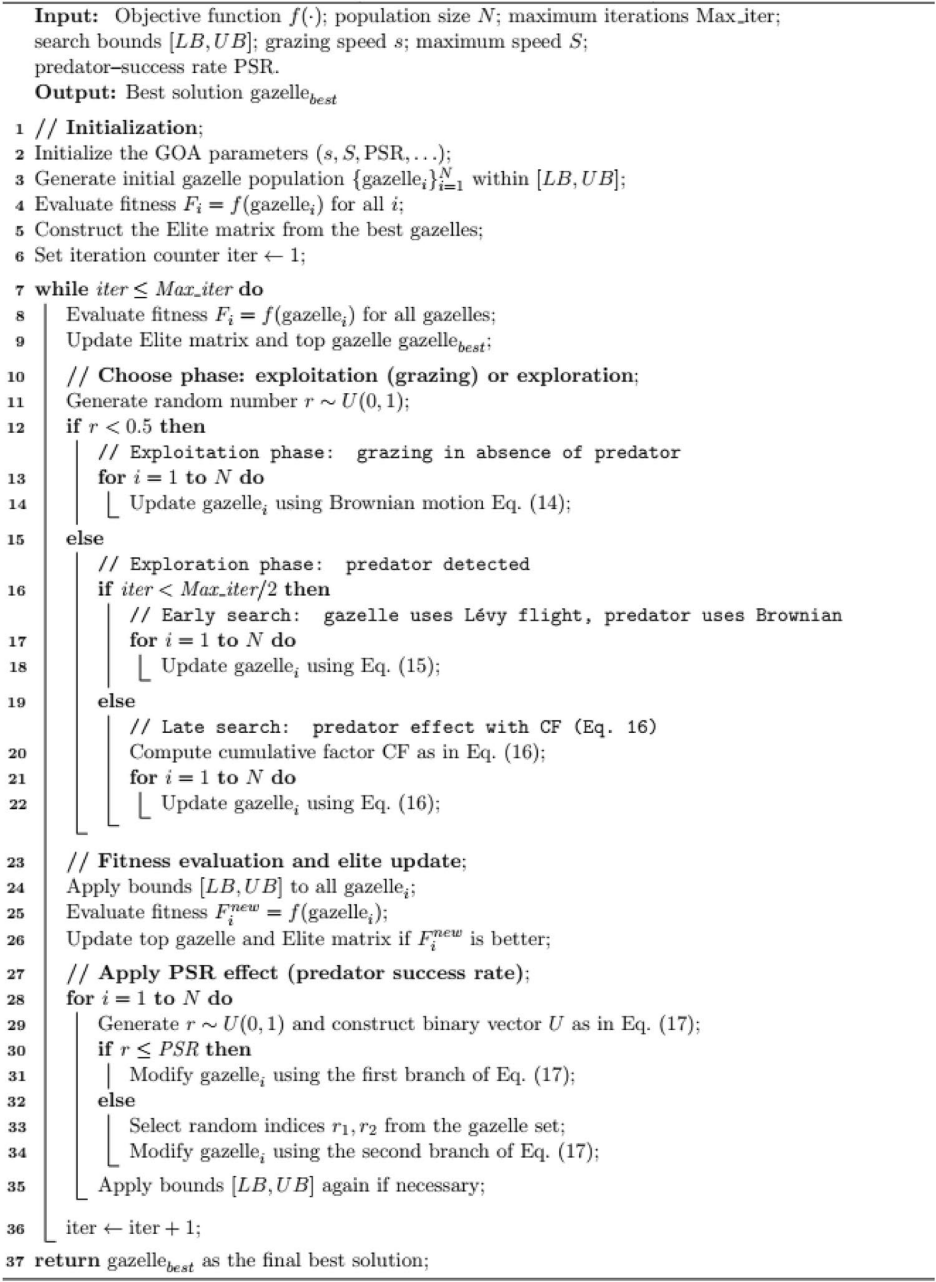
Pseudo code of GOA (Gazelle optimization Algorithm)

## The proposed work

The initial version of WO and GOA had some drawbacks, such as the slow convergence speed and a probability for getting trapped in local optima. Exploration and exploitation of the search space must be balanced to avoid premature convergence. However WO demonstrates strong local exploitation and GOA exhibits excellent global exploration, some adjustments have been introduced to both algorithms claiming the improvement of the previously mentioned drawbacks.

### Proposed 1: adaptive intelligent reinforced Walrus_Gazelle optimizer (AIRE_WGO)

**Fig. 2:**
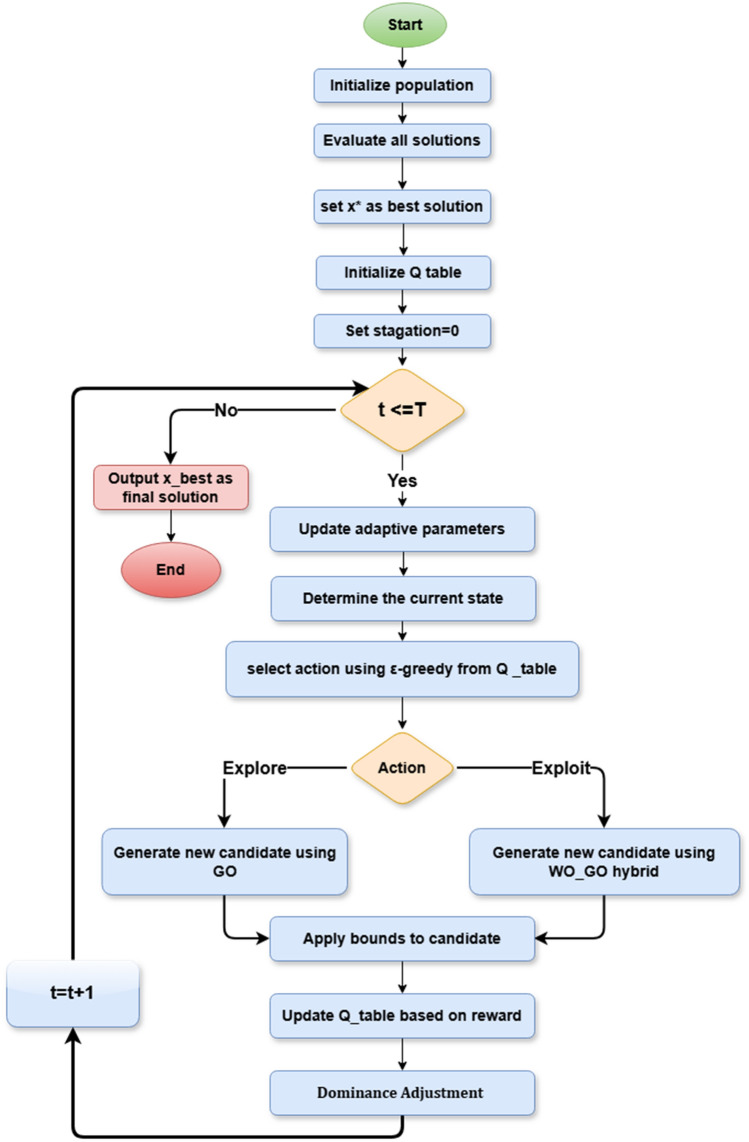
The flowchart of the proposed algorithm AIRE_WGO.

The Adaptive Intelligent Reinforced Walrus_Gazelle Optimizer (AIRE_WGO) is a hybrid reinforcement-driven metaheuristic framework designed to achieve an intelligent balance between exploration and exploitation through adaptive behavioral learning. Figure 2 shows the flowchart of the AIRE_WGO algorithm, starting with population initialization and fitness evaluation. It combines Q learning to adaptively select between exploration of GOA or exploitation of hybrid WO and GOA through a $\varepsilon$ greedy policy. The flowchart also shows adaptive parameter tuning and stagnation handling to maintain diversity and guide convergence efficiently. The pseudo code is described in Algorithm 3. Details of the proposed algorithm are explained in the next sub-sections.

**Algorithm 3:**
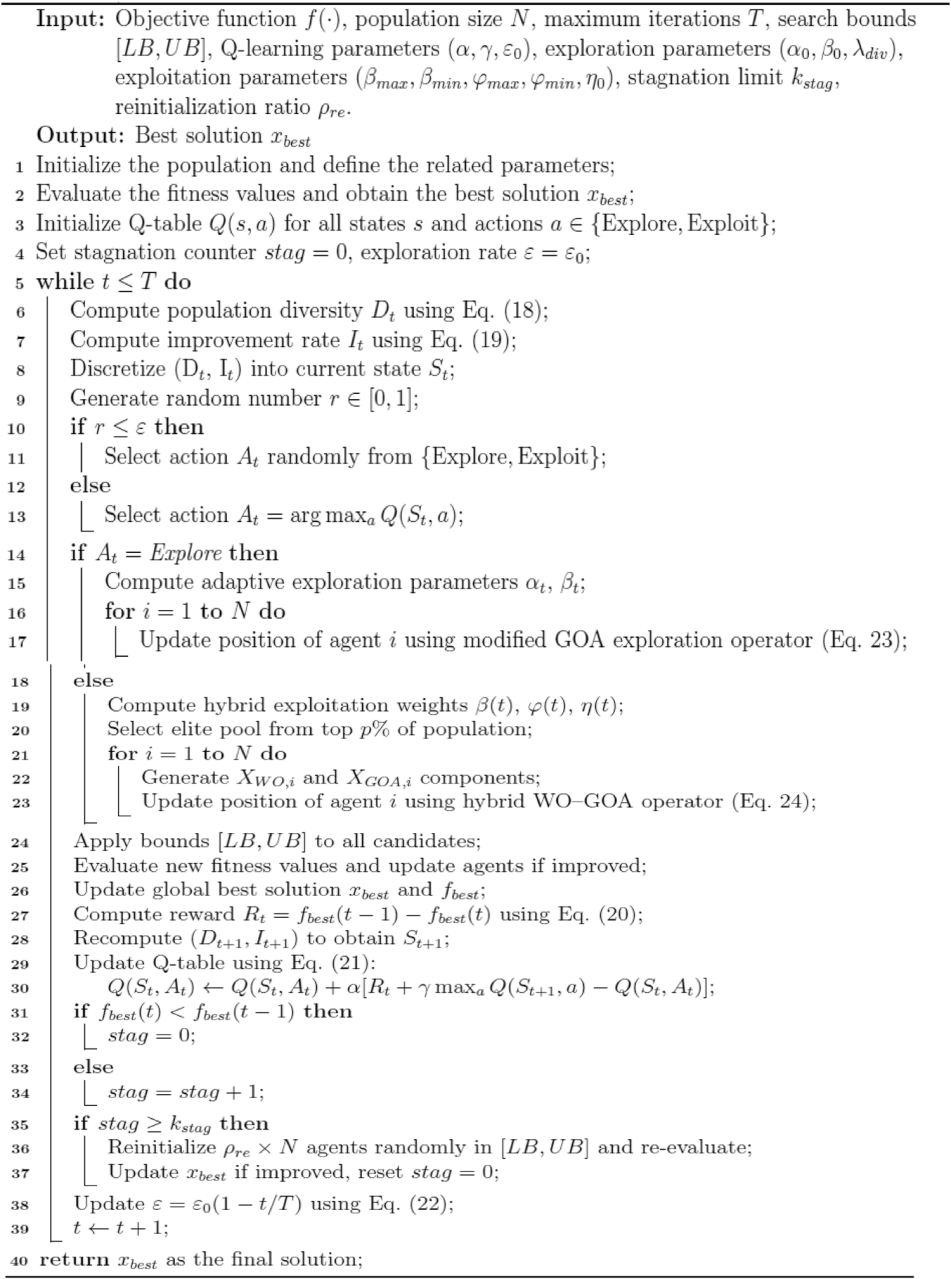
Pseudo-code of AIRE-WGO (Adaptive Intelligent Reinforced Walrus-Gazelle Optimizer)

#### Q learning table

In the proposed algorithm, the Q learning table is used to direct the decision-making process using a reinforcement learning mechanism. The Q table is intended to translate several states of the search process into the optimal action. These include population diversity and improvements in the best fitness and are binned into discrete levels. The diversity of populations ${\mathrm{D}}_{{\mathrm{t}}}$ shows how dispersed the individuals are around the population mean and is computed as in Eq. 18:

18 $$ D_{t} = \frac{{std\left( {P_{t} } \right)}}{{mean\left( {P_{t} } \right) + \varepsilon }} $$

where $P_{t}$ is the population at t.

The improvement rate ${\mathrm{I}}_{{\mathrm{t}}}$ quantifies the relative enhancement in the global best fitness ${\mathrm{f}}_{{{\mathrm{best}}}}$ between consecutive iterations and is computed as in Eq. 19:

19 $$ I_{t} = \frac{{\left| {f_{best} \left( t \right) - f_{best} \left( {t - 1} \right)} \right|}}{{\left| {f_{best} \left( {t - 1} \right) + \varepsilon } \right|}} $$

Each state $S_{t} = \left[ {{\mathrm{D}}_{{\mathrm{t}}} , {\mathrm{I}}_{{\mathrm{t}}} } \right]$ in the table represents the system's current state, and the two actions decide whether the algorithm should improve the current solutions (exploitation) or seek out new ones (exploration). The reward function is calculated as in Eq. 20:

20 $$ R_{t} = f_{best} \left( {t - 1} \right) - f_{best} \left( t \right) $$

where $f_{best} \left( {t - 1} \right)$ represents the best fitness value at the previous iteration and $f_{best} \left( t \right)$ represents the best fitness value at the current iteration.

The Q values are updated iteratively according to the rewards received, as in Eq. 21. This technique allows the algorithm to balance between discovering different areas of the search space and improving the quality of the best solutions.

21 $$ Q_{new} \left[ {S_{t} ,A_{t} } \right] = Q\left[ {S_{t} ,A_{t} } \right] + \alpha \times \left[ {R_{t} + \gamma maxQ\left( {S_{t + 1} ,a} \right) - Q\left( {S_{t} ,A_{t} } \right)} \right] $$

where ${\mathrm{Q}}_{{{\mathrm{new}}}} \left[ {{\mathrm{S}}_{{\mathrm{t}}} ,A_{{\mathrm{t}}} } \right]$ is the updated Q-value, $Q\left[ {S_{t} ,A_{t} } \right]$ is the current Q-value for state $S$ and action $A$, ${\upalpha }$ is the learning rate between 0 and 1, ${\mathrm{R}}_{{\mathrm{t}}}$ is the immediate reward received after taking action $A$ in state $S$, ${\upgamma }$ is The discount factor a number range in [0,1], ${\mathrm{maxQ}}\left( {{\mathrm{S}}_{{{\mathrm{t}} + 1}} ,{\text{ a}}} \right)$ is the maximum Q-value for all possible actions $a$ in the next state $S_{t + 1}$, $S_{t + 1}$ is The new state reached after taking action $A$ in state $S$, $a$ combines all possible actions in the new state $S_{t + 1}$.

Q learning uses epsilon-greedy $\varepsilon$ to effectively balance the crucial trade-off between exploitation and exploration. At each iteration, a random number is produced and compared with $\varepsilon$ . If this random number is less than or equal to $\varepsilon$, the algorithm does a random action (exploration or exploitation), to encourage diversity and prevent the search from getting trapped in local optima. If the random number exceeds $\varepsilon$, the algorithm selects the action with the highest estimated value from the Q-table for the current state.$\varepsilon$ is set to decay over time to provide a smooth transition from global search to local refinement. As the number of iterations increases, $\varepsilon$ gradually decreases as in Eq. 22. By using this decay technique, the proposed algorithm is able to achieve both robust global search and effective convergence by dynamically balancing diversification and intensification. In the experiments, the initial value of $\varepsilon_{0}$ was set to 0.1.

22 $$ \varepsilon = \varepsilon_{0} \times \left( {1 - \frac{t}{T}} \right) $$

where $\varepsilon_{0}$ is the initial epsilon greedy, $t$ is the current iteration, and $T$ is the maximum number of iterations.

#### The modification of AIRE_WGO algorithm (exploration phase)

In AIRE_WGO, the exploration operator was originally derived from the GOA; all motion and exploration parameters in the original formulation of GOA are defined as static and non-adaptive constants, meaning they don’t change during the optimization process, regardless of the search dynamics. This inflexibility limits the algorithm's ability to respond to changes in the search environment, particularly in complex or high-dimensional environments. As a result, the algorithm may suffer from limited exploration capability and an increased tendency to become trapped in local optima. However, to overcome these limitations, improve search efficiency, and avoid premature convergence, the operator was modified by incorporating adaptive scaling parameters, Lévy flights, and a Gaussian Cauchy mutation mixture, resulting in a more diversity-aware and flexible exploration method. Equation 23 defines the proposed exploration in AIRE_WGO.

23 $$ {\mathrm{X}}_{{\mathrm{i}}}^{{{\mathrm{t}} + 1}} = {\mathrm{X}}_{{\mathrm{i}}}^{{\mathrm{t}}} + {\upalpha }_{{\mathrm{t}}} {\text{*mu* CF RB * }}\left( {{\upvarepsilon }_{{\mathrm{t}}} { } - {\text{ RL }}.{\text{* xi}}} \right) + 0.1{\mathrm{*L}}\mathop {\mathrm{e}}\limits^{\prime } {\mathrm{vy}}\left( {\uplambda } \right) + {\upbeta }_{{\mathrm{t}}} {\mathrm{*M}}_{{\mathrm{i}}}^{{\mathrm{t}}} $$

where $$ X_{i}^{t + 1}$$ and $$X_{i}^{t}$$ donate the position of the $$i$$ th candidate at iterations $$t + 1 $$ and $$t$$, $$mu = 2*rand - 1$$ is the mutation factor providing a random direction in the range [− 1,1], The cumulative predator effect is expressed as $$CF = \left( {1 - \frac{iter}{{Max_{iter} }}} \right)^{{\left( {2\frac{iter}{{Max_{iter} }}} \right)}} $$ which controls the decay of exploratory steps as the iterations increase, $$RB$$ is a vector containing random numbers representing the Brownian motion, $$RL$$ denotes a vector of random numbers based on $$L\mathop e\limits^{\prime } vy$$ distributions^76^, $$\varepsilon_{t}$$ represents a random elite pool sampled from the best-performing individuals at iteration $$t$$, $$ { }0.1{*}L\mathop e\limits^{\prime } vy\left( \lambda \right)$$ introduces occasional large steps to escape local optima. A mutation mixture is also incorporated by the operator $$M = \left( {1 - \rho } \right)*\mu \_G + \rho *\mu \_C,$$ which combines Gaussian adaptive mutation $$\mu_{G} $$ and Cauchy adaptive mutation $$\mu_{C}$$ with a random mixing coefficient $$ \rho$$.

$\alpha_{t }$ and $\beta_{t}$ are adaptive scaling parameters regulate the influence of exploration and mutation, respectively. The population diversity is measured as $diversity = population diversity\left( X \right)$, which is normalized into the range [0, 1] using a soft scaling function $div\_norm = diversity/\left( {diversity + 1} \right)$ Based on this normalization,$\alpha_{t }$ is defined as $\alpha_{t} = \alpha_{0} *\left( {1 - t/T^{2} * \left( {1 + 0.5*div\_norm} \right)} \right)$ which ensures that the exploration step decreases gradually over iterations and more rapidly when population diversity is high. Similarly, $\beta_{t}$ is computed as $\beta_{t} = {\upbeta }_{0} *\left( {1 + \lambda \times div\_norm} \right)$ where $\lambda$ = 0.3 is the diversity scaling parameter. With this formulation $\beta_{t}$ adaptively grows with variety, permitting more intense disturbances in more distributed populations.

These features guarantee that AIRE_WGO's exploration is both adaptive and diversity-aware, allowing the algorithm to explore widely in its initial iterations and progressively focus as convergence is approached.

#### The modification of AIRE_WGO algorithm (exploitation phase)

The exploitation phase of the proposed AIRE_WGO algorithm is formulated as a hybrid of the Walrus Optimizer (WO) and the Gazelle Optimization Algorithm (GOA) as defined in Eq. 24:

24 $$ X_{i}^{t + 1} = X_{i}^{t} + \beta \left( t \right)X_{WO}^{t} + \phi \left( t \right)X_{GOA}^{t} + \eta \left( t \right)\varepsilon^{t} $$

where ${\mathrm{X}}_{{{\mathrm{WO}}}}^{{\mathrm{t}}}$ corresponds to the formulations already described in Eq. 4 and ${\mathrm{X}}_{{{\mathrm{GOA}}}}^{{\mathrm{t}}}$ corresponds to the formulations already described in Eq. 14, three adaptive coefficients ${ }\beta \left( t \right)$, $\phi \left( t \right),$ and $\eta \left( t \right)$ determine the relative contributions of the WO, GOA, and elite jitter, respectively.

WO weight ${ }\beta \left( t \right)$ is defined as $\beta \left( t \right) = \beta_{max} - \left( {\beta_{max} - \beta_{min} } \right) \times \frac{t}{T}$. This parameter regulates the contribution of walrus-inspired behavioral dynamics (fleeing and gathering). It decreases linearly from $\beta_{max}$ at the start of the search to $\beta_{min}$ at the final iteration. A large initial value of $\beta \left( t \right)$ encourages a stronger influence of WO’s behavioral dynamics, which helps candidates quickly move toward high-quality regions. As the iterations increase, $\beta \left( t \right)$ reduces, resulting in smaller and more stable movements, thereby enhancing fine-grained exploitation near convergence. The walrus weight is initialized with an upper bound of $\beta_{max}$ = 2 and a lower bound of $\beta_{min}$ = 0.2.

GOA weight $\emptyset \left( t \right)$ is computed as $\emptyset \left( t \right) = \emptyset_{\max } - \left( {\emptyset_{\max } - \emptyset_{\min } } \right) \times \frac{t}{T}$ This coefficient begins close to $\emptyset_{\max }$, ensuring that GOA-based grazing plays a dominant role in early exploitation by guiding candidates smoothly toward elite solutions. As the iterations increase, $\emptyset \left( t \right)$ gradually decreases, reducing the grazing pressure and allowing WO dynamics to dominate during later refinements. This balance ensures that exploitation transitions from smooth, elite-guided convergence to sharper, behavior-driven adjustment. The GOA weight is initialized with an upper limit of $\emptyset_{\max }$ = 1 and a lower limit of $\emptyset_{\min }$ = 0.2.

Elite jitter coefficient $\eta \left( t \right)\varepsilon^{t}$ is computed as ${ }\eta \left( t \right) = \eta_{0} \left( {1 - \frac{t}{T}} \right)$ this parameter regulates the strength of elite injection, where $\varepsilon^{{t{ }}}$ is sampled from the top ${{\% }}p$ of the population at each iteration. In the initial stages of the optimization,${ }\eta \left( t \right)$ is large, introducing a strong pull toward the elite pool and stabilizing the search trajectory. As the iterations increase, $\eta \left( t \right){ }$ decays to zero, reducing elite dominance and preventing premature convergence to suboptimal regions. This elite jitter guarantees that there is controlled randomness around elite candidates and diversity in the exploitation stage. The initial jitter parameter is given as $\eta_{0}$ = 5 × 10^−3^.

This adaptive hybrid formulation exchanges the static update rules of the original WO and GOA algorithms, enabling a context-sensitive exploitation process that balances convergence pressure with exploration robustness.

#### Stagnation detection and diversity preservation

To prevent premature convergence and loss of population diversity, the AIRE_WGO algorithm incorporates a stagnation detection and diversity restoration mechanism. A stagnation counter is employed to record the number of consecutive iterations during which the global best solution remains unchanged. When this counter exceeds a predefined threshold set to 20 iterations, the algorithm detects a stagnation condition, signaling that the population has lost its exploratory potential. In response, a fraction of the population (20% of the agents) is randomly reinitialized within the search boundaries.

The algorithm also normalizes the population diversity measure $div\_norm$ and uses it to modulate exploration-related parameters such as $\alpha_{t}$ and ${ }\beta_{t}$. High diversity triggers stronger exploratory movements, while low diversity encourages tighter exploitation around the elite regions. Consequently, AIRE_WGO maintains a self-regulating search dynamic, where exploration and exploitation are continuously balanced through both reinforcement feedback and diversity-based adaptation.

This dual mechanism of stagnation monitoring and diversity restoration plays a vital role in sustaining the algorithm’s robustness, ensuring its capability to escape local traps and maintain effective global search coverage until convergence.

### Proposed 2: dual-mode adaptive reinforced switching walrus–gazelle optimizer (DMARS_WGO)

The Dual-Mode Adaptive Reinforced Switching Walrus Gazelle Optimizer (DMARS_WGO) introduces a new generation of adaptive evolutionary intelligence that extends the AIRE_WGO framework toward higher autonomy, scalability, and decision stability. Unlike AIRE_WGO, which relies on a single Q learning paradigm to guide behavioral adaptation, DMARS_WGO introduces a dual-mode reinforcement mechanism that allows the algorithm to dynamically alternate between tabular Q learning and deep neural Q-value estimation (DQN) depending on the search landscape. This adaptive switching is guided by internal indicators, population diversity, improvement rate, and stagnation that enable DMARS_WGO to select the most suitable learning paradigm at each stage. Additionally, a context-aware blending coefficient ($\lambda_{t}$) smoothly detects the actions proposed by the Q learning and DQN agents, guiding the optimizer to operate through one of three coordinated movement strategies, which are GOA for exploratory diversification, WO for intensive exploitation around promising regions, and a mixed mode that adaptively balances both behaviors according to the search context. A cross-agent knowledge exchange further strengthens learning cooperation, where Q learning experiences enrich the DQN’s replay buffer, and distilled DQN knowledge improves the Q-table entries.

Compared to conventional metaheuristic algorithms and existing reinforcement-assisted optimizers, this dual-mode framework provides several distinctive advantages. First, by combining discrete tabular learning with neural function approximation, DMARS_WGO mitigates the limitations of single-policy reinforcement mechanisms, reducing instability caused by coarse state discretization or neural approximation bias. Second, the adaptive switching strategy prevents dominance bias toward either exploration or exploitation, a common limitation in static or heuristically scheduled hybrid models. Third, the smooth behavioral regulation enabled by $\lambda_{t}$ avoids abrupt phase transitions, enhancing convergence stability.

**Fig. 3:**
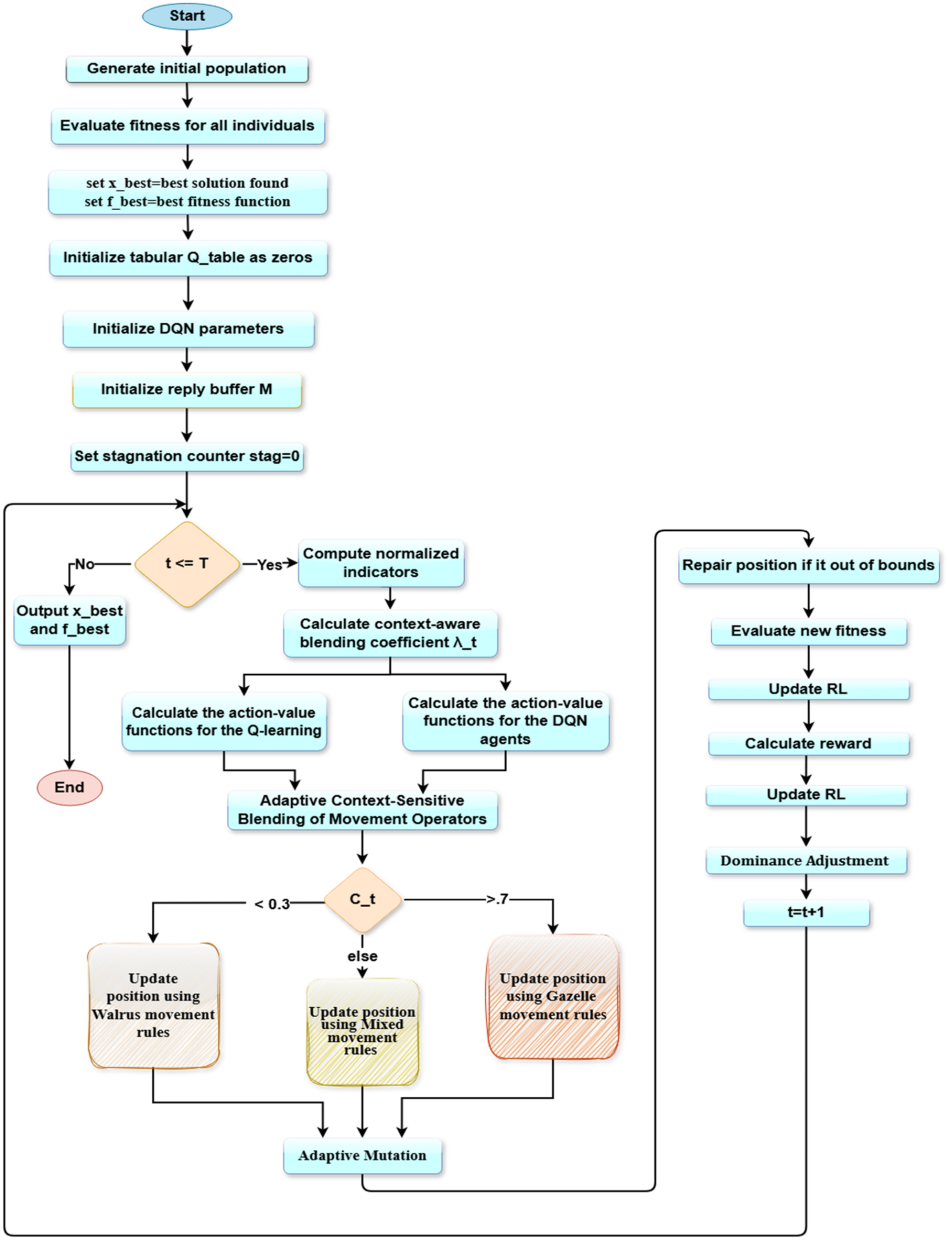
the flowchart of the proposed algorithm DMARS_WGO.

Through this cooperative interaction, DMARS_WGO demonstrates faster convergence, greater robustness, and more stable optimization behavior across complex, high-dimensional problem domains. As shown in Fig. 3, the complete flowchart begins with population initialization and fitness evaluation, followed by reinforcement-guided decision making, context-sensitive movement selection, and adaptive mutation. The pseudo code is described in Algorithm 4, and the details of the algorithm are illustrated in the following subsections.

AIRE_WGO is presented as a reinforcement-based of the classical WO-GOA hybrid which deploys a single Q-learning policy to control behavior. DMARS_WGO is an extension of this implementation to add a dual-mode cooperative reinforcement framework that combines both tabular Q-learning and DQN. Thus, AIRE_WGO is used as a mid-way reinforcement framework whereas DMARS_WGO is the complete dual-mode framework. This process of staged design can be used to validate enhancement based on reinforcing progressively.


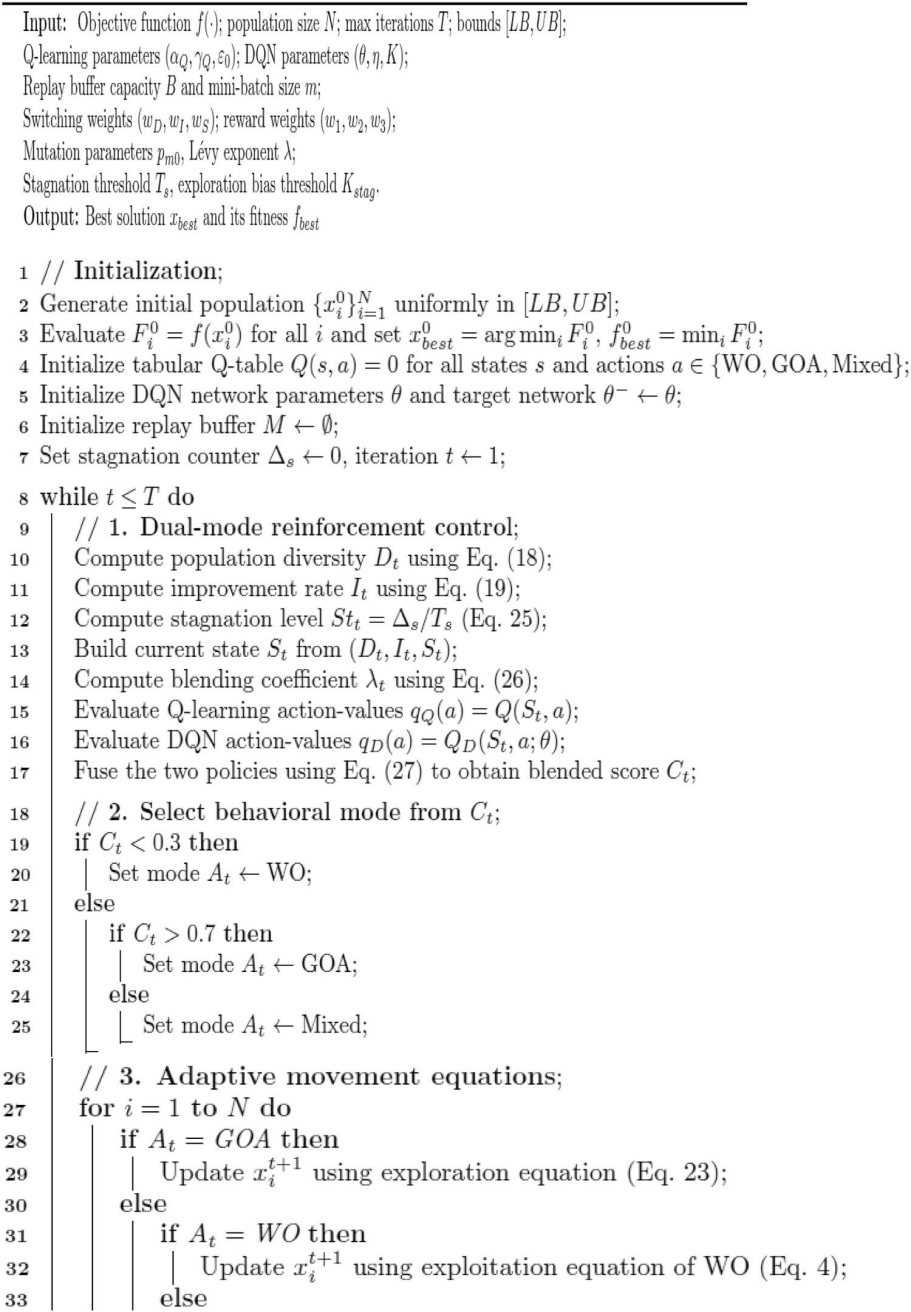


**Algorithm 4:**
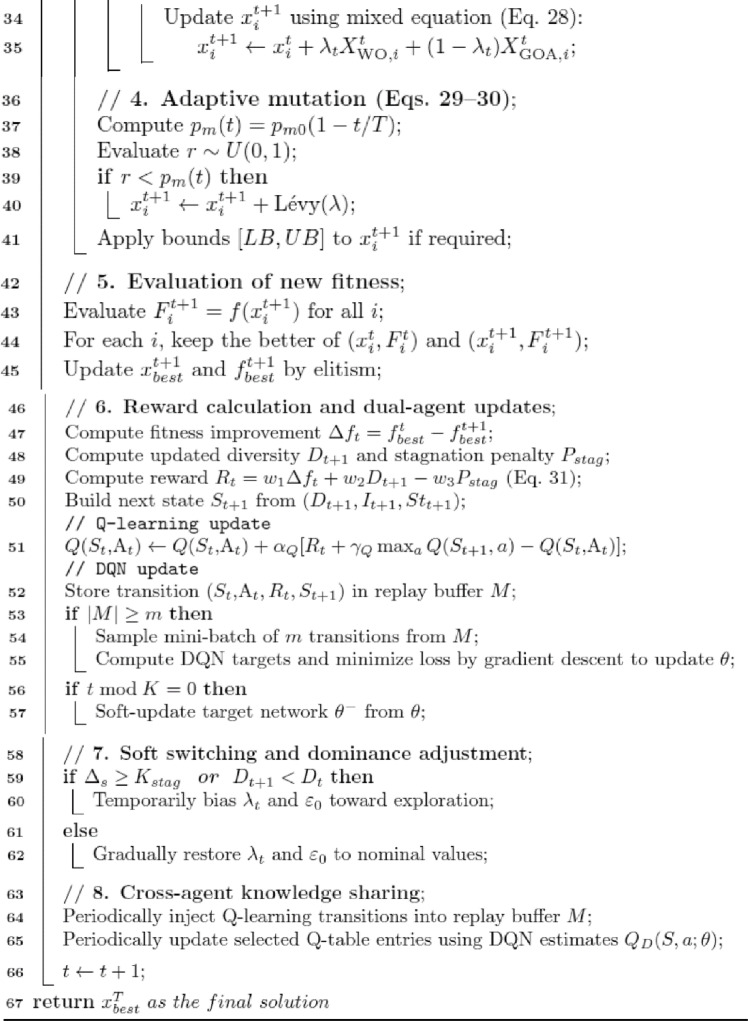
Pseudo-code of DMARS_DGO (Dual-Mode Adaptive Reinforced Switching Walrus–Gazelle Optimizer)

#### Dual mode reinforcement control

During each iteration, the algorithm evaluates its internal state $S_{t}$ using three feedback indicators, which are population diversity ($D_{t}$), improvement rate ($I_{t}$), and stagnation level ($St_{t}$). The diversity of populations $D_{t}$ shows how dispersed the individuals are around the population mean and is computed as provided in Eq. 18. The improvement rate $I_{t}$ quantifies the relative enhancement in the global best fitness between consecutive iterations and is computed as shown by Eq. 19.

The stagnation level ${\mathrm{St}}_{{\mathrm{t}}}$ tracks how long the optimization has progressed without improvement and is computed by Eq. 25:

25 $$ {\mathrm{St}}_{{\mathrm{t}}} = \frac{{\Delta_{{\mathrm{s}}} \left( {\mathrm{t}} \right)}}{{{\mathrm{T}}_{{\mathrm{s}}} }} $$

where $\Delta_{{\mathrm{s}}} ({\mathrm{t}}$) is the Consecutive stagnant iterations, $T_{s}$ is the stagnation threshold.

After calculating these indicators, the state $S_{t}$ is computed as [$D_{t} ,I_{t} ,St_{t}$] and DMARS_WGO combines them through an adaptive switching function to determine the blending coefficient ($\lambda_{t}$) that balances the influence of both reinforcement agents as in Eq. 26:

26 $$ {\uplambda }_{{\mathrm{t}}} = \frac{{{\mathrm{w}}_{{\mathrm{D}}} {\mathrm{D}}_{{\mathrm{t}}} + {\mathrm{w}}_{{\mathrm{I}}} {\mathrm{I}}_{{\mathrm{t}}} }}{{{\mathrm{w}}_{{\mathrm{D}}} {\mathrm{D}}_{{\mathrm{t}}} + {\mathrm{w}}_{{\mathrm{I}}} {\mathrm{I}}_{{\mathrm{t}}} + {\mathrm{w}}_{{\mathrm{S}}} {\mathrm{St}}_{{\mathrm{t}}} + {\upvarepsilon }}} $$

where ${\mathrm{w}}_{{\mathrm{D}}} ,{\text{ w}}_{{\mathrm{I}}} ,{\text{ w}}_{{\mathrm{S}}}$ are weighting coefficients for diversity, improvement, and stagnation, and $\varepsilon$ is a small constant that prevents division by zero.

The final movement ${\mathrm{C}}_{{\mathrm{t}}}$ is obtained using a context-aware fusion of the Q learning and DQN policies as in Eq. 27:

27 $$ {\mathrm{C}}_{{\mathrm{t}}} = {\uplambda }_{{\mathrm{t}}} .{\mathrm{A}}_{{\mathrm{t}}}^{{\mathrm{Q}}} + \left( {1 - {\uplambda }_{{\mathrm{t}}} } \right).{\mathrm{A}}_{{\mathrm{t}}}^{{\mathrm{D}}} $$

where $A_{t}^{Q}$ and $A_{t}^{D}$ are the actions that the two reinforcement learners suggest.

This adaptive blending chooses the final movement from three actions: GOA for exploration, WO for exploitation, and hybrid WO and GOA.

#### Adaptive movement equations

The reinforcement controller uses the adaptive coefficient $\lambda_{t}$ to identify the behavioral mode. These obtained decisions are converted into specific mathematical movement equations by the DMARS_WGO, which directs the population's progress. If ${\mathrm{C}}_{{\mathrm{t}}}$ is less than 0.3, the action $A_{t}$ is WO. If the ${\mathrm{C}}_{{\mathrm{t}}}$ is more than 0.7, the action $A_{t}$ is GOA. Otherwise, the action $A_{t}$ is a mix of WO and GOA. Every iteration updates the positions of all individuals using a particular movement equation that is chosen from the reinforcement agents. When GOA mode is selected, the algorithm performs global exploration to expand the search and preserve population diversity. The position of each individual is updated according to the formulation in Eq. 23, inspired by the exploration dynamics of the earlier proposed AIRE_WGO algorithm. When WO mode is selected, the optimizer switches to local exploitation, concentrating on fine-grained refining around promising regions. Its movement is expressed as in Eq. 4, inspired by the exploitation equation of the original WO. When the mixed mode is selected, the population movement is determined using the formulation as in Eq. 28:

28 $$ {\mathrm{X}}_{{\mathrm{i}}}^{{{\mathrm{t}} + 1}} = {\mathrm{X}}_{{\mathrm{i}}}^{{\mathrm{t}}} + {\uplambda }_{{\mathrm{t}}} {\mathrm{X}}_{{{\mathrm{WO}}}}^{{\mathrm{t}}} + \left( {1 - {\uplambda }_{{\mathrm{t}}} } \right){\mathrm{X}}_{{{\mathrm{GOA}}}}^{{\mathrm{t}}} $$

where ${\mathrm{X}}_{{{\mathrm{WO}}}}^{{\mathrm{t}}}$ corresponds to the formulations already described in Eq. 4 and ${\mathrm{X}}_{{{\mathrm{GOA}}}}^{{\mathrm{t}}}$ corresponds to the formulations already described in Eq. 14. By using this dynamic weighting method, DMARS_WGO consistently strikes a balance between its exploration and exploitation capabilities.

#### Adaptive mutation

DMARS_WGO uses a conditional adaptive mutation mechanism that is run after each movement update to avoid population stagnation and preserve sufficient diversity during the optimization phase. The mutation probability is defined as in Eq. 29:

29 $$ p_{m} \left( t \right) = p_{m0} \left( {1 - \frac{t}{T}} \right) $$

where $$p_{m0}$$ is the initial mutation probability. At each iteration, the algorithm first evaluates this probability and compared with a randomly generated number $$r \in \left[ {0,1} \right]$$. If $$p_{m} \left( t \right)$$ exceeds $$ r$$, a levy mutation is applied as in Eq. 30; Otherwise, the current individual remains unchanged.

30 $$ {\mathrm{X}}_{{\mathrm{i}}}^{{{\mathrm{t}} + 1}} = {\mathrm{X}}_{{\mathrm{i}}}^{{{\mathrm{t}} + 1}} + L\mathop e\limits^{\prime } vy\left( \lambda \right) $$

where $L\mathop e\limits^{\prime } vy\left( \lambda \right)$ represents a random step drawn from a Lévy distribution with exponent λ.

At the early iterations of the optimization, the value of $p_{m} \left( t \right)$ is relatively high, resulting in stronger and more frequent mutations that promote wide exploration and discovery of diverse promising regions. As iterations progress, $p_{m} \left( t \right)$ decreases gradually, reducing the mutation. This controlled decay narrows the search focus around high-quality areas, stabilizing the convergence behavior and refining the final candidate solutions. After this step, boundary repair is applied if it is required. Then the algorithm calculates the new fitness $f\left( {x_{t + 1}^{i} } \right)$ and updates $x_{best}$, $f_{best}$.

#### Reward calculation and dual-agent updates

At the end of each iteration, DMARS_WGO interprets the observed optimization progress as a reinforcement feedback signal, which drives the cooperative learning dynamics of its Q learning and DQN modules. The reward function is formulated as in Eq. 31:

31 $$ {\mathrm{R}}_{{\mathrm{t}}} = {\mathrm{w}}_{1} \Delta {\mathrm{f}}_{{\mathrm{t}}} + {\mathrm{w}}_{2} {\mathrm{D}}_{{{\mathrm{t}} + 1}} - {\mathrm{w}}_{3} {\mathrm{P}}_{{{\mathrm{stag}}}} $$

where $\Delta f_{t}$ represents the fitness improvement of the global best solution, $D_{t + 1}$ denotes the updated population diversity, $P_{stag}$ quantifies the stagnation penalty accumulated across successive non-improving iterations, and $w_{1}$, ${ }w_{2}$, ${ }w_{3}$ are the weighting coefficients that control the relative contribution of progress, diversity, and stability to the overall reinforcement response.

This reward signal is simultaneously propagated to the two learning modules. For the Q learning agent, the discrete Q-table is updated as in Eq. 18 in parallel, and the DQN module processes the same reward by storing the transition $\left( {S_{t} ,A_{t} ,R_{t} ,S_{t + 1} } \right)$ within its replay buffer. Once the replay buffer accumulates a sufficient number of transitions (∣M∣ ≥ m), where M is the number of transitions in the replay buffer and m is a batch size, the DQN initiates a training cycle. A random mini-batch of m transitions is sampled from the buffer to ensure uncorrelated updates and stable convergence. For each sampled experience, the target Q value is computed. Then the loss function is calculated and minimized through gradient descent to update the parameters $\theta$ of the primary network. Through this iterative replay-based learning process, the DQN gradually refines its approximation of the true action value function and improves its decision across successive iterations.

In flat fitness areas with low density of the improvement signals, the reinforcement learning layer of DMARS_WGO is still fed with the signal of diversity variation and stagnation detection. The lack of improvement enhances the stagnation indicators thus skewing the switching mechanism to favor exploration-dominant behavior. Together with ε-greedy exploration and adaptive mutation, the design discourages the stalling of learning process by rare reward signals and aids in escaping plateau regions.

#### Soft switching and dominance adjustment

The DMARS-WGO also incorporates a Soft Switching and Dominance Adjustment mechanism that constantly checks the search dynamics to ensure stagnation does not occur, and the adaptive balance between exploration and exploitation is kept. On the one hand, the algorithm monitors the diversity of the population $D_{t}$ and the development of the global best fitness at every iteration. In cases where the global optimum solution fails to improve, the stagnation counter $St_{t}$ is increased; in other cases, it is set to zero. The time the search has been stuck in a poor region is countered by this counter. When the stagnation counter goes above a specified threshold $K_{stag} { }$ or when the diversity of the population decreases $D_{t + 1,} { }$ the algorithm takes this as an early warning of an imminent stagnation. In a bid to come out, DMARS-WGO temporarily shapes its reinforcement control towards exploration. It can be done by raising the exploration probability ε of the Q-learning agent or changing the coefficient of blending ${\uplambda }_{{\mathrm{t}}}$ to highlight the exploratory movements (preferring the GOA-based operator). This temporary supremacy of exploration allows the population to overcome the local optima and get back to diversity. As soon as a measurable increase in the fitness is observed or the diversity returns, the algorithm slowly drives ${\uplambda }_{{\mathrm{t}}}$ and ε back to their nominal equilibrium value. This progressive recovery is a good transition that guarantees a smooth behavioral transition eliminating oscillations and providing constant convergence performance.

DMARS_WGO incorporates tabular Q-learning and DQN in a collaborative decision layer. The dominance of learners is not as a hard switch, but instead, a context-sensitive blending coefficient ${\uplambda }_{{\mathrm{t}}}$ ∈ [0,1] offers an interpolative transition between two action recommendations. Generally, tabular Q-learning is more efficient at the initial stages of search and highly exploratory states, where discrete state feedback and little experience prefer rapid updates of local values. As the replay buffer fills out enough and the optimization enters less unstructured exploitation phases, the DQN policy will be more informative as a result of its generalization ability. The dominance regulation is also regulated with the diversity and stagnation indicators to avoid oscillatory switching via a threshold hysteresis (i.e. switching is only done when indicators pass margins and not at the boundary values). Furthermore, there is a small persistence window such that, when a predominant learner has been chosen, it will continue to be maintained a minimum number of times before being re- evaluation. These mechanisms prevent the recurrent switching of learners and maintain constant transitions throughout the search.

#### Cross-agent knowledge sharing

DMARS_WGO implements a cross-agent knowledge sharing scheme that allows the Q learning and DQN to share information collaboratively in order to ensure that the two reinforcement layers are consistent. Individual experiences obtained in the course of the Q-learning updates are repeatedly added to the replay buffer of the DQN, which increases its exposure to a variety of state-action-reward transitions. This move broadens the range of training of the DQN, increasing its generalizability to dynamic optimization scenarios. On the other hand, once a specified synchronization period, the DQN transmits its learned $Q\left( {S,a;\theta } \right)$ approximations to modify the tabular Q values. This biased process of distillation makes the Q learning policy focus on more globally consistent assessments based on the neural model.

#### DQN architecture and training configuration

DQN module in DMARS_WGO is a completely connected feedforward neural network with two hidden layers consisting of 64 neurons each, where the activation functions are ReLU. The discrete action space is associated with the output layer which employs linear activation. This network is trained by the Adam optimizer with the learning rate of 0.001. It has a replay buffer of 10,000 and mini-batches of 32 are sampled to train. The parameters of network are updated after every iteration. Rewards are normalized to ensure restricted learning indications. In the ε-greedy exploration approach, the exploration strategy takes the deterministic decay policy between ε₀ to ε min throughout the iterations. Experiments were done using a fixed DQN hyperparameter in order to have reproducibility.

### Computational complexity analysis

Let N be the population size, D the problem dimension, and T the maximum number of iterations. In each iteration, DMARS_WGO updates the position of all agents and optimizes the objective function. The update of the population (GOA/WO/Mixed) requires basic vector operations with complexity *O*(*N* $\times$ *D*)*.* The cost of fitness evaluation is *O*(*N* $\times$ *Cf*(*D*))*,* where *Cf*(*D*) denotes the computational cost of evaluating the objective function at dimension D.

In the reinforcement-learning part, they are (i) tabular Q-learning updates and (ii) DQN inference/training. The Q-learning update is *O*(*1*) per iteration since it updates a single state–action entry. In the case of the DQN module, forward inference is *O*(*P*)*,* and training with a mini-batch of size mmm is *O*(*mP*)*,* where PPP denotes the number of network parameters (a constant for a fixed architecture). Therefore, the RL overhead per iteration is *O*(*1* + *mP*)*,* which is independent of *D* for a fixed state representation size.

This means that the overall complexity of DMARS WGO per iteration can be stated as:

$$ O(N \times D + N \times Cf\left( D \right) + mP). $$

Since P and m are constants in our implementation and *N* $\times$ *Cf*(*D*) generally in the first term in benchmark and engineering problems, the total time complexity in T iterations is:

$$ O(T \times (N \times D + N \times Cf(D))) \approx O(T \times N \times D)\,\,\,\,\,\,(when\,Cf(D) = O(D)) $$

That means that DMARS_WGO has the same asymptotic order as standard population-based metaheuristics, in the typical objective evaluation cost, with the dual-mode reinforcement mechanism providing a limited additional overhead. To make use of the opportunity, DMARS_WGO presents a DQN training loop into the metaheuristic cycles, which provides an additional computational cost in contrast to AIRE_WGO and classical metaheuristics. This overhead is, however, limited since the DQN architecture and mini-batch size are constant, therefore, the cost of an update per iteration is approximately constant. In standard benchmark and engineering design problems, objective function evaluation on the population is often of primary cost, particularly when the functional in question is computationally expensive. Hence, although DMARS_WGO requires additional runtime due to DQN optimization, the overhead remains moderate under the adopted settings and does not change the overall scalability behavior characterized by (*TND*).

### Scalability discussion

Theoretically, DMARS_WGO has the same computational complexity of O(T × N × D), as the traditional population-based metaheuristics. The proposed dual-mode reinforcement learning only creates a small, constant overhead and will not change the asymptotic scaling of the algorithm.

In an empirical perspective, the CEC2017 and CEC2022 benchmark suites consist of hybrid and composition functions, which model extensive and high complexity search landscapes. These categories of functions provide evidence of good adaptability and robustness of DMARS WGO with the escalating complexity of the problems because its ranking performance has been constant throughout the range of these classes. All these findings are indicative that DMARS WGO is able to maintain scalability at theoretical as well as experimental levels.

## Experimental results and analysis

This section presents the experimental results of applying AIRE_WGO algorithm and DMARS_WGO algorithm on various benchmarks and testing their performance against other algorithms. To ensure that all algorithms are given a fair chance, all algorithms have been applied for 51 separate runs and 500 iterations. The initial populations are the same for all algorithms. This paper has used CEC 2017 benchmark functions, CEC 2022 benchmark functions, and 6 engineering problems to compare all algorithms. All experiments have been simulated using a computer with Dell core i7 processor and 16 GB RAM and by using MATLAB R2020b. Table 1 gives the parameters of the used algorithms, which are taken from their original references.

**Table 1:** Initial parameter settings of the algorithms used for comparison.

Algorithm	Parameters
WO	*a* = 2 × (1 − *t*/*tmax*) β = 1 − *t*/*tmax*
GOA	PSRs = 0.34 S = 0.88
GJO	–
OOA	–
PDO	$\rho = 0.1$, $\varepsilon = 2.2204 \times 10^{ - 16}$, and ${\Delta } = 0.005$
POA	$R = 0.2$
RSO	$R \in \left[ {1, 5} \right]$ and $C \in \left[ {0, 2} \right]$
CAO	–
RFO	α = 0.1, β = 0.5
SAO	$olf = 0.75$ and $SL = 0.9$
MShOA	*pm* = 0.1
AL-SHADE	$H = 6$, $p = 0.11$, and $e = 0.5$

### Simulation results of CEC 2017 benchmark functions

The proposed DMARS_WGO is evaluated against AIRE_WGO, WO, and GOA and also against nine state-of-the-art algorithms using the 29 CEC 2017 benchmark functions (F2 is a dummy), which are divided to two unimodal functions (F1–F3), seven simple multimodal functions (F4–F10), ten hybrid functions (F11–F20), and ten composition functions (F21–F30)^77^. Table 2 demonstrates the operation of these functions.

**Table 2:** Details of CEC-2017 benchmark functions.

No	Type	Function name	Optimal
F1	Unimodal	Rotated and shifted Bent Cigar	100
F3		Rotated and shifted Zakharov	300
F4	Multimodal	Rotated and shifted Rosenbrock’s	400
F5		Rotated and shifted Rastrigin’s	500
F6		Rotated and shifted Expanded Schaffer’s	600
F7		Rotated and shifted Lunacek Bi_Rastrigin	700
F8		Rotated and shifted non-continuous Rastrigin’s	800
F9		Rotated and shifted Levy	900
F10		Rotated and shifted Schwefel’s	1000
F11	Hybrid	Hybrid_F1	1100
F12		Hybrid_F2	1200
F13		Hybrid_F3	1300
F14		Hybrid_F4	1400
F15		Hybrid_F5	1500
F16		Hybrid_F6	1600
F17		Hybrid_F7	1700
F18		Hybrid_F8	1800
F19		Hybrid_F9	1900
F20		Hybrid_F10	2000
F21	Comparison	Composition_F1	2100
F22		Composition_F2	2200
F23		Composition_F3	2300
F24		Composition_F4	2400
F25		Composition_F5	2500
F26		Composition_F6	2600
F27		Composition_F7	2700
F28		Composition_F8	2800
F29		Composition_F9	2900
F30		Composition_F10	3000

#### DMARS_WGO vs AIRE_WGO, GOA, and WO

**Fig. 4:**
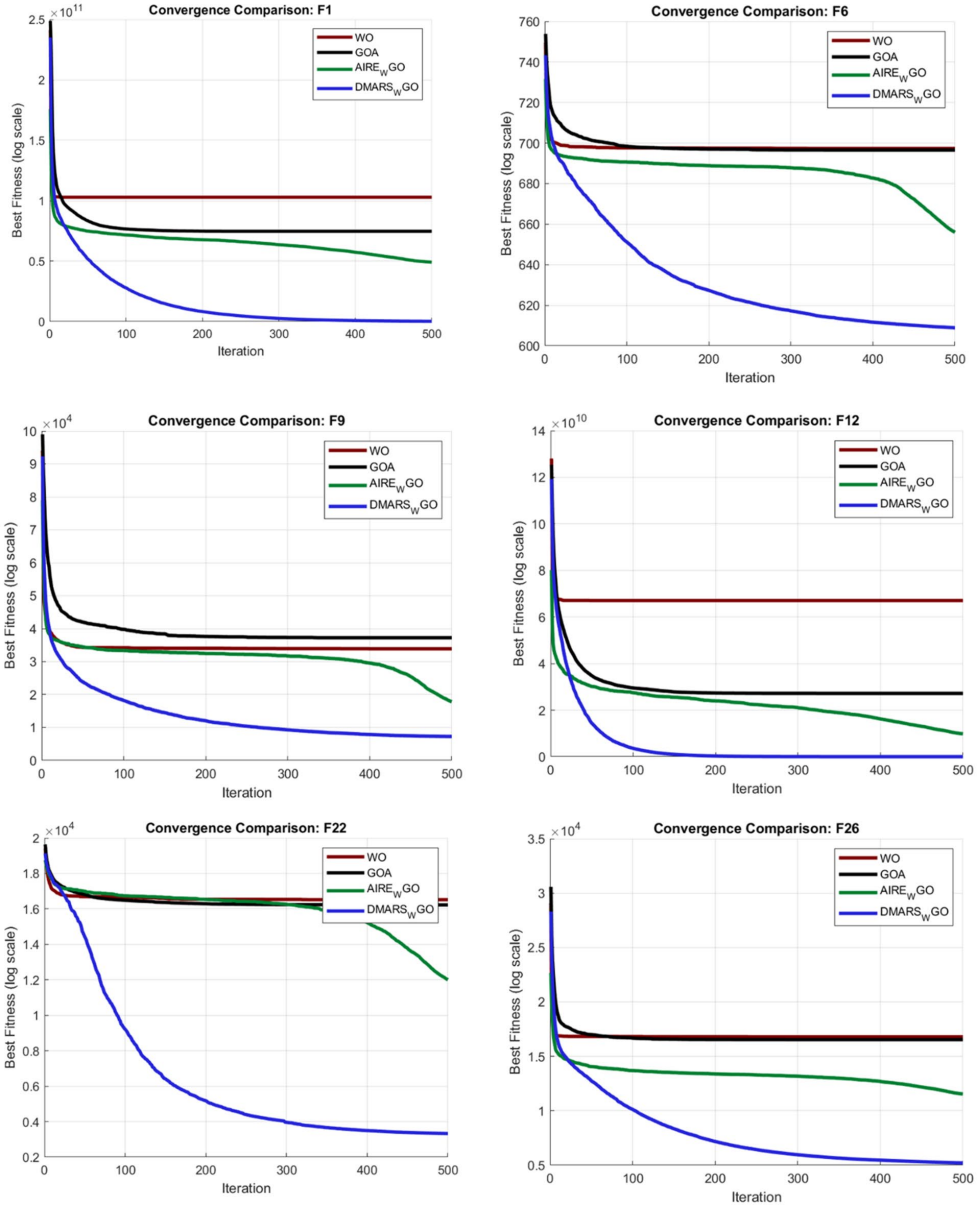
Samples of Convergence plots of DMARS_WGO, AIRE_WGO, GOA and WO on CEC 2017.

**Fig. 5:**
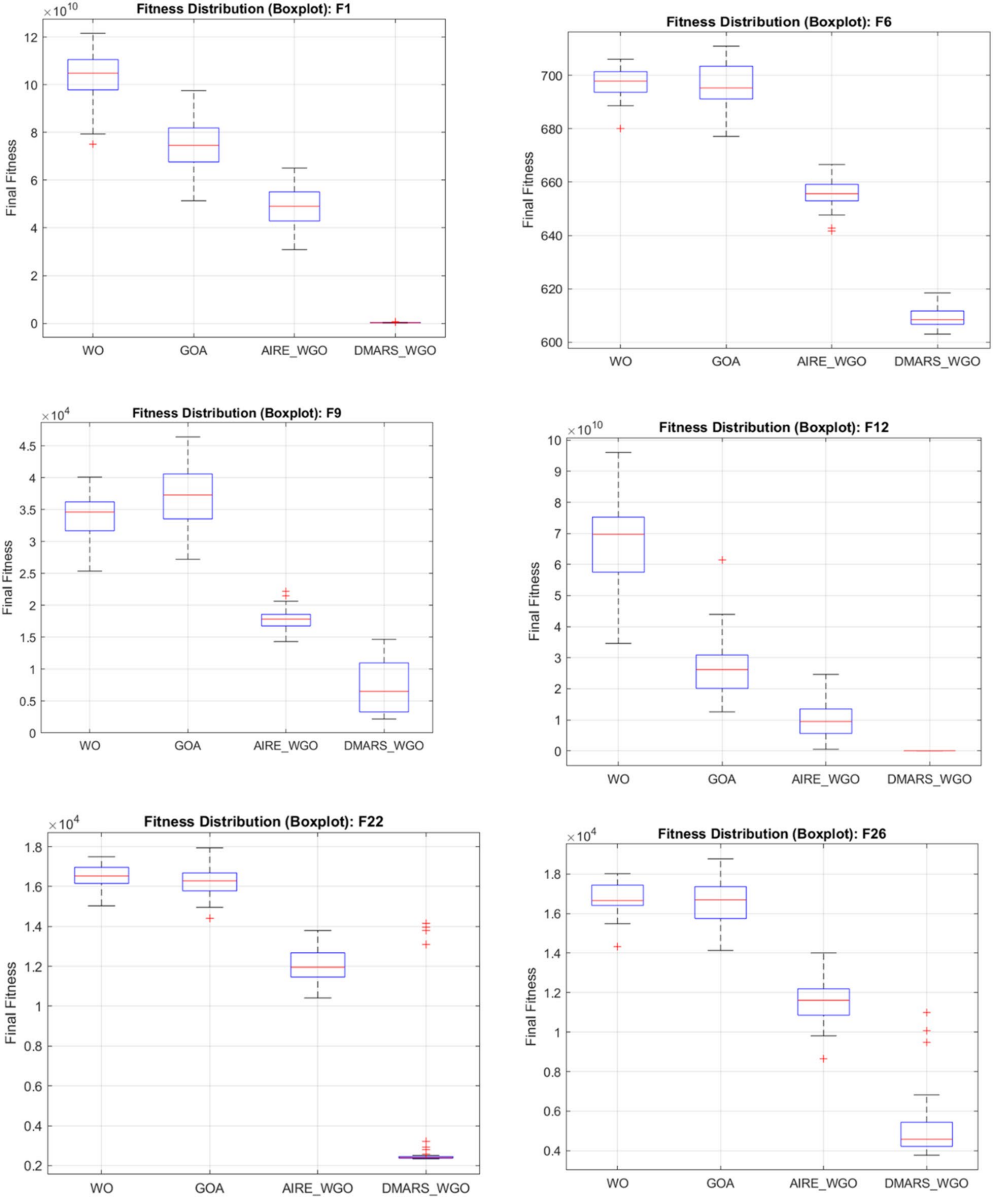
Samples of boxplot of DMARS_WGO, AIRE_WGO, GOA and WO on CEC 2017.

The proposed DMARS_WGO algorithm is compared to its predecessor AIRE_WGO and the baseline bio-inspired algorithms GOA and WO in order to evaluate its effectiveness and adaptability. It has proven effective not only for low dimensional but also for high dimensional optimization problems. For each metric, we record and compare the best, Mean, STD, and Rank of the achieved results. The outcomes of DMARS_WGO against AIRE_WGO, GOA, and WO to F1-F30 issues are shown in Table 3 where the best values for each benchmark are shown in bold. DMARS_WGO accomplishes the best mean values for all functions (except F3, F10, and F20). As compared to AIRE_ WGO, GOA, and WO, DMARS_WGO achieves vastly improved results attesting to the optimizer’s higher performance. In this study, the Wilcoxon signed rank test has been employed to ensure the reliability of the comparison results obtained on the CEC 2017 benchmark. The test evaluates whether the observed improvements of DMARS_WGO over competing algorithms are statistically significant rather than arising from random variations. Table 4 shows that DMARS_WGO achieves statistically significant improvements over other compared algorithms, with *p*-values < 0.05 for benchmark functions demonstrating the effectiveness of its dual-mode learning structure. Despite the Wilcoxon tests of rank being conducted separately in benchmark functions, Friedman mean rank analysis offers most conclusions because it allows a comparison of multiple problems on a global scale. The point of agreement between rank-based testing and pairwise significance testing makes it less likely that Type-I statistical inflation will cause conclusions to be drawn out of single-point inflation. Convergence curves shown in Fig. 4 allow us to visually compare the convergence rates of DMARS_WGO and other algorithms. These diagrams depict typical objective values achieved by algorithms at various stages of their iterative process. Convergence plots depict iterations on the horizontal axis and objective function values on the vertical axis. The plots make it clear that the suggested DMARS_WGO has a faster convergence rate than the AIRE_WGO, GOA, and WO. The convergence curves show that DMARS_WGO has a greater search diversity at the initial iterations, which allows it to explore the search space in a wider way. The intermediate stage involves the dynamic adjustment of behavioral dominance by the reinforcement-driven switching mechanism depending on the indication of improvement and stagnation, which avoids premature convergence. In the last step, the blending coefficient slowly introduces the search to exploitation-oriented movements, leading to steady smooth convergence to the near-optimal areas. DMARS_WGO converges more quickly to baseline algorithms and is more robust to multimodal and hybrid functions (which are more susceptible to premature stagnation) than baseline algorithms. The lack of oscillatory convergence patterns is also an indication of stable learning-based adaptation. Boxplot comparison of DMARS_WGO, AIRE_WGO, GOA, and WO across benchmark functions is shown in Fig. 5. The figure clearly shows that DMARS_WGO achieves the most stable and consistent performance, with narrower interquartile ranges and lower median fitness values than the other algorithms.

**Table 3:** Comparison of results of DMARS_WGO, AIRE_WGO, GOA and WO on CEC 2017.

F	Algorithm	WO	GOA	AIRE_WGO	DMARS_WGO
F1	Best	74,985,151,451	51,349,180,700	30,883,383,805	99,365,265.62
	Mean	1.02909E + 11	74,735,434,572	49,154,555,910	**257,742,421.6**
	Std	10,350,182,133	9,872,845,809	8,537,595,937	101,985,536.4
	Rank	4	3	2	**1**
F3	Best	149,001.2124	167,372.7762	93,646.24117	123,087.7106
	Mean	188,766.1608	285,669.1394	**127,712.5665**	150,367.4736
	Std	15,604.82517	104,489.2229	13,753.29871	12,215.22119
	Rank	3	4	**1**	2
F4	Best	23,081.51396	11,431.39001	4331.08965	584.2432414
	Mean	33,215.98073	20,390.33666	7988.393753	**702.4862444**
	Std	4248.24894	5276.936747	2181.390022	61.61492807
	Rank	4	3	2	**1**
F5	Best	1107.86075	1089.604125	861.6260989	728.5294561
	Mean	1164.075045	1172.79609	917.8338016	**893.2501283**
	Std	29.81196384	45.58846088	33.61924269	62.495179
	Rank	3	4	2	**1**
F6	Best	680.0505046	677.1150722	641.7226976	603.0035281
	Mean	697.3649729	696.5677111	655.9916488	**608.8933288**
	Std	5.345425796	8.212396657	5.400084601	3.47055345
	Rank	4	3	2	**1**
F7	Best	1752.686775	1783.007396	1322.737224	1118.593692
	Mean	1982.677327	2013.157456	1515.18343	**1195.83094**
	Std	77.52582768	72.46142785	80.19393743	43.8852907
	Rank	3	4	2	**1**
F8	Best	1329.492153	1359.359224	1141.793442	1047.436848
	Mean	1461.691838	1474.555576	1238.049762	**1208.372843**
	Std	41.25037721	45.18300887	39.91118647	54.20574872
	Rank	3	4	2	**1**
F9	Best	25,355.74126	27,211.00666	14,314.38169	2175.223233
	Mean	33,879.04262	37,245.71549	17,837.1911	**7276.927637**
	Std	3667.571977	5104.243874	1769.855805	4018.700806
	Rank	3	4	2	**1**
F10	Best	13,805.68669	12,917.72377	6839.334227	10,491.8665
	Mean	14,887.27909	14,507.6171	**9117.537207**	11,784.4832
	Std	495.7416852	830.9650771	907.0283132	493.6053309
	Rank	4	3	**1**	2
F11	Best	18,469.73849	8678.52538	4366.05749	1472.084594
	Mean	24,850.43601	17,382.86034	8058.903759	**1685.074765**
	Std	2194.006403	4282.096879	2106.204951	103.4145324
	Rank	4	3	2	**1**
F12	Best	34,577,862,100	12,581,872,967	536,681,112.5	1,971,378.113
	Mean	67,088,168,389	27,200,100,306	9,805,054,959	**8,676,347.615**
	Std	13,144,708,693	9,434,850,731	5,705,909,656	4,102,909.476
	Rank	4	3	2	**1**
F13	Best	18,843,608,159	1,198,922,065	23,205,898.6	3633.037906
	Mean	38,310,244,300	6,895,446,301	1,147,048,521	**7130.047409**
	Std	11,100,870,977	4,828,179,011	2,288,642,755	3323.418485
	Rank	4	3	2	**1**
F14	Best	6,350,292.734	829,087.2609	226,331.2985	31,409.54403
	Mean	59,836,038.89	13,788,824.86	2,682,923.348	**165,328.5013**
	Std	39,382,754.66	11,200,500.22	2,342,625.964	82,604.62022
	Rank	4	3	2	**1**
F15	Best	1,411,179,547	139,049,979.2	1,646,795.202	6325.671863
	Mean	6,059,795,033	1,050,414,472	250,826,929.6	**15,132.61082**
	Std	2,636,008,100	825,186,165.8	363,995,839.3	3504.984666
	Rank	4	3	2	**1**
F16	Best	6486.816596	5796.328171	2972.021206	2513.289588
	Mean	8475.923903	7753.050703	3823.314027	**3723.061272**
	Std	1026.073104	1391.171236	439.5829094	535.1913929
	Rank	4	3	2	**1**
F17	Best	4579.604303	3939.336041	2665.969302	2344.473335
	Mean	6616.136816	6322.55956	3332.330906	**3098.138003**
	Std	2610.975974	2865.318036	324.3468068	250.3262596
	Rank	4	3	2	**1**
F18	Best	30,371,447.62	7,627,549.045	784,394.7435	766,856.0518
	Mean	117,127,641.7	91,516,762.17	11,291,869.13	**1,701,892.883**
	Std	54,292,192.32	59,278,194.9	12,177,922.88	573,148.6437
	Rank	4	3	2	**1**
F19	Best	253,667,290.2	44,477,340.57	210,573.4156	9914.609965
	Mean	2,323,978,983	486,653,740.8	21,723,560.2	**18,887.32514**
	Std	1,116,531,262	332,639,804	55,424,520.16	4652.21945
	Rank	4	3	2	**1**
F20	Best	3329.696825	3596.78833	2701.308776	2963.004631
	Mean	4027.803639	4201.08897	**3210.720201**	3249.690182
	Std	234.9533789	282.6782845	267.4542774	130.7617144
	Rank	3	4	**1**	2
F21	Best	2974.017928	2955.30341	2633.014859	2434.850881
	Mean	3173.158915	3134.651141	2742.876141	**2626.109015**
	Std	62.54788582	88.8414865	48.66267856	68.11697545
	Rank	4	3	2	**1**
F22	Best	15,032.26327	14,402.34216	10,412.38612	2346.149372
	Mean	16,520.23296	16,225.17072	12,005.86972	**3328.227288**
	Std	554.3183454	718.4604943	812.6132739	3077.636718
	Rank	4	3	2	**1**
F23	Best	4000.189157	3687.935189	3183.703571	2865.171908
	Mean	4421.55513	4077.989586	3429.06813	**3074.347795**
	Std	153.45547	202.3774218	90.94673814	57.5310963
	Rank	4	3	2	**1**
F24	Best	4213.827741	3778.447566	3718.895019	3086.242285
	Mean	4616.680163	4250.202391	3888.090073	**3242.39787**
	Std	209.1620033	188.0453436	100.8259509	52.96804956
	Rank	4	3	2	**1**
F25	Best	11,629.6244	7839.051663	4712.285332	3156.601692
	Mean	14,399.85673	10,913.818	6505.913054	**3253.861884**
	Std	1169.373569	1562.771469	926.3894182	38.96505136
	Rank	4	3	2	**1**
F26	Best	14,335.59242	14,127.55201	8638.46511	3774.611372
	Mean	16,789.43097	16,532.94762	11,533.64172	**5203.791562**
	Std	728.2449979	1178.460968	979.6975863	1719.214384
	Rank	4	3	2	**1**
F27	Best	5088.678634	4344.387411	3985.406977	3287.074604
	Mean	6464.299387	5659.527222	4439.151501	**3404.916578**
	Std	539.6868335	771.6802132	202.3880941	54.25113468
	Rank	4	3	2	**1**
F28	Best	10,271.10327	7871.507429	5220.849992	3558.23316
	Mean	12,562.86141	9781.936597	6710.442973	**3682.907757**
	Std	1031.240105	1218.996608	672.9112915	70.06520688
	Rank	4	3	2	**1**
F29	Best	11,383.70994	8856.462153	4380.639785	3899.566114
	Mean	44,670.25031	16,649.87498	5764.451791	**4675.414345**
	Std	32,589.09695	7007.581657	632.094065	353.1795223
	Rank	4	3	2	**1**
F30	Best	1,081,855,499	239,867,707.6	41,107,369.56	844,348.4041
	Mean	5,231,959,823	1,438,855,395	190,971,063.6	**1,130,620.264**
	Std	2,054,977,070	952,937,874.4	146,591,406.4	147,180.9514
	Rank	4	3	2	**1**
Average		3.793103448	3.206896552	1.896551724	**1.103448276**
Total Rank		4	3	2	**1**

**Table 4:** The Wilcoxon rank-sum test (p-value) for DMARS_WGO Vs AIRE_WGO, GOA and WO for CEC 2017.

DMARS vs	WO	GOA	AIRE_WGO
F1	3.30368E − 18***	3.30368E − 18***	3.30368E − 18***
F3	7.07298E − 17***	4.70263E − 18***	8.6974E − 12***
F4	3.30368E − 18***	3.30368E − 18***	3.30368E − 18***
F5	3.30368E − 18***	3.30368E − 18***	0.302692347^ ns^
F6	3.30368E − 18***	3.30368E − 18***	3.30368E − 18***
F7	3.30368E − 18***	3.30368E − 18***	3.30368E − 18***
F8	3.30368E − 18***	3.30368E − 18***	0.017190684*
F9	3.30368E − 18***	3.30368E − 18***	0.017190684*
F10	3.30368E − 18***	3.30368E − 18***	6.30374E − 18***
F11	3.30368E − 18***	3.30368E − 18***	3.30368E − 18***
F12	3.30368E − 18***	3.30368E − 18***	3.30368E − 18***
F13	3.30368E − 18***	3.30368E − 18***	3.30368E − 18***
F14	3.30368E − 18***	3.30368E − 18***	6.68332E − 18***
F15	3.30368E − 18***	3.30368E − 18***	3.30368E − 18***
F16	3.30368E − 18***	3.30368E − 18***	0.4454821^ ns^
F17	3.30368E − 18***	3.30368E − 18***	0.000626272***
F18	3.30368E − 18***	3.30368E − 18***	4.07863E − 10***
F19	3.30368E − 18***	3.30368E − 18***	3.30368E − 18***
F20	1.26782E − 17***	3.30368E − 18***	0.486402801^ ns^
F21	3.30368E − 18***	3.30368E − 18***	6.01196E − 15***
F22	3.30368E − 18***	3.30368E − 18***	1.63897E − 13***
F23	3.30368E − 18***	3.30368E − 18***	3.30368E − 18***
F24	3.30368E − 18***	3.30368E − 18***	3.30368E − 18***
F25	3.30368E − 18***	3.30368E − 18***	3.30368E − 18
F26	3.30368E − 18***	3.30368E − 18***	2.25853E − 17***
F27	3.30368E − 18***	3.30368E − 18***	3.30368E − 18***
F28	3.30368E − 18***	3.30368E − 18***	3.30368E − 18***
F29	3.30368E − 18***	3.30368E − 18***	7.83417E − 15***
F30	3.30368E − 18***	3.30368E − 18***	3.30368E − 18***

*denotes statistical significance at *p* < 0.05, while multiple asterisks indicate stronger significance levels.

#### DMARS_WGO vs other algorithms

In this part, we compare the proposed DMARS_WGO to various optimization algorithms, namely GJO^26^, OOA^27^, POA^28^, RSO^55^, SAO^56^, CAO^57^, RFO^58^, MShOA^29^, and ALSHADE^30^. All of algorithms selected for comparison are fiercely competitive and have proven their value in problem solving. The outcomes are compared along the 30th dimension and from F1 to F30 with a fixed dimension. Table 5 shows the obtained experimental results for benchmark function. The table shows the best, Mean, STD, and Rank of the achieved results. DMARS_WGO achieves the best mean values for all functions except (F3 and F10). As Table 5 shows, the total rank for DMARS_WGO is 1.

**Table 5:** Comparison of results of DMARS_WGO vs other algorithms on CEC 2017.

F	Algo	GJO	OOA	POA	RSO	SAO	CAO	RFO	MShOA	ALSHADE	DMARS_WGO
F1	Best	4,825,115,190	28,573,515,333	5,041,094,346	34,625,387,319	28,745,730,600	1,146,560,917	16,607,719,429	22,951,562,565	19,346,098,272	116,023.1563
	Mean	12,503,900,535	54,933,339,606	16,999,640,238	44,385,226,192	68,947,565,221	6,693,790,058	45,012,072,708	50,378,678,856	30,330,451,156	**631,394.8094**
	Std	4,400,950,607	8,747,338,933	5,825,251,905	5,207,587,139	9,811,927,398	4,001,020,930	10,669,377,767	13,912,719,399	6,003,897,224	452,436.7703
	Rank	3	9	4	6	10	2	7	8	5	**1**
F3	Best	37,949.45534	69,267.72428	33,070.98118	57,106.29016	72,195.63307	47,770.75312	53,618.79766	76,068.90476	90,218.68922	45,114.43615
	Mean	62,689.87819	91,421.17038	**46,095.09553**	85,524.37241	116,174.982	65,166.51469	90,031.97945	89,813.56113	145,048.9528	59,921.59372
	Std	9636.96354	8639.667242	8022.63988	22,905.69731	22,497.93879	7873.077526	18,945.6098	7237.200717	39,334.36494	5131.575482
	Rank	3	8	**1**	5	9	4	7	6	10	2
F4	Best	647.0933867	9218.514331	712.6707791	8760.835058	6905.10482	542.289305	4791.744487	5663.054815	1364.338492	481.1815918
	Mean	1525.234234	14,695.11419	2973.835868	17,946.91893	18,956.74466	1264.13609	11,422.17345	17,156.50303	5725.447469	**516.1514443**
	Std	972.2651022	2996.100372	1782.633309	3315.179185	3809.172503	808.6495204	3618.764503	6459.962139	4017.930313	13.09954493
	Rank	3	7	4	9	10	2	6	8	5	**1**
F5	Best	634.1187236	843.5501011	680.7251287	865.9163276	874.0271221	667.7986276	761.7041525	849.1835189	763.9996249	637.5141507
	Mean	726.1620263	914.2842611	789.8372187	916.3300084	984.6526929	778.8952225	864.6374675	908.2851729	835.0978204	**694.1248981**
	Std	50.36540928	34.96094135	34.47638242	23.8701247	33.76760236	45.77727885	45.44702223	38.43307886	28.88814736	21.37079411
	Rank	2	8	4	9	10	3	6	7	5	**1**
F6	Best	620.7328476	660.6622901	646.6322895	681.9220893	674.1700548	652.9201712	646.7396767	665.7935962	658.4698154	600.5430819
	Mean	638.6797298	686.4740956	664.140514	695.8560482	696.704597	666.0775593	672.2131462	687.1537561	675.5347411	**603.0791512**
	Std	10.35254293	8.08907819	6.807454323	7.735953751	7.950084228	6.152716755	13.11208082	9.772231938	10.20420233	2.620772809
	Rank	2	7	3	9	10	4	5	8	6	**1**
F7	Best	927.8094111	1270.878928	1045.278378	1270.178748	1410.491227	1034.122016	1169.191788	1245.823491	1135.212454	891.7169559
	Mean	1060.663168	1425.899235	1284.561098	1362.011794	1548.676186	1257.893742	1455.699297	1439.499714	1270.920483	**936.4081885**
	Std	55.22183036	54.59472534	69.71747916	53.16858494	57.48102997	64.2444425	151.7058602	64.06667794	55.37879354	28.89639175
	Rank	2	7	5	6	10	3	9	8	4	**1**
F8	Best	929.6387346	1091.125986	950.1013759	1062.091389	1141.71236	939.1448425	994.4305271	1060.947641	1027.275268	886.0371712
	Mean	975.4971769	1139.862991	996.8776838	1120.408876	1199.487281	999.308509	1092.697249	1139.355233	1085.704685	**967.3872744**
	Std	31.00786939	25.58290752	24.07303999	28.27532112	27.29507704	23.31894929	42.62433659	31.42441537	24.14685589	28.00639709
	Rank	2	9	3	7	10	4	6	8	5	**1**
F9	Best	2540.522314	7620.560934	3933.117633	7850.961893	11,186.84229	4466.840163	4743.568794	8020.033777	5906.417782	912.2524345
	Mean	5587.364144	10,584.62373	6031.433389	10,882.44113	14,934.76413	6050.293003	8838.920579	11,140.05871	9219.130363	**1869.253523**
	Std	1816.710496	1421.802235	684.9578839	2127.905081	1867.436364	679.4579308	2561.812245	1908.105075	1843.214229	914.6023963
	Rank	2	7	3	8	10	4	5	9	6	**1**
F10	Best	4797.20435	7584.517976	3626.393364	7221.204378	7983.479659	4509.940385	6587.553709	7179.271583	8159.844353	5258.747896
	Mean	7000.03612	8810.345365	**5473.324554**	8830.273731	9293.530654	5960.60294	8381.174816	8826.488225	8770.669714	6342.977685
	Std	1440.153502	451.1483359	659.1312869	580.897944	565.5125342	642.9021187	814.4829474	596.4930339	264.0197474	386.2278063
	Rank	4	7	**1**	9	10	2	5	8	6	3
F11	Best	1664.664235	3916.163713	1317.681405	5119.339774	5382.46485	1475.188918	1951.314686	3364.094507	2448.788476	1150.696897
	Mean	3462.818454	9405.364853	2642.523763	7529.74268	11,690.37179	3216.201469	6442.296191	9338.047012	5534.776385	**1229.396383**
	Std	1378.459652	2537.910055	1091.490865	1814.904936	3535.882701	1600.723015	2752.70647	2623.181979	1150.200302	33.83566386
	Rank	4	9	2	7	10	3	6	8	5	**1**
F12	Best	152,529,305.2	5,057,070,713	91,178,471.78	8,536,501,087	7,095,151,195	3,625,901.127	2,998,867,801	665,266,261.2	2,643,622,015	124,699.8864
	Mean	1,004,213,949	13,783,473,687	2,132,793,017	15,629,086,142	18,002,705,220	381,923,866	7,687,492,717	9,931,920,856	6,765,584,358	**922,979.9099**
	Std	1,028,094,348	3,769,461,470	1,815,196,644	2,402,222,392	4,817,502,857	614,036,726.2	3,060,413,239	5,808,735,143	2,861,786,239	540,528.806
	Rank	3	8	4	9	10	2	6	7	5	**1**
F13	Best	455,933.3854	1,568,585,571	39,195.64888	5,375,373,626	1,331,107,112	46,077.88408	19,555,272.27	160,949,894.3	116,903,226.8	1591.645855
	Mean	428,378,828.1	8,673,644,269	433,228,490.9	16,705,123,597	11,744,836,112	113,785,226.7	3,233,813,180	8,854,445,620	5,615,875,476	**12,890.11693**
	Std	903,278,280.7	4,815,203,090	1,105,478,907	5,276,014,159	5,432,070,009	421,858,017.6	3,231,407,191	9,121,538,849	5,463,405,801	8774.709721
	Rank	3	7	4	10	9	2	5	8	6	**1**
F14	Best	37,348.94853	169,227.7261	2483.46244	842,198.482	68,662.75154	20,551.64949	1603.255315	284,613.0225	66,403.79935	2177.807216
	Mean	712,783.4134	6,159,661.316	50,983.54005	5,954,013.606	10,482,388.69	1,166,637.975	599,901.9009	3,770,831.949	1,337,558.942	**12,147.87959**
	Std	805,020.9182	8,892,610.075	56,982.98445	2,827,393.442	11,092,415.42	1,310,690.753	1,352,689.48	5,349,513.361	1,493,395.233	7988.611721
	Rank	4	9	2	8	10	5	3	7	6	**1**
F15	Best	38,983.30716	272,912.3424	5230.356626	9,124,582.99	406,377,170.8	17,305.66852	5414.745642	16,390,943.3	3,146,100.78	1696.201507
	Mean	10,131,200.6	612,977,572	749,615.6182	539,135,401.9	2,011,492,780	21,675,649.79	4,352,675.624	88813`8365.6	67,427,684.81	**3593.548787**
	Std	22,303,238.92	474,890,670.2	4,246,712.007	787,470,834.3	1,128,271,438	83,080,461.12	15,431,630.08	1,343,959,276	309,524,909.6	1889.639797
	Rank	4	8	2	7	10	5	3	9	6	**1**
F16	Best	2452.804583	3893.736028	2426.800398	3565.229658	5266.174774	2802.959969	2990.282794	3259.487565	3479.547512	2445.335457
	Mean	3242.622756	5727.106475	3446.180644	4796.476298	8344.987994	3610.358847	4126.541692	4704.316924	4197.884818	**2872.657485**
	Std	473.7112392	1035.254561	443.0586145	517.5309807	1946.966972	498.213352	732.1544067	610.9373902	411.6243663	169.936261
	Rank	2	9	3	8	10	4	5	7	6	**1**
F17	Best	1886.994962	2557.857263	1991.241008	2744.806491	3362.920004	1978.328365	1966.898987	2418.220545	2298.857093	1851.998756
	Mean	2324.954405	6610.199126	2399.603547	3536.573546	8506.066272	2635.908209	2657.66373	3835.275393	2873.769505	**2008.822045**
	Std	283.3231235	7174.587027	233.7625613	474.650442	10,626.16769	508.066927	405.2889821	3026.899411	279.2523719	94.29431262
	Rank	2	9	3	7	10	4	5	8	6	**1**
F18	Best	32,665.35653	1,446,433.123	59,660.11803	2,002,506.649	3,530,335.415	114,292.0277	13,945.09045	2,234,944.379	1,845,991.16	46,994.06565
	Mean	3,373,471.489	104,923,135.5	847,156.6115	26,293,446.34	188,127,059.5	7,040,567.027	1,972,965.232	49,268,553.72	5,107,338.579	**198,544.3922**
	Std	4,753,182.371	121,368,344.9	1,218,616.533	23,663,197.6	398,187,025.2	8,282,910.487	4,774,599.86	46,987,901.7	3,880,550.144	118,119.6741
	Rank	4	9	2	7	10	6	3	8	5	**1**
F19	Best	40,706.03801	25,963,624.12	19,165.91859	205,363,914.4	59,156,004.43	6918.90897	5639.612561	40,878,643.38	13,846,291.28	2802.100009
	Mean	20,638,316.54	515,153,477.4	1,645,409.263	1,361,578,448	1,376,386,352	9,157,323.188	16,553,521.79	1,630,848,273	373,311,327.8	**5546.952409**
	Std	54,879,525.69	351,293,707.5	1,434,086.424	732,475,946.4	855,768,452.1	28,931,475.42	50,048,051.2	1,600,930,733	384,273,200.8	1749.996856
	Rank	5	7	2	8	9	3	4	10	6	**1**
F20	Best	2201.829529	2449.998902	2352.009298	2723.131073	2700.610499	2509.789112	2388.049682	2556.342365	2983.217409	2225.473386
	Mean	2607.766463	2994.987697	2587.533219	3124.177637	3233.0957	2896.89892	2883.423381	3006.046094	3243.666082	**2423.255725**
	Std	215.273897	225.0695106	142.2973159	178.7934162	207.5622383	186.1083687	238.9397946	207.2155495	128.4369585	73.58733304
	Rank	3	6	2	8	9	5	4	7	10	**1**
F21	Best	2397.022295	2613.948408	2263.934622	2629.115252	2747.317867	2323.99235	2555.56507	2591.688363	2548.173363	2387.378483
	Mean	2487.398228	2716.207562	2569.438467	2728.448113	2830.941735	2576.709811	2657.502874	2689.764514	2627.049776	**2460.342582**
	Std	41.63588194	52.00533722	64.46362738	44.17438015	47.47240808	71.58579289	51.61737712	55.93183283	34.54277222	23.62017442
	Rank	2	8	3	9	10	4	6	7	5	**1**
F22	Best	2885.102434	6794.795932	3231.035567	7829.518828	8575.303416	2849.471831	5126.574288	6057.667638	9036.289253	2302.016301
	Mean	6350.220055	9613.070617	6191.737414	9853.333387	10,550.49926	6830.267506	8827.762995	9456.552457	9988.915981	**2305.917219**
	Std	2512.558818	938.7536597	1608.200823	904.7440602	613.8949942	1920.315287	1394.424251	1168.628218	332.0321064	2.478190825
	Rank	3	7	2	8	10	4	5	6	9	**1**
F23	Best	2787.754	3293.537855	2951.182354	3137.678588	3488.538587	2927.948442	3152.789141	3046.792688	3013.676622	2724.156156
	Mean	2914.900876	3763.600809	3090.747796	3357.362788	3878.896771	3180.222655	3513.776788	3358.893077	3113.210866	**2782.288215**
	Std	59.75428857	238.1665691	94.91315506	111.0938533	228.3893026	121.8285714	222.6159888	153.9419881	52.37644123	25.2410487
	Rank	2	9	3	6	10	5	8	7	4	**1**
F24	Best	2967.538529	3657.618409	3127.685632	3421.029079	3619.84971	3021.908229	3278.389279	3321.814785	3206.962858	2885.63433
	Mean	3089.681567	4112.696436	3264.792249	3640.332085	4084.070312	3308.485828	3813.718507	3545.994749	3323.182493	**2952.558337**
	Std	65.63113581	230.569925	74.2691182	118.587924	259.8989239	121.0879787	289.1224634	172.4694387	54.69220424	27.30747495
	Rank	2	10	3	7	9	4	8	6	5	**1**
F25	Best	3018.130799	3972.724166	3030.66564	4486.894795	4943.555582	2984.845978	3737.367265	3657.6086	3822.329141	2890.308252
	Mean	3243.418012	4977.947932	3378.07015	5349.388682	6012.790846	3242.400319	4880.555848	5693.880953	4714.542258	**2930.148052**
	Std	178.3204217	483.1014792	214.6170972	501.2691526	577.0407561	198.2511869	792.8933458	1169.804725	481.6578356	20.18852097
	Rank	3	7	4	8	10	2	6	9	5	**1**
F26	Best	4713.149596	9684.077892	5053.901669	8416.091511	10,604.74887	3853.568502	7258.409383	7639.342567	6573.653279	2808.875084
	Mean	6082.497428	11,624.42303	8124.673196	9709.579057	13,472.70285	8250.50523	9982.154493	10,831.05498	7359.132695	**3485.265072**
	Std	571.0699919	941.5659495	1312.00284	684.7965567	1196.229833	1183.816969	1061.731157	1578.070436	386.3725918	929.8322859
	Rank	2	9	4	6	10	5	7	8	3	**1**
F27	Best	3250.284736	4018.610599	3268.640984	3702.452824	3954.708726	3278.287749	3631.445312	3591.05168	3580.589431	3202.748395
	Mean	3369.438051	4836.537927	3459.484344	4233.599491	5285.947294	3422.263972	4243.834456	3996.028949	3828.845823	**3220.790723**
	Std	69.78814161	520.9309151	115.5829273	285.9686475	747.0947338	110.53746	418.1235845	267.9705322	124.7613289	10.5175994
	Rank	2	9	4	7	10	3	8	6	5	**1**
F28	Best	3426.112351	5987.191611	3384.283203	5593.21849	5792.557343	3343.03536	4526.30922	4744.443689	3779.796698	3231.295207
	Mean	3978.907255	7411.086242	4316.936753	7215.252903	8515.048376	3717.44342	6362.649967	7431.749307	4994.934175	**3293.337186**
	Std	331.8139284	630.0672162	507.4118941	743.6049597	1041.220627	241.7334549	859.9941313	1254.786127	616.2564886	20.63278467
	Rank	3	8	4	7	10	2	6	9	5	**1**
F29	Best	3819.122837	5245.844564	4122.351253	4662.610888	5751.89334	3611.876427	4572.502333	4804.688423	4341.749439	3612.906093
	Mean	4361.860822	7871.759267	4835.479601	5722.603017	10,677.8678	4786.13218	5607.788074	8173.632841	5002.779564	**3932.304505**
	Std	292.0287729	2081.51807	438.4303817	734.2899181	5075.439376	382.5070882	699.2846269	11,281.98426	260.2814639	149.9794958
	Rank	2	8	4	7	10	3	6	9	5	**1**
F30	Best	2,229,706.588	126,355,129	1,032,047.119	443,025,866.7	42,115,145.66	721,291.0549	2,958,240.473	34,555,050.01	21,115,634	6773.198221
	Mean	48,175,515.24	1,850,002,938	30,064,140.81	1,690,093,743	2,530,541,321	9,544,957.048	248,930,468.8	735,674,389.5	89,478,576.91	**14,160.5348**
	Std	34,953,894.66	1,182,812,975	100,439,950.7	810,700,101.7	1,619,519,232	9,087,292.424	398,606,089.6	1,283,425,955	32,979,068.97	10,763.07795
	Rank	4	9	3	8	10	2	6	7	5	**1**
Average		2.827586207	8.068965517	3.034482759	7.586206897	9.827586207	3.482758621	5.724137931	7.689655172	5.655172414	**1.103448276**
Total Rank		2	9	3	7	10	4	6	8	5	**1**

The overall ranking performance of DMARS_WGO is high but the performance of the product does not prevail in all the individual benchmark functions. These differences should be observed as per the No Free Lunch principle. Some functions can have a natural preference to less complex or more exploitation-oriented strategies. Nevertheless, the uniformity of the superiority of average ranks and statistical validation of the entire benchmark collection demonstrate strong level of performance at the system level instead of single improvements.

**Fig. 6:**
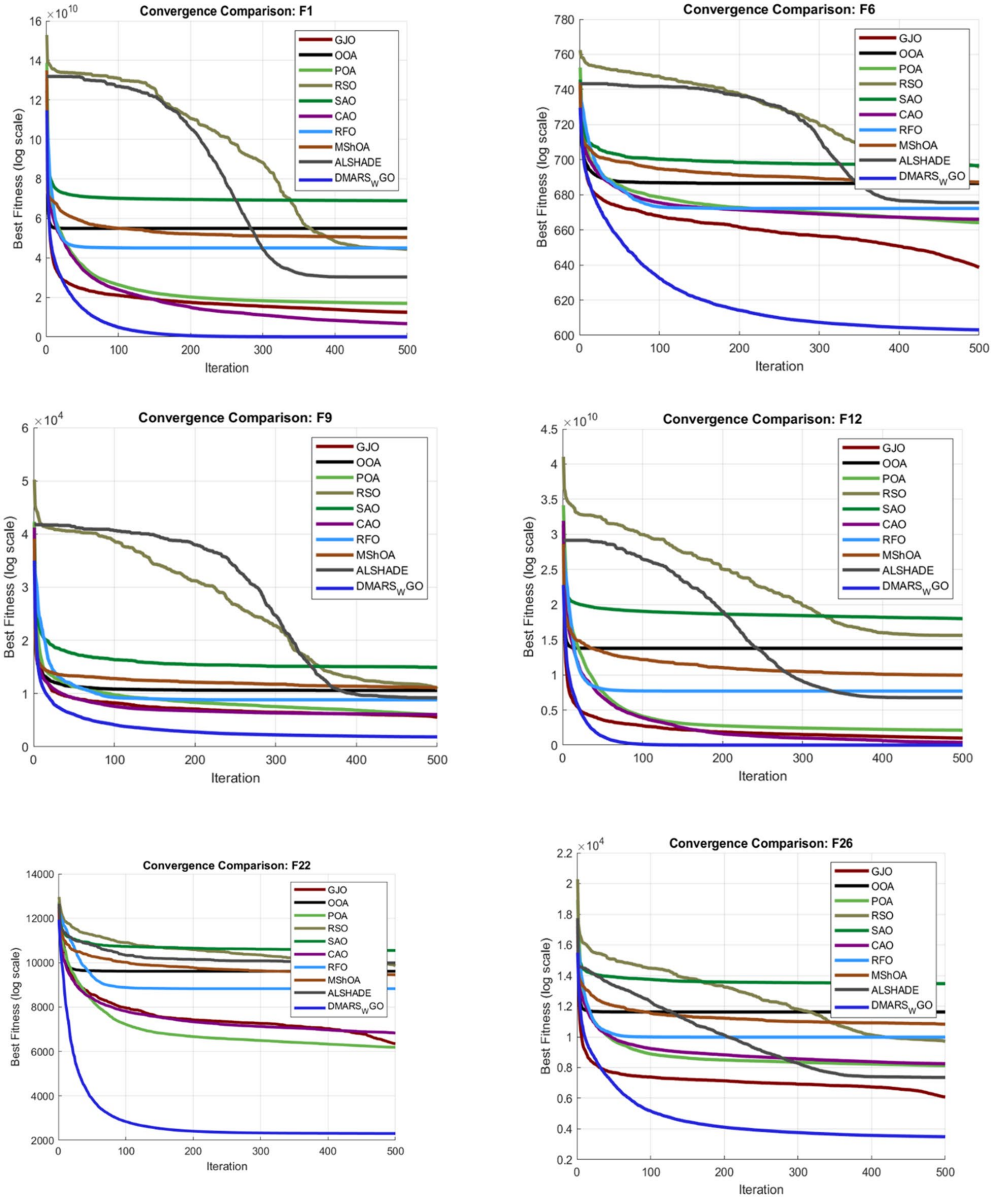
Samples of convergence plots of DMARS_WGO vs other algorithms on CEC 2017.

**Fig. 7:**
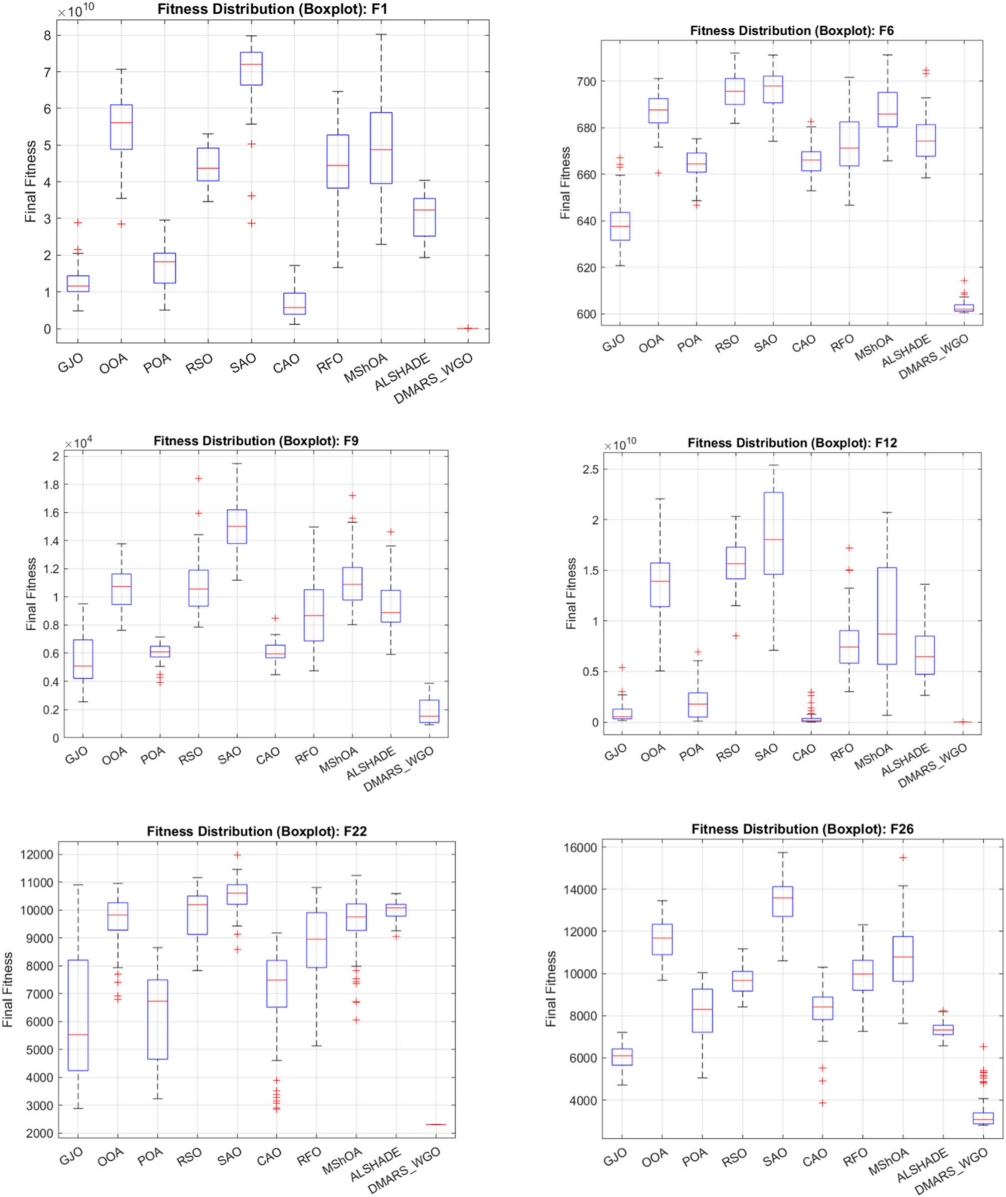
Samples of boxplot of DMARS_WGO vs other algorithms on CEC 2017.

In addition, the Wilcoxon results confirm that the observed performance advantages of DMARS_WGO are statistically significant (*p* < 0.05) across most benchmark cases as shown as in Table 6, indicating that its improvements are both reliable and reproducible. Figure 6 also presents the findings about the convergence of the various techniques. By studying these curves, we can visually compare the convergence rates of DMARS_WGO to other algorithms. Figure 7 shows Boxplot comparison of DMARS_WGO with the compared algorithms on benchmark functions, confirming its consistent and robust performance across all test functions. These tables and curves make it clear that the DMARS_WGO has satisfactorily resolved the majority of the problems. The low standard value found for the great majority of the problems demonstrates the dependability of the DMARS_WGO. Consequently, the proposed DMARS_WGO has been validated to use in-depth statistical and experimental research to determine the global optimum with higher accuracy.

**Table 6:** The Wilcoxon rank-sum test (*p*-value) for DMARS_WGO Vs all other algorithms for CEC 2017.

DMARS vs	GJO	OOA	POA	RSO	SAO	CAO	RFO	MShOA	ALSHADE
F1	3.30368E − 18***	3.30368E − 18***	3.30368E − 18***	3.30368E − 18***	3.30368E − 18***	3.30368E − 18***	3.30368E − 18***	3.30368E − 18***	3.30368E − 18***
F3	0.04395655*	4.18122E − 18***	3.82573E − 13***	2.87427E − 16***	3.30368E − 18***	0.000264778***	2.84794E − 15***	3.30368E − 18***	3.30368E − 18***
F4	3.30368E − 18***	3.30368E − 18***	3.30368E − 18***	3.30368E − 18***	3.30368E − 18***	3.30368E − 18***	3.30368E − 18***	3.30368E − 18***	3.30368E − 18***
F5	0.001620091**	3.30368E − 18***	4.24085E − 17***	3.30368E − 18***	3.30368E − 18***	2.89532E − 14***	3.30368E − 18***	3.30368E − 18***	3.30368E − 18***
F6	3.30368E − 18***	3.30368E − 18***	3.30368E − 18***	3.30368E − 18***	3.30368E − 18***	3.30368E − 18***	3.30368E − 18***	3.30368E − 18***	3.30368E − 18***
F7	1.47143E − 16***	3.30368E − 18***	3.30368E − 18***	3.30368E − 18***	3.30368E − 18***	3.30368E − 18***	3.30368E − 18***	3.30368E − 18***	3.30368E − 18***
F8	0.639434568 ns	3.30368E − 18***	1.91994E − 07***	3.30368E − 18***	3.30368E − 18***	4.38415E − 08***	5.60729E − 18***	3.30368E − 18***	3.30368E − 18***
F9	3.5732E − 17***	3.30368E − 18***	3.30368E − 18***	3.30368E − 18***	3.30368E − 18***	3.30368E − 18***	3.30368E − 18***	3.30368E − 18***	3.30368E − 18***
F10	0.17006188 ns	3.30368E − 18***	1.26122E − 11***	3.30368E − 18***	3.30368E − 18***	0.00061104***	8.44063E − 18***	3.30368E − 18***	3.30368E − 18***
F11	3.30368E − 18***	3.30368E − 18***	3.30368E − 18***	3.30368E − 18***	3.30368E − 18***	3.30368E − 18***	3.30368E − 18***	3.30368E − 18***	3.30368E − 18***
F12	3.30368E − 18***	3.30368E − 18***	3.30368E − 18***	3.30368E − 18***	3.30368E − 18***	3.30368E − 18***	3.30368E − 18***	3.30368E − 18***	3.30368E − 18***
F13	3.30368E − 18***	3.30368E − 18***	3.50432E − 18***	3.30368E − 18***	3.30368E − 18***	3.30368E − 18***	3.30368E − 18***	3.30368E − 18***	3.30368E − 18***
F14	3.50432E − 18***	3.30368E − 18***	2.79404E − 05***	3.30368E − 18***	3.30368E − 18***	4.9869E − 18***	0.010368035*	3.30368E − 18***	3.30368E − 18***
F15	3.30368E − 18***	3.30368E − 18***	5.60729E − 18***	3.30368E − 18***	3.30368E − 18***	3.30368E − 18***	7.51142E − 18***	3.30368E − 18***	3.30368E − 18***
F16	1.63178E − 05***	3.30368E − 18***	5.67605E − 13***	3.30368E − 18***	3.30368E − 18***	3.38078E − 14***	1.59806E − 17***	3.30368E − 18***	3.30368E − 18***
F17	3.01826E − 10***	3.30368E − 18***	4.36929E − 15***	3.30368E − 18***	3.30368E − 18***	1.64587E − 16***	4.24085E − 17***	3.30368E − 18***	3.30368E − 18***
F18	1.72208E − 14***	3.30368E − 18***	1.10312E − 06***	3.30368E − 18***	3.30368E − 18***	1.84067E − 16***	0.024957691*	3.30368E − 18***	3.30368E − 18***
F19	3.30368E − 18***	3.30368E − 18***	3.30368E − 18***	3.30368E − 18***	3.30368E − 18***	4.9869E − 18***	1.19639E − 17***	3.30368E − 18***	3.30368E − 18***
F20	1.59722E − 06***	9.48312E − 18***	2.18688E − 09***	3.30368E − 18***	3.30368E − 18***	4.9869E − 18***	3.58792E − 16***	3.71697E − 18***	3.30368E − 18***
F21	0.000453226***	3.30368E − 18***	1.47143E − 16***	3.30368E − 18***	3.30368E − 18***	1.66132E − 15***	3.30368E − 18***	3.30368E − 18***	3.30368E − 18***
F22	3.30368E − 18***	3.30368E − 18***	3.30368E − 18***	3.30368E − 18***	3.30368E − 18***	3.30368E − 18***	3.30368E − 18***	3.30368E − 18***	3.30368E − 18***
F23	1.69309E − 17***	3.30368E − 18***	3.30368E − 18***	3.30368E − 18***	3.30368E − 18***	3.30368E − 18***	3.30368E − 18***	3.30368E − 18***	3.30368E − 18***
F24	2.13223E − 17***	3.30368E − 18***	3.30368E − 18***	3.30368E − 18***	3.30368E − 18***	3.30368E − 18***	3.30368E − 18***	3.30368E − 18***	3.30368E − 18***
F25	3.30368E − 18***	3.30368E − 18***	3.30368E − 18***	3.30368E − 18***	3.30368E − 18***	3.30368E − 18***	3.30368E − 18***	3.30368E − 18***	3.30368E − 18***
F26	1.2434E − 16***	3.30368E − 18***	7.96266E − 18***	3.30368E − 18***	3.30368E − 18***	1.0051E − 17***	3.30368E − 18***	3.30368E − 18***	3.30368E − 18***
F27	3.30368E − 18***	3.30368E − 18***	3.30368E − 18***	3.30368E − 18***	3.30368E − 18***	3.30368E − 18***	3.30368E − 18***	3.30368E − 18***	3.30368E − 18***
F28	3.30368E − 18***	3.30368E − 18***	3.30368E − 18***	3.30368E − 18***	3.30368E − 18***	3.30368E − 18***	3.30368E − 18***	3.30368E − 18***	3.30368E − 18***
F29	3.4649E − 13***	3.30368E − 18***	5.94546E − 18***	3.30368E − 18***	3.30368E − 18***	2.30094E − 16***	3.30368E − 18***	3.30368E − 18***	3.50432E − 18***
F30	3.30368E − 18***	3.30368E − 18***	3.30368E − 18***	3.30368E − 18***	3.30368E − 18***	3.30368E − 18***	3.30368E − 18***	3.30368E − 18***	3.30368E − 18***

*denotes statistical significance at *p* < 0.05, while multiple asterisks indicate stronger significance levels.

#### Diversity regulation discussion

DMARS_WGO includes a clear diversity aware control system. At initial search phases, GOA exposed search dynamics ensure spatial dispersion among agents, which keeps a large population diversity. As potentially rich areas are discovered, the exploitation strategy of WO further and further eliminates the diversity in order to narrow the convergence. Moreover, the adaptive mutation operator is also attracted when stagnation is identified, and guided stuttering is brought back to the population to avoid untimely failure. As opposed to the predefined schedules of exploration in static metaheuristics, the two-mode reinforcement mechanism constantly measures diversity and improvement rate, varying the dominance of behavioral responses in response to the information. This has the effect of giving a more refined diversity decay trend, and better strength when dealing with multimodal and composition functions, where premature convergence is prevalent in conventional algorithms.

### Simulation results of CEC 2022 benchmark functions

After evaluating the proposed algorithms on the CEC 2017 benchmark functions, additional experiments are carried out on the more recent CEC 2022 functions to validate performance consistency and scalability across different benchmark generations. The CEC 2022 benchmark introduces more complex and dynamically shifted landscapes, providing a stricter evaluation environment for global optimizers^78^. It includes 12 functions which are one unimodal function (F1), three basic functions (F2-F5), three hybrid functions (F6-F8), and four composite functions (F9-F12). Table 7 demonstrates the details of these functions.

**Table 7:** Details of CEC-2022 benchmark functions.

No	Function name	Type	optimal
F1	Shifted and full rotated Zakharov function	Unimodal	300
F2	Shifted and full rotated Rosenbrock’s function	Basic	400
F3	Shifted and full rotated expanded Schafer’s f6 function		600
F4	Shifted and full rotated non-continuous Rastrigin’s function		800
F5	Shifted and rotated Levy function		900
F6	Hybrid function 1 (N-3)	Hybrid	1800
F7	Hybrid function 2 (N-6)		2000
F8	F8 Hybrid function 3 (N-5)		2200
F9	Composite function 1 (N-5)	Composite	2300
F10	Composite function 2 (N- 4)		2400
F11	Composite function 3 (N- 5)		2600
F12	Composite function 4 (N-6)		2700

#### DMARS_WGO vs AIRE_WGO, GOA, and WO

**Fig. 8:**
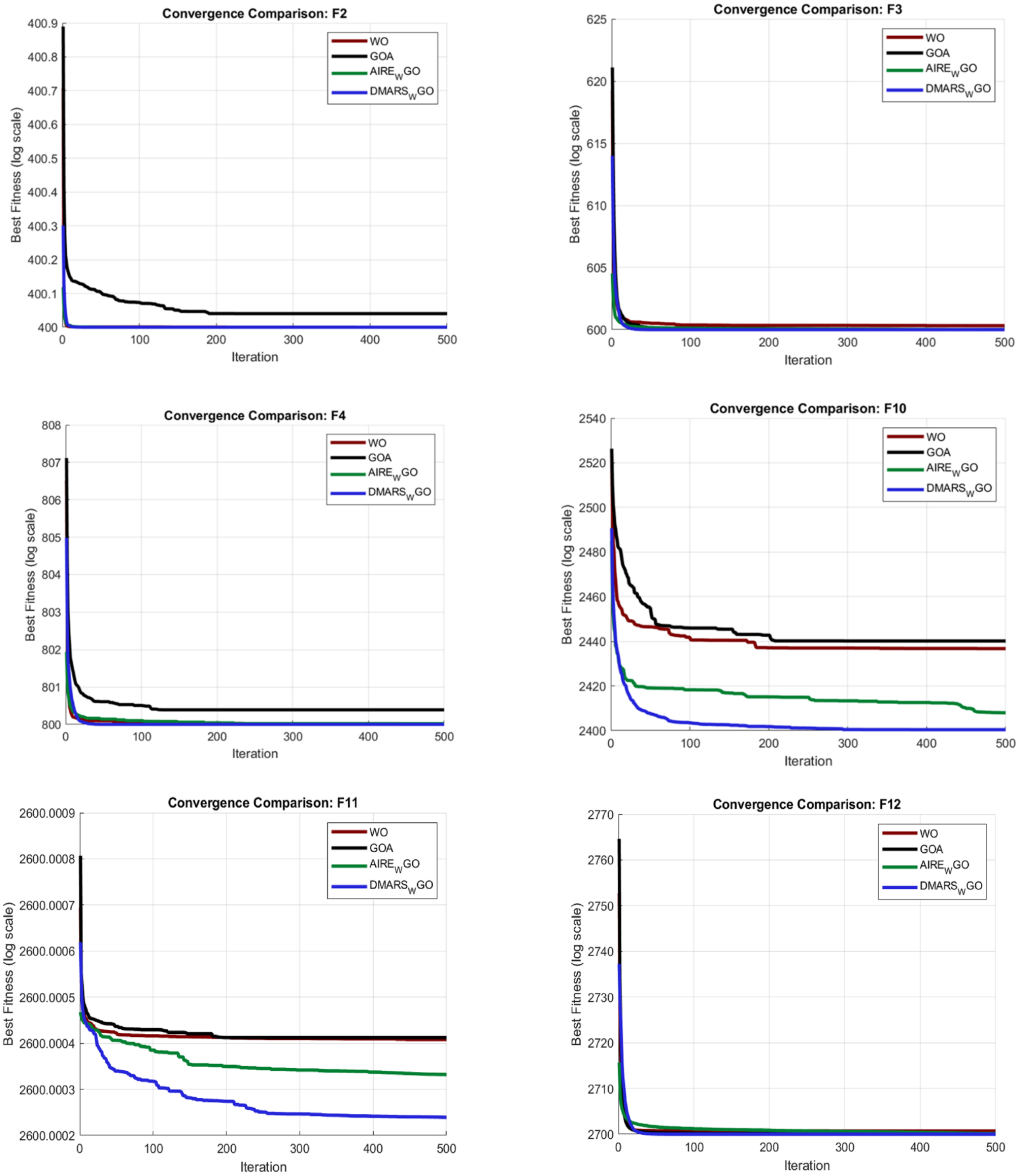
Samples of convergence plots of DMARS_WGO, AIRE_WGO, GOA and WO on CEC 2022.

**Fig. 9:**
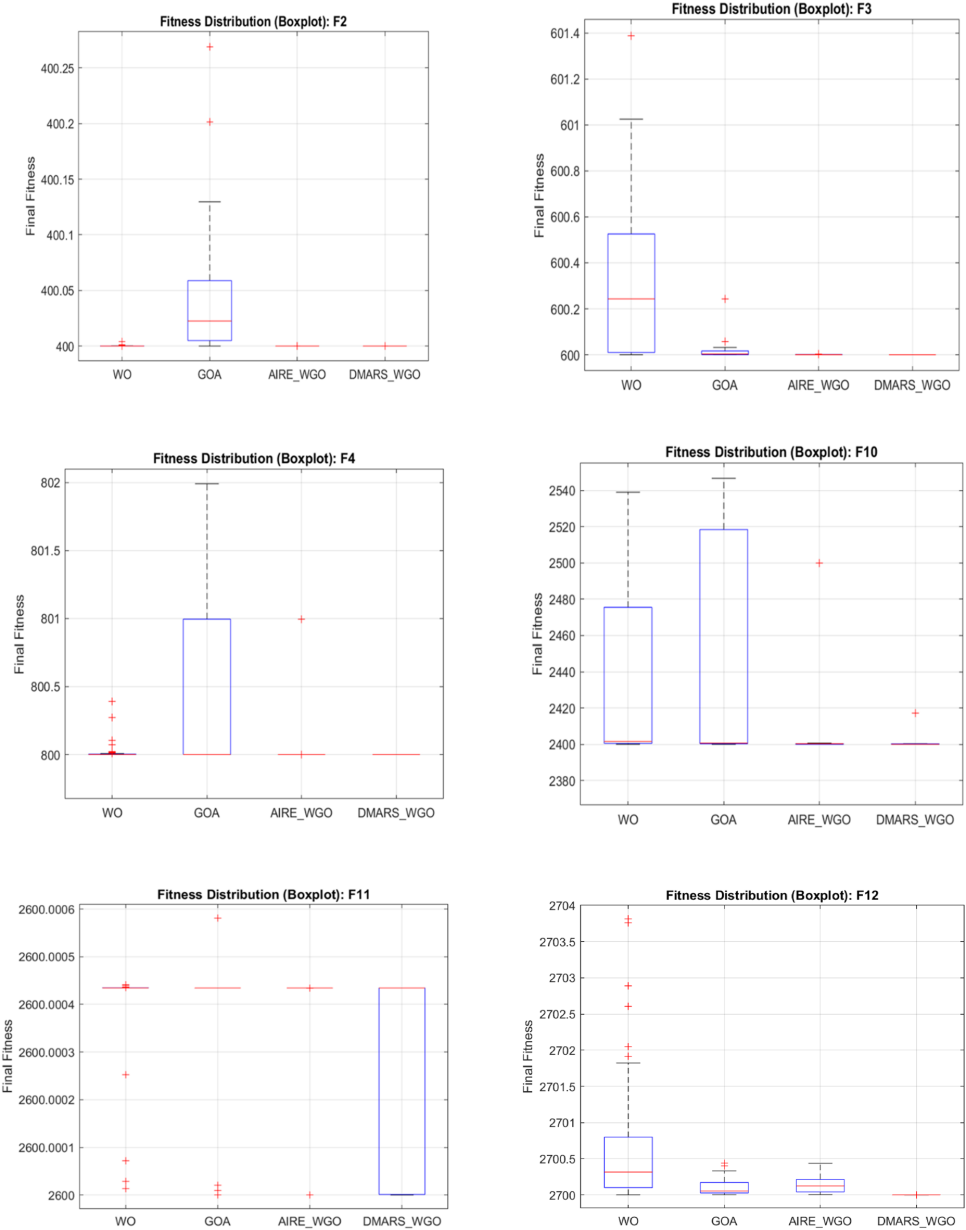
Samples of boxplot of DMARS_WGO, AIRE_WGO, GOA and WO on CEC 2022.

The proposed algorithms are tested on 12 representative benchmark functions from the CEC 2022. This comparison with AIRE_WGO, GOA, and WO highlights the consistency of DMARS_WGO across different problem complexities and benchmark generations. Table 8 shows the best, Mean, STD, and Rank of the achieved results. The best values for each benchmark are shown in bold in the table. DMARS_WGO accomplishes the best mean values for all functions, and it ranks first in all functions. Similarly, the Wilcoxon signed-rank test is applied to the comparative experiments carried out on the CEC 2022 benchmark functions. Table 9 indicates that DMARS_WGO consistently achieves statistically significant improvements (*p* < 0.05) compared to other algorithms. As illustrated in Fig. 8, the convergence plots indicate that DMARS_WGO reaches optimal regions more rapidly and maintains a stable convergence throughout the iterative process. Figure 9 shows a boxplot comparison of DMARS_WGO and the compared algorithms on the benchmark functions, illustrating the higher stability and lower result dispersion of DMARS_WGO.

**Table 8:** Comparison of results of DMARS_WGO, AIRE_WGO, GOA and WO on CEC 2022.

F	Algorithm	WO	GOA	AIRE_WGO	DMARS_WGO
F1	Best	300	300	300	300
	Mean	300.0112503	300.0000368	300.0000001	**300**
	Std	0.042996793	7.83685E − 05	1.79887E − 07	0
	Rank	4	3	2	**1**
F2	Best	400	400.0000216	400	400
	Mean	400.0001737	400.0401962	400.00000000831	**400**
	Std	0.000574001	0.05273098	2.16325E − 08	2.41166E-14
	Rank	3	4	**2**	**1**
F3	Best	600.000008	600.0000025	600.0000065	600
	Mean	600.3250727	600.0145295	600.0006982	**600**
	Std	0.357007191	0.03559655	0.000871659	0
	Rank	4	3	2	**1**
F4	Best	800	800	800	800
	Mean	800.0185378	800.3904599	800.019509	**800**
	Std	0.067260113	0.529435353	0.139322135	0
	Rank	2	4	3	**1**
F5	Best	900	900	900	900
	Mean	900.0410121	900.00000000029	900.00000000115	**900**
	Std	0.085923236	5.43308E − 10	2.45285E − 09	0
	Rank	4	2	3	**1**
F6	Best	1800	1800	1800	1800
	Mean	**1800**	**1800**	**1800**	**1800**
	Std	0	0	0	0
	Rank	**1**	**1**	**1**	**1**
F7	Best	2000	2000	2000	2000
	Mean	**2000**	**2000**	**2000**	**2000**
	Std	0	0	0	0
	Rank	**1**	**1**	**1**	**1**
F8	Best	2201.718282	2201.718282	2201.718282	2201.718282
	Mean	**2201.718282**	**2201.718282**	**2201.718282**	**2201.718282**
	Std	4.59272E − 13	4.59272E − 13	4.59272E − 13	4.59272E-13
	Rank	**1**	**1**	**1**	**1**
F9	Best	2300	2300.000003	2300.000001	2300
	Mean	2300.115246	2304.902414	2300.000192	**2300**
	Std	0.349151334	35.00693738	0.000236812	0
	Rank	3	4	2	**1**
F10	Best	2400.005041	2400.000001	2400	2400
	Mean	2436.773632	2440.198182	2407.992262	**2400.408341**
	Std	53.09661308	60.80883449	27.11224587	2.38427291
	Rank	3	4	2	**1**
F11	Best	2600.000013	2600.000001	2600	2600
	Mean	2600.000408	2600.000412	2600.000332	**2600.000239**
	Std	9.70972E − 05	0.000103619	0.000186122	0.000217691
	Rank	3	4	2	**1**
F12	Best	2700.00107	2700.00266	2700.004222	2700.000091
	Mean	2700.699055	2700.105141	2700.139799	**2700.000398**
	Std	0.942898531	0.104837629	0.11007597	0.000162968
	Rank	4	2	3	**1**
Average		2.75	2.75	2	**1**
Total Rank		3	3	2	**1**

**Table 9:** the Wilcoxon rank-sum test (*p*-value) for DMARS_WGO Vs AIRE_WGO, GOA and WO for CEC 2022.

DMARS vs	WO	GOA	AIRE_WGO
F1	2.51783E − 18***	1.39059E − 20***	1.39059E − 20***
F2	4.27906E − 19***	9.00125E − 20***	9.59038E − 20***
F3	1.36758E − 20***	1.3898E − 20***	1.39059E − 20***
F4	1.96372E − 19***	1.39059E − 20***	1.3902E − 20***
F5	1.96372E − 19***	5.29018E − 20***	1.39059E − 20***
F6	ns	ns	ns
F7	ns	ns	ns
F8	ns	ns	ns
F9	1.96372E − 19***	1.39059E − 20***	1.39059E − 20***
F10	7.78765E − 17***	2.41475E − 13***	0.230808332ns
F11	3.71688E − 15***	2.9636E − 06***	0.013293906*
F12	3.30368E − 18***	3.30368E − 18***	3.30368E − 18***

*denotes statistical significance at *p* < 0.05, while multiple asterisks indicate stronger significance levels.

#### DMARS_WGO vs other algorithms

**Fig. 10:**
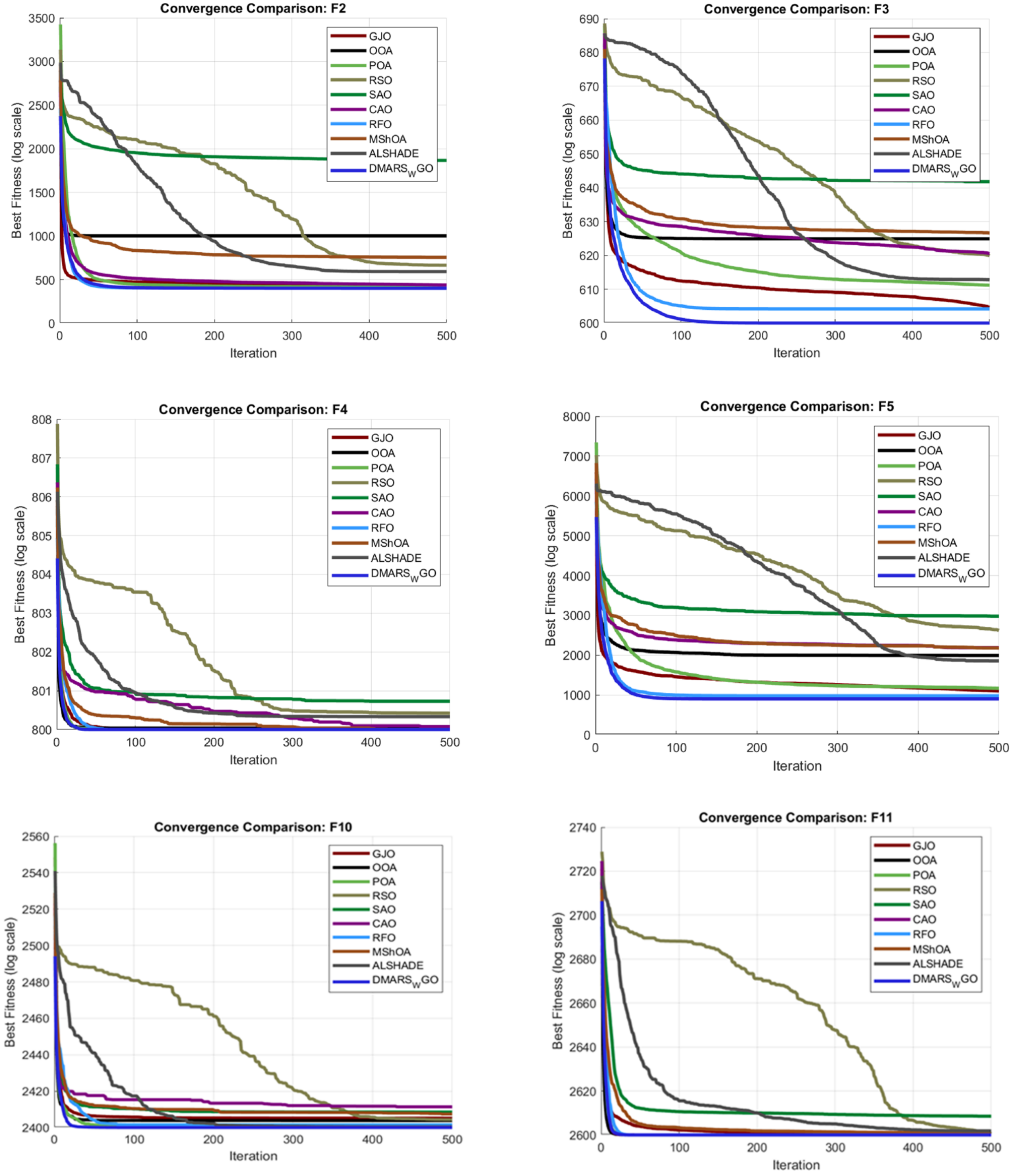
Samples of convergence plots of DMARS_WGO vs other algorithms on CEC 2022.

**Fig. 11:**
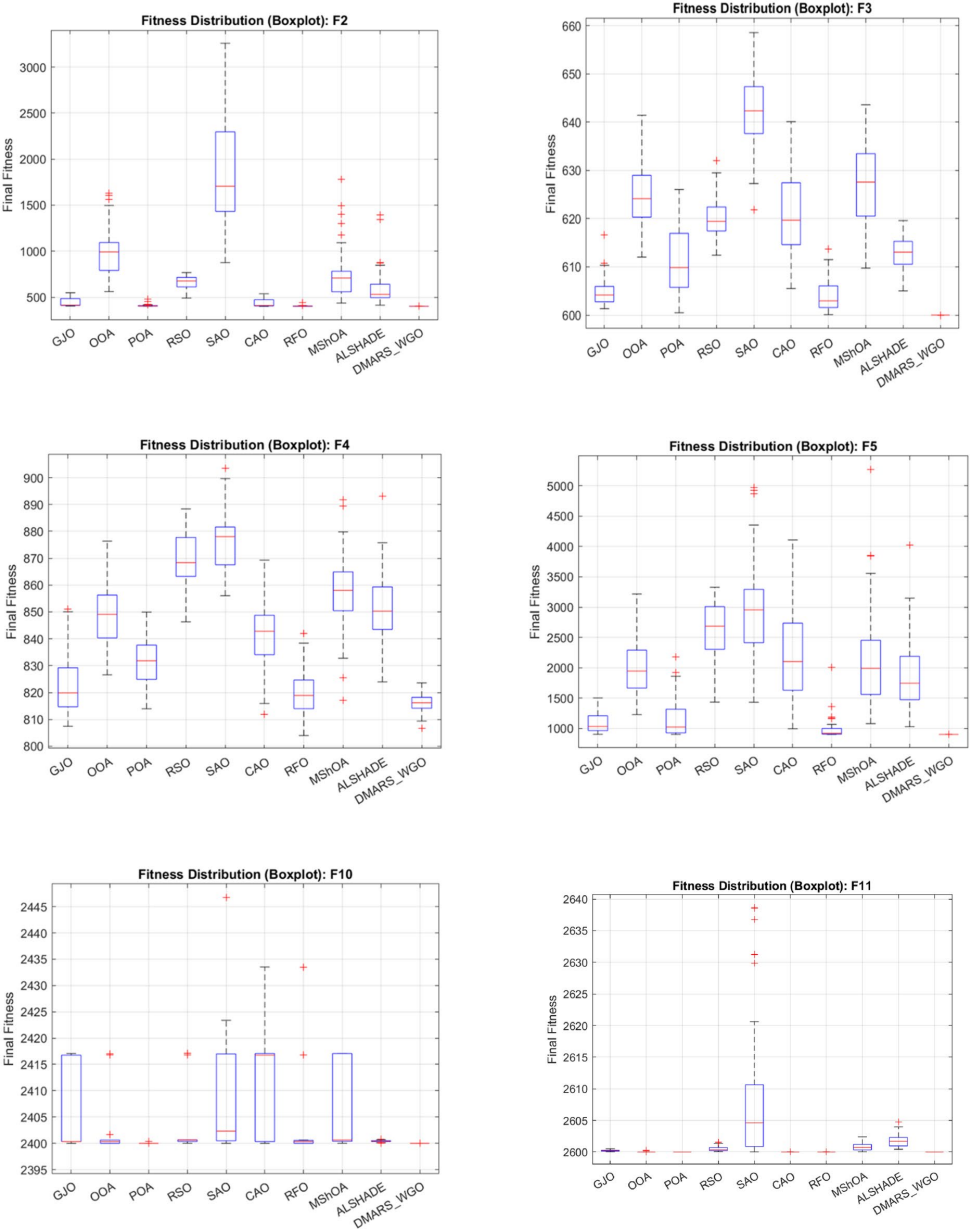
Samples of boxplot of DMARS_WGO vs other algorithms on CEC 2022.

In this section the proposed DMARS_WGO is compared with various optimization algorithms, namely GJO, OOA, POA, RSO, SAO, CAO, RFO, MShOA, and ALSHADE as done in CEC 2017. The outcomes of DMARS_WGO and other algorithms is shown in Table 10. The best, mean, std, and rank are recorded in the table to compare the algorithms. The table displays the best values for each benchmark in bold. DMARS_WGO achieves the best mean values for all benchmark functions except F1, F6, F7, and F12. RFO records the best mean values on F1, F6, and F12, while POA achieves the best mean values on F7. However, DMARS_WGO achieves the overall best total rank (Rank = 1), confirming its superior consistency and robustness across the entire benchmark functions. Additionally, when tested against other state of the art optimization algorithms, the Wilcoxon analysis similarly indicates significant superiority of DMARS_WGO (*p* < 0.05) across most benchmark functions as shown as in Table 11, validating the consistency and statistical soundness of its results. These statistical findings are further supported by the convergence plots are illustrated in Fig. 10. The curves illustrate that DMARS_WGO not only converges faster but also maintains a smoother and more stable optimization trajectory compared to the other algorithms. Figure 11 shows Boxplot comparison of DMARS_WGO against other state of the art optimization algorithms on the benchmarks, demonstrating its consistent and robust performance across diverse test functions.

**Table 10:** Comparison of results of DMARS_WGO vs other algorithms on CEC 2022.

F	Algo	GJO	OOA	POA	RSO	SAO	CAO	RFO	MShOA	ALSHADE	DMARS_WGO
F1	Best	1294.906416	6878.818737	304.5438776	7739.1967	8158.69375	300.445138	300	2768.409165	2035.372249	337.3093807
	Mean	6279.391276	15,762.63268	1955.7729	17,960.15474	19,916.25942	5438.688713	**300.9182731**	18,348.78765	6878.72054	604.9296972
	Std	3938.400463	5457.72803	2011.214503	5454.780956	6657.216634	4417.090695	5.88614366	8686.267484	3931.64263	156.0241119
	Rank	5	7	3	8	10	4	**1**	9	6	2
F2	Best	402.9771069	559.9300443	400.1786833	490.1051513	875.4352086	400.0758833	400	434.8615363	412.7424301	400.0014519
	Mean	438.7980839	1002.438436	410.4167311	664.9184472	1867.045258	437.9042035	403.0566262	755.9623437	592.6357662	**400.7798342**
	Std	44.06875411	262.8498091	17.95035361	66.00871646	609.6902234	43.17690871	5.567586122	273.3901702	192.8027678	0.812232606
	Rank	5	9	3	7	10	4	2	8	6	**1**
F3	Best	601.3312286	612.0336325	600.4961594	612.4058948	621.8850144	605.4992153	600.0919389	609.7353415	605.0130149	600
	Mean	604.6832101	624.9035612	611.204136	619.9089947	641.8174811	620.5459793	604.1884636	626.6580111	612.8176437	**600.0000005**
	Std	2.764699271	7.077481695	6.859673254	4.312926187	7.106397372	7.578819864	3.506364859	9.079396484	3.227689254	8.50874E-07
	Rank	3	8	4	6	10	7	2	9	5	**1**
F4	Best	807.4213744	826.5540713	813.9417584	846.339551	856.0265875	811.9400086	803.9799523	817.1558832	823.9525743	806.6101914
	Mean	822.7970746	848.801711	831.4610629	869.551349	876.0004275	842.0712334	819.9691539	856.7758232	850.9284627	**816.0758686**
	Std	10.6054696	10.8886191	8.564448825	9.405195001	11.04240905	12.24482547	8.510224737	14.45099446	12.70507846	3.305620985
	Rank	3	6	4	9	10	5	2	8	7	**1**
F5	Best	901.7135584	1227.779392	901.1998556	1433.866873	1431.458828	994.9547334	900.0000793	1078.393413	1028.306921	900
	Mean	1098.47049	1992.498123	1169.184854	2621.618349	2976.623457	2184.907077	980.1900716	2179.14005	1854.262128	**900.0017555**
	Std	171.5630421	448.0326433	306.2926848	451.3439654	770.411653	737.2922556	172.9692853	851.6483296	599.326805	0.012536467
	Rank	3	6	4	9	10	8	2	7	5	**1**
F6	Best	2660.542852	1921.559114	1814.801525	2,741,092.151	113,019.7969	1894.3742	1801.775936	22,501.5732	121,802.4212	1852.123294
	Mean	1,369,375.756	5,295,597.332	2016.87665	31,687,346.55	134,120,795.6	5550.189843	**1828.761629**	10,667,136.26	1,229,328.934	1890.60124
	Std	9,709,626.388	11,877,272.91	305.308153	19,318,453.87	194,958,974	4529.267631	20.6289893	27,288,439.5	747,337.4601	44.26107967
	Rank	6	7	3	9	10	4	**1**	8	5	2
F7	Best	2004.696155	2022.991036	2003.358419	2033.723542	2053.302846	2025.263748	2004.73528	2034.286371	2034.363075	2017.099968
	Mean	2035.283379	2058.911621	**2024.746119**	2062.651084	2101.789077	2068.223666	2028.108113	2076.995604	2065.346614	2027.907099
	Std	11.65910357	22.37941651	6.610705018	13.5724736	20.09221971	38.01644966	12.58045515	31.146187	11.41484712	3.339380496
	Rank	4	5	**1**	6	10	8	3	9	7	2
F8	Best	2205.09682	2223.259006	2202.474289	2227.362782	2225.199599	2208.110723	2202.50539	2210.270254	2225.718824	2206.820879
	Mean	2228.70161	2229.886361	2218.579265	2238.179287	2238.63766	2237.849726	2221.968515	2244.884178	2228.867818	**2218.44284**
	Std	12.9795255	3.619969114	8.168167867	10.24077071	6.998178456	15.96183133	8.597766545	15.94332014	1.851671145	4.079359046
	Rank	4	6	2	8	9	7	3	10	5	**1**
F9	Best	2300.003657	2300	2300	2300.10103	2300.176611	2300.000001	2300	2300.019692	2300.036296	2300
	Mean	2300.065481	2300.040978	2300	2302.70306	2316.509606	2300.000093	2300	2300.682849	2301.111826	**2300**
	Std	0.033030377	0.159369271	0	2.407838852	23.37784967	0.000211559	4.1812E − 12	0.524585022	3.93314382	0
	Rank	6	5	1	9	10	4	3	7	8	**1**
F10	Best	2400.000306	2400	2400	2400.003128	2400.000693	2400	2400	2400.009642	2400.024993	2400
	Mean	2405.150082	2403.836139	2400.031384	2404.333015	2408.551476	2411.391839	2401.553013	2407.401421	2400.386556	**2400**
	Std	7.663200224	6.894156133	0.093546951	7.109217144	10.1171371	8.631047749	5.599235674	8.149010762	0.163277962	2.96959E-08
	Rank	7	5	2	6	9	10	4	8	3	**1**
F11	Best	2600.022096	2600	2600	2600.057247	2600.019438	2600.000001	2600	2600.030254	2600.438191	2600
	Mean	2600.21845	2600.013915	2600	2600.498707	2608.513502	2600.000024	2600	2600.883927	2601.808911	**2600**
	Std	0.111248172	0.052101663	0	0.369842198	10.94448464	3.21265E − 05	2.8209E − 12	0.681733277	0.964219549	0
	Rank	6	5	1	7	10	4	3	8	9	**1**
F12	Best	2955.054235	2950	2951.904238	2919.444856	2955.054235	2950	2700.468861	2817.215701	2955.054235	2868.380512
	Mean	2955.089579	2950	2954.992471	2953.367439	2955.371479	2950	**2946.453754**	2946.545629	2955.158187	2951.33714
	Std	0.05048059	0	0.441087869	5.250482286	0.366874286	0	48.7477123	19.44514147	0.014847232	14.71914837
	Rank	8	3	7	6	10	3	**1**	2	9	5
Average		5	6	2.916666667	7.5	9.833333333	5.666666667	2.25	7.75	6.25	**1.583333333**
Total Rank		4	6	3	8	10	5	2	9	7	**1**

**Table 11:** the Wilcoxon rank-sum test (*p*-value) for DMARS_WGO Vs all other algorithms for CEC 2022.

DMARS vs	GJO	OOA	POA	RSO	SAO
F1	3.30368E − 18***	3.30368E − 18***	3.23625E − 05***	3.30368E − 18***	3.30368E − 18***
F2	3.50432E − 18***	3.30368E − 18***	1.9052E − 13***	3.30368E − 18***	3.30368E − 18***
F3	3.30368E − 18***	3.30368E − 18***	3.30368E − 18***	3.30368E − 18***	3.30368E − 18***
F4	0.000641858***	3.30368E − 18***	4.14231E − 15***	3.30368E − 18***	3.30368E − 18***
F5	3.30368E − 18***	3.30368E − 18***	3.30368E − 18***	3.30368E − 18***	3.30368E − 18***
F6	3.30368E − 18***	5.28812E − 18***	0.001987922**	3.30368E − 18***	3.30368E − 18***
F7	0.000553492***	2.87427E − 16***	4.71817E − 05***	3.50432E − 18***	3.30368E − 18***
F8	4.9545E − 12***	3.94236E − 18***	0.014304244*	3.30368E − 18***	3.30368E − 18***
F9	1.39059E − 20***	7.11552E − 19***	ns	1.39059E − 20***	1.39059E − 20***
F10	2.45727E − 18***	5.88644E − 13***	0.003764506**	2.45727E − 18***	2.45727E − 18***
F11	1.39059E − 20***	2.51783E − 18***	ns	1.39059E − 20***	1.39059E − 20***
F12	1.17865E − 07***	8.7785E − 17***	0.16292429ns	0.377632122ns	6.21524E − 18***

DMARS vs	CAO	RFO	MShOA	ALSHADE
F1	4.72938E − 16***	3.50432E − 18***	3.30368E − 18***	3.30368E − 18***
F2	8.49807E − 14***	0.001735093**	3.30368E − 18***	3.30368E − 18***
F3	3.30368E − 18***	3.30368E − 18***	3.30368E − 18***	3.30368E − 18***
F4	6.22712E − 16***	0.019504021*	9.48312E − 18***	3.30368E − 18***
F5	3.30368E − 18***	3.50432E − 18***	3.30368E − 18***	3.30368E − 18***
F6	1.79369E − 17***	1.31525E − 16***	3.30368E − 18***	3.30368E − 18***
F7	4.6085E − 15***	0.031685243*	3.30368E − 18***	3.30368E − 18***
F8	2.87427E − 16***	1.20307E − 05***	5.63717E − 17***	3.30368E − 18***
F9	1.39059E − 20***	1.35833E − 20***	1.39059E − 20***	1.39059E − 20***
F10	1.63952E − 15***	0.000314601***	2.45727E − 18***	2.45727E − 18***
F11	1.39059E − 20***	1.34151E − 20***	1.39059E − 20***	1.39059E − 20***
F12	8.7785E − 17***	3.13939E − 17***	1.12552E − 16***	3.48112E − 22***

*denotes statistical significance at *p* < 0.05, while multiple asterisks indicate stronger significance levels.

### Simulation results of real world problems

In real-world optimization problems, the structure of the search space is often unknown and highly complex, posing multiple challenges for optimization algorithms. These challenges significantly degrade the performance of algorithms that may otherwise perform well on standard benchmark functions or simplified test cases. To ensure high quality solutions in optimization problems, researchers must recognize these challenges and incorporate suitable adjustments and improvements into their algorithms to effectively address them. In this part, the DMARS was evaluated with respect to solving a collection of six real-life design problems in the field of engineering, which are the design of welded beams, the design of pressure vessels, the design of tension/ compression springs, the minimization of weight of a speed reducer, the design of a three-bar truss problem and the design of a gear train problem.

#### Tension/compression spring design

**Fig. 12:**
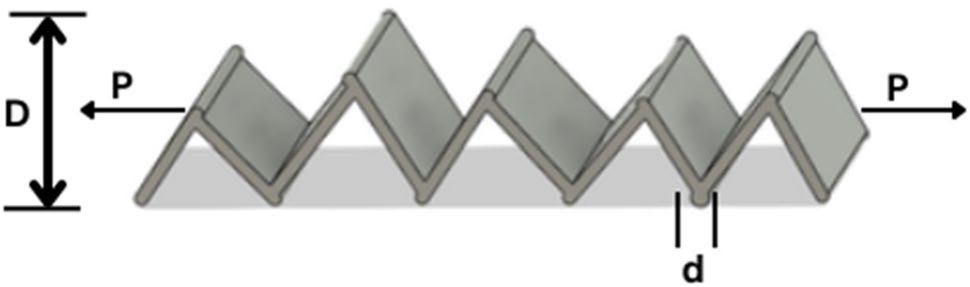
Tension/compression spring design problem.

This is to be achieved by minimizing the weight of the fabrication and this is determined by three structural parameters: the wire diameter (d or $x1$), mean coil diameter (D or ${\mathrm{x2}}$), and the number of active coils (P or ${\mathrm{x3}}$). Figure 12 shows the spring and its parameters^79^.

The following is the mathematical formulation of this problem:

Consider $$X = \left[ {x_{1} , x_{2} ,x_{3} } \right] = \left[ {d, D, P} \right].$$

32 $$ {\mathrm{Minimize}}\,\,\,{\mathrm{f}}\left( {\mathrm{x}} \right) = {\mathrm{x}}_{2} {\mathrm{x}}_{1}^{2} \left( {{\mathrm{x}}_{3} + 2} \right). $$

$$ {\text{According to}}: q_{1} \left( x \right) = 1 - { }\frac{{x_{2}^{3} x_{3} }}{{7.1785x_{1}^{4} }}{ } \le 0, $$

$$ q_{2} \left( x \right) = { }\frac{{4x_{2}^{2} - { }x_{1} x_{2} }}{{12.566\left( {x_{2} x_{1}^{3} } \right){ } - { }x_{1}^{4} }} + { }\frac{1}{{5.108x_{1}^{{2{ }}} }} - 1{ } \le 0, $$

$$ q_{3} \left( x \right) = 1 - { }\frac{{140.45x_{1} }}{{x_{2}^{2} x_{3} }}{ } \le 0, $$

$$ q_{4} \left( x \right) = { }\frac{{x_{1} + { }x_{2} }}{1.5} - 1{ } \le 0. $$

With the following variable bounds: $0.05 \le x_{1} \le 2,{ }0.25 \le x_{2} \le 1.3,{ }and{ }2 \le x_{3} \le 15$.

#### Pressure vessel design

**Fig. 13:**
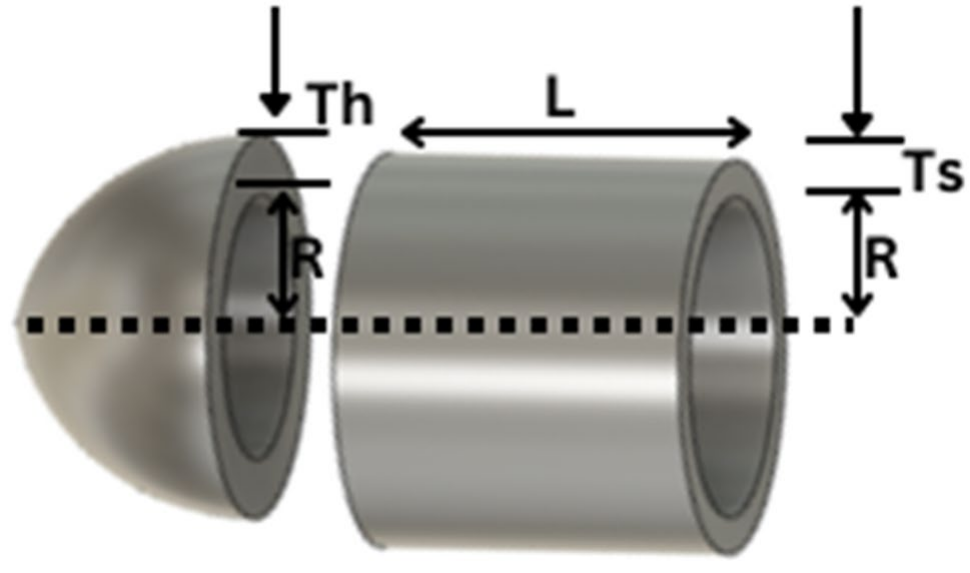
Schematic of pressure vessel design.

It aims at designing a pressure vessel design at the lowest fabrication cost. Figure 13 shows the pressure vessel and the design parameters. The four decision variables in this problem are head thickness (Th), shell thickness (Ts), length of the cylindrical section (L) without considering the head and inner radius (R)^80^.

The mathematical formulation of this problem is the following:

Consider $$X{ } = \left[ {x_{1} ,{ }x_{2} ,{ }x_{3} ,{ }x_{4} } \right] = \left[ {T_{s} ,{ }T_{h} ,{ }R,{ }L} \right].$$

$x_{1} { }and{ }x_{2}$ are discrete while $x_{3} { }and{ }x_{4}$ are continuous.

The fitness is a nonlinear and linear and non-linear inequality constrained fitness.

33 $$ {\mathrm{Minimize}}:{\mathrm{f}}\left( {\mathrm{x}} \right) = 0.6224{\mathrm{x}}_{1} {\mathrm{x}}_{3} {\mathrm{x}}_{4} + 3.1661{\text{ x}}_{1}^{2} {\mathrm{x}}_{4} + 1.778{\mathrm{x}}_{2} {\mathrm{x}}_{3}^{2} + 19.84{\text{ x}}_{1}^{2} {\mathrm{x}}_{3} . $$

$$ {\text{According to}}:\,{\mathrm{q}}1{ }\left( x \right) = - x_{1} + 0.0193x_{3} { } \le { }0,{ } $$

$$ {\mathrm{q2}} \left( {\mathrm{x}} \right) = - x_{2} + 0.00954x_{3} \le 0, $$

$$ {\mathrm{q3}} \left( {\mathrm{x}} \right) \, = - \pi x_{3}^{2} x_{4} - { }\frac{4}{3}\pi x_{3}^{3} + 1296000, $$

$$ {\mathrm{q4}} \left( {\mathrm{x}} \right) \, = x_{4} - 240 \le 0, $$

$$ {\mathrm{With}}\,\,0 \le x_{1} ,{ }x_{2} \le 100,{ }\,{\mathrm{and}}\,{ }10 \le x_{3} ,{ }x_{4} \le 200. $$

#### Welded beam design

**Fig. 14:**
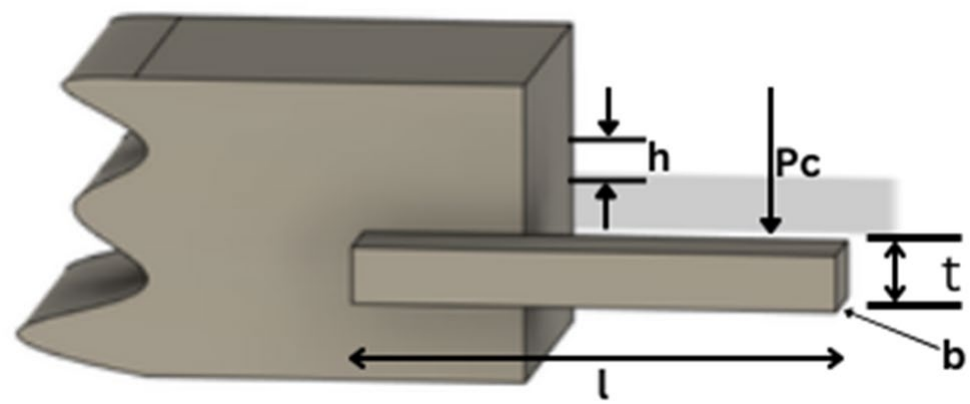
Welded beam design schematic.

One more issue that is often applied to assess the efficiency of DMARS_WGO in the optimization of engineering problems is the so-called welded beam design problem^81^, as illustrated in Fig. 14.

This problem aims at arriving at the best design parameters in order to minimize the total manufacturing cost of a welded beam with a combination of constraints such as the shear stress (τ), bending stress (θ), the buckling load of the bar (Pc), deflection at the beam end (δ) amongst others. The four variables of this problem are the bar length (l), weld thickness (h), height (t) and thickness (b). The mathematical formulation of this problem is the following:

$$ {\text{According to}}\,X{ } = \left[ {x_{1} ,{ }x_{2} ,{ }x_{3} ,{ }x_{4} } \right] = \left[ {h,{ }l,{ }t,{ }b} \right]. $$

Minimize

34 $$ {\mathrm{f}}\left( {\mathrm{x}} \right) = 1.10471{\mathrm{x}}_{1}^{2} {\mathrm{x}}_{2} + 0.04811{\mathrm{x}}_{3} {\mathrm{x}}_{4} \left( {14.0{ } + {\text{ x}}_{2} } \right). $$

$$ {\text{Subject to}}:\,{\mathrm{q}}1{ }\left( x \right) = \tau \left( x \right) - 13600{ } \le { }0,{ } $$

$$ {\mathrm{q2}} \left( {\mathrm{x}} \right) = \sigma \left( {\mathrm{x}} \right) - {3}0000 \le 0, $$

$$ {\mathrm{q3}} \left( {\mathrm{x}} \right) = {\mathrm{x1}}{-}{\mathrm{x4}} \le 0, $$

$$ {\mathrm{q4}} \left( {\mathrm{x}} \right) = 0.{1}0{471} \times {21} + 0.0{4811} \times {3} \times {4} \left( {{14} + {\mathrm{x2}}} \right){-}{5}.0 \le 0, $$

$$ {\mathrm{q5}}\left( {\mathrm{x}} \right) = 0.{125}{-}{\mathrm{x1}} \le 0, $$

$$ {\mathrm{q6}}\left( {\mathrm{x}} \right) = \delta \left( {\mathrm{x}} \right){-}0.{25} \le 0, $$

$$ {\mathrm{q7}} \left( {\mathrm{x}} \right) = {6}000{-}{\mathrm{pc}} \left( {\mathrm{x}} \right) \le 0. $$

$$ Where \, \tau \left( x \right) = \sqrt {\tau^{\prime } + \left( {2\tau \tau^{\prime } } \right)\frac{{x_{2} }}{2R} + \left( {\tau^{\prime \prime } } \right)^{2} } , $$

$$ \tau^{\prime } = \frac{6000}{{\sqrt {2x_{1} x_{2} } }}, $$

$$ \tau^{\prime \prime } = \frac{MR}{J}, $$

$$ {\mathrm{M}} = 6000\left( {14 + \frac{{{\mathrm{x}}_{2} }}{2}} \right),{ } $$

$${\mathrm{R}} = { }\sqrt {\frac{{{\mathrm{x}}_{2}^{2} }}{4} + \left( {\frac{{{\mathrm{x}}_{1} + {\mathrm{x}}_{3} }}{2}} \right)^{2} },$$

$$ {\mathrm{J}} = {2}\left( {{\mathrm{x}}_{1} {\mathrm{x}}_{2} \surd {2}\left[ {\frac{{{\mathrm{x}}_{2}^{2} }}{12} + \left( {\frac{{{\mathrm{x}}_{1} + {\mathrm{x}}_{3} }}{2}} \right)^{2} } \right]} \right) $$

$$ \sigma \left( x \right) = \frac{ 504000}{{x_{4} x_{3}^{2} }} $$

$$ \delta \left( {\mathrm{x}} \right) = \frac{{{ }65856000}}{{\left( {30{ }.{ }10^{6} } \right){\mathrm{x}}_{4} {\mathrm{x}}_{3}^{3} }}{ } $$

$$ {\text{ pc}} \left( {\mathrm{x}} \right) = \frac{{4.013\left( {30{ }.{ }10^{6} } \right)\sqrt {\frac{{{\mathrm{x}}_{3}^{2} {\mathrm{x}}_{4}^{6} }}{36}} }}{196}{ }\left( {1 - \frac{{{\mathrm{x}}_{3} }}{28}\sqrt {\frac{{30{ }.{ }10^{6} }}{{4\left( {12{ }.{ }10^{6} } \right)}}} { }} \right). $$

$$ {\mathrm{With}}\,\,0.1 \le x_{1} ,\,x_{4} \le 2{ }\,{\mathrm{and}}\,\,{ }0.1 \le x_{2} ,\,x_{3} \le 10. $$

#### Speed Reducer problem

**Fig. 15:**
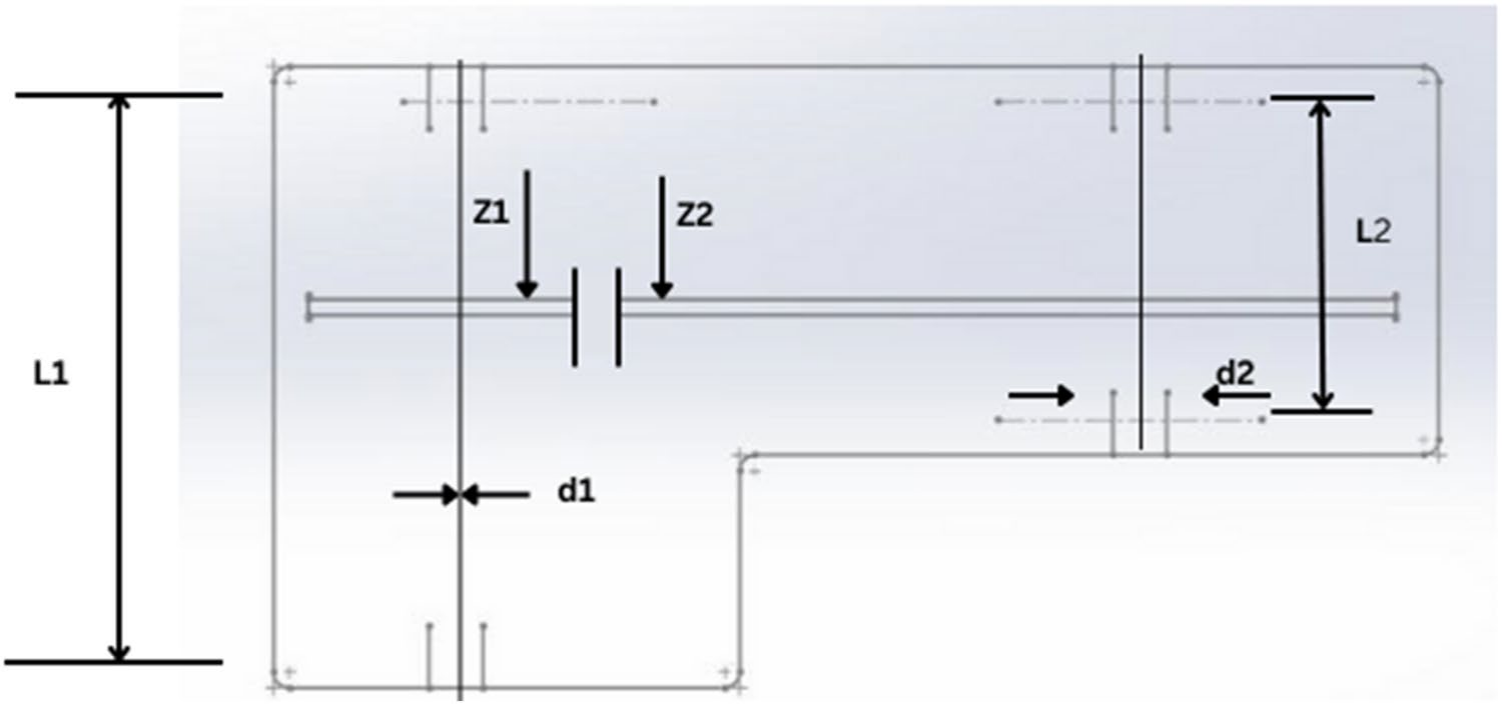
Speed reducer design.

The given issue aims at reducing the total weight of the design of a small aircraft internal combustion engine speed reducer. It is a difficult target given that there are seven design variables ($x_{1} { }to{ }x_{7}$)^82^ as indicated in Fig. 15.

The mathematical formulation of this problem is the following:

According to $$X = \left[ {x_{1} , x_{2} , x_{3} , x_{4} , x_{5} , x_{6} , x_{7} } \right] = \left[ {b, m, p, l_{1} , l_{2} , d_{1} , d_{2} } \right].$$

35 $$ \begin{aligned} & Minimize \\ & {\mathrm{f}}\left( {\mathrm{x}} \right) = 7.4777\left( {{\mathrm{x}}_{6}^{3} + {\mathrm{x}}_{7}^{3} } \right) - 1.508{\mathrm{x}}_{1} \left( {{\mathrm{x}}_{6}^{2} + {\mathrm{x}}_{7}^{2} } \right) + 0.7854\left( {{\mathrm{x}}_{4} {\mathrm{x}}_{6}^{2} + {\mathrm{x}}_{5} {\mathrm{x}}_{7}^{2} } \right) \\ & \,\,\,\,\,\,\,\,\,\,\,\,\,\,\,\,\, + 0.7854{\mathrm{x}}_{1} {\mathrm{x}}_{2}^{2} \left( {3.3333{\mathrm{x}}_{3}^{2} + 14.9334{\mathrm{x}}_{3} - 43.0934} \right) \\ \end{aligned} $$

$$ {\text{Subject to}}q_{1} \left( x \right) = \frac{27}{{x_{1} x_{2}^{2} x_{3} }} - 1 \le 0, $$

$$ q_{2} \left( x \right) = \frac{397.5}{{x_{1} x_{2}^{2} x_{3} }} - 1 \le 0, $$

$$ q_{3} \left( x \right) = \frac{{1.93x_{4}^{3} }}{{x_{2} x_{3} x_{6}^{4} }} - 1 \le 0, $$

$$ q_{4} \left( x \right) = \frac{{1.93x_{5}^{3} }}{{x_{2} x_{3} x_{7}^{4} }} - 1 \le 0, $$

$$ q_{5} \left( x \right) = \frac{1}{{110x_{6}^{3} }}\sqrt {\left( {\frac{{745x_{4} }}{{x_{2} x_{3} }}} \right)^{2} + 16.9 \times 10^{6} } - 1 \le 0, $$

$$ q_{6} \left( x \right) = \frac{1}{{85x_{7}^{3} }}\sqrt {\left( {\frac{{745x_{5} }}{{x_{2} x_{3} }}} \right)^{2} + 157.5 \times 10^{6} } - 1 \le 0, $$

$$ q_{7} \left( x \right) = \frac{{x_{2} x_{3} }}{40} - 1 \le 0, $$

$$ q_{8} \left( x \right) = \frac{{5x_{2} }}{{x_{1} }} - 1 \le 0, $$

$$ q_{9} \left( x \right) = \frac{{x_{1} }}{{12x_{2} }} - 1 \le 0, $$

$$ q_{10} \left( x \right) = \frac{{1.5x_{6} + 1.9}}{{x_{4} }} - 1 \le 0, $$

$$ q_{11} \left( x \right) = \frac{{1.1x_{7} + 1.9}}{{x_{5} }} - 1 \le 0. $$

$$ {\rm With}\,2.6 \le x_{1} \le 3.6, 0.7 \le x_{2} \le 0.8, 17 \le x_{3} \le 28, 7.3 \le x_{4} \le 8.3, 7.3 \le x_{5} \le 8.3, 2.9 \le x_{6} \le 3.9, 5 \le x_{7} \le 5.5.$$

#### Gear train design problem

**Fig. 16:**
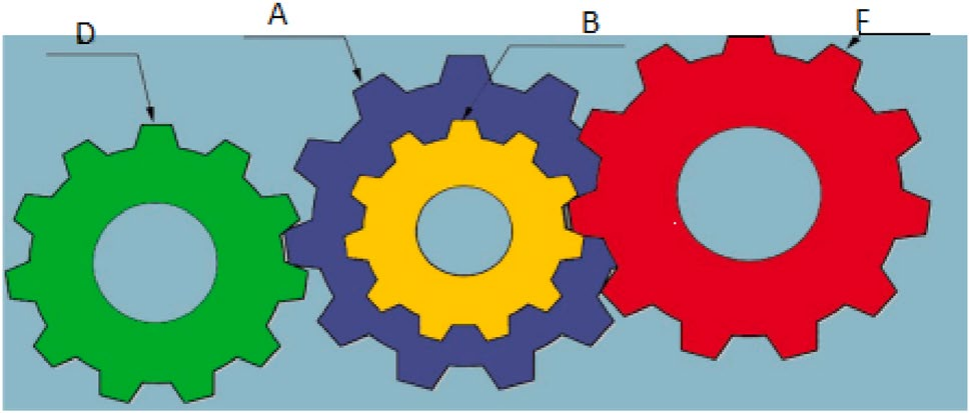
gear train design.

The primary aim of creating this problem is to reduce the gear ratio of preparing the compound gear train^83^ as presented in Fig. 16.

The idea is to have a minimum gear ratio by attaining the optimum number of teeth on the four train gears. The mathematical formulation of this problem is the following:

$$ {\mathrm{According}}\,{\mathrm{to}}\,\,x_{1} , x_{2} ,x_{3} ,x_{4} = n_{A} , n_{B} , n_{D} ,n_{F} $$

36 $$ Minimize\,{\mathrm{f}}\left( {\mathrm{x}} \right) = \left( {\frac{1}{6.931} - \frac{{{\mathrm{x}}_{3} {\mathrm{x}}_{2} }}{{{\mathrm{x}}_{1} {\mathrm{x}}_{4} }}} \right)^{2} $$

$$ {\mathrm{Subject}}\,\,{\mathrm{to}}\,\,12 \le x_{i} \le 60,\, i = 1, \,2, \,3,\, 4. $$

#### Three-bar truss design problem

**Fig. 17:**
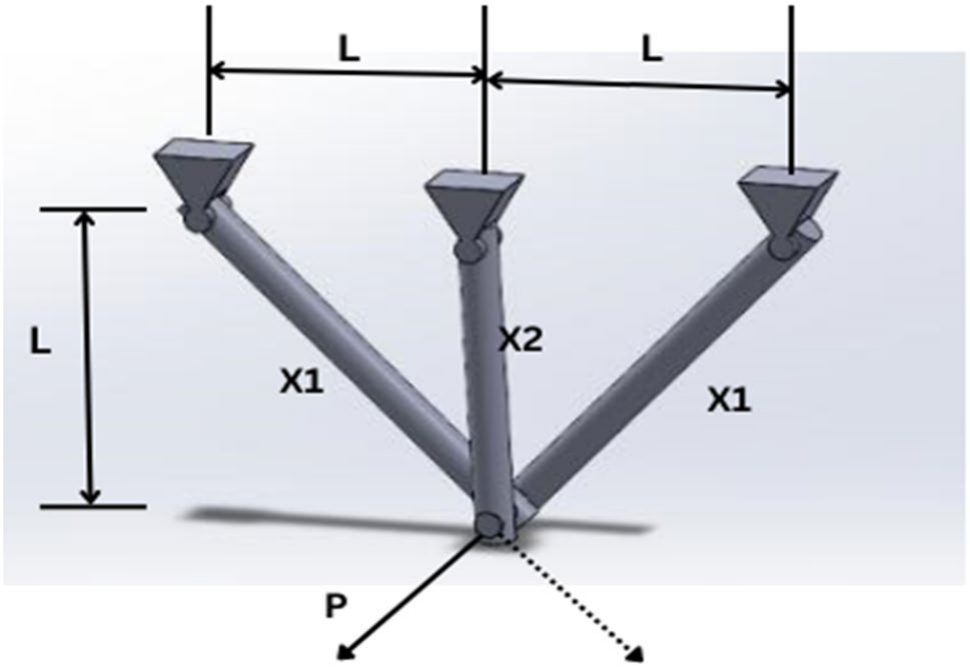
Three-bar truss design.

The main purpose of truss design is reduction in weight of the bar structures. This caused the position of two bars to be three bars as represented in Fig. 17, since it is required to minimize the weight of the bars in this position. The issue is three-restrictive, and the functions of it are the constraints of deflection of any given bar, buckling, stress and design parameters $\left( {x_{1} ,x_{2} } \right)$^84^.

The mathematical formulation of this problem is the following: 

Minimize

37 $$ f\left( x \right) = \left( {2\sqrt 2 x_{1} + x_{2} } \right) \times L $$

$$ {\text{Subject }}\,{\mathrm{to}}\,\,q_{1} = \frac{{\sqrt 2 x_{1} + x_{2} }}{{\sqrt 2 x_{1}^{2} + 2x_{1} x_{2} }} \times P - \sigma \le 0 $$

$$ q_{2} = \frac{{x_{2} }}{{\sqrt 2 x_{1}^{2} + 2x_{1} x_{2} }} \times P - \sigma \le 0 $$

$$ q_{3} = \frac{1}{{\sqrt 2 x_{1} + x_{2} }} \times P - \sigma \le 0 $$

where $0 \le x_{1} , \,x_{2} \le 1.$ the constants are as follows: $L = 100\, {\mathrm{cm}}, \,\,P = 2 \,{\mathrm{KN/cm}}^{2}$ and ${ }\sigma = 2 KN/cm^{2}$.

#### DMARS_WGO vs AIRE_WGO, GOA, and WO

**Fig. 18:**
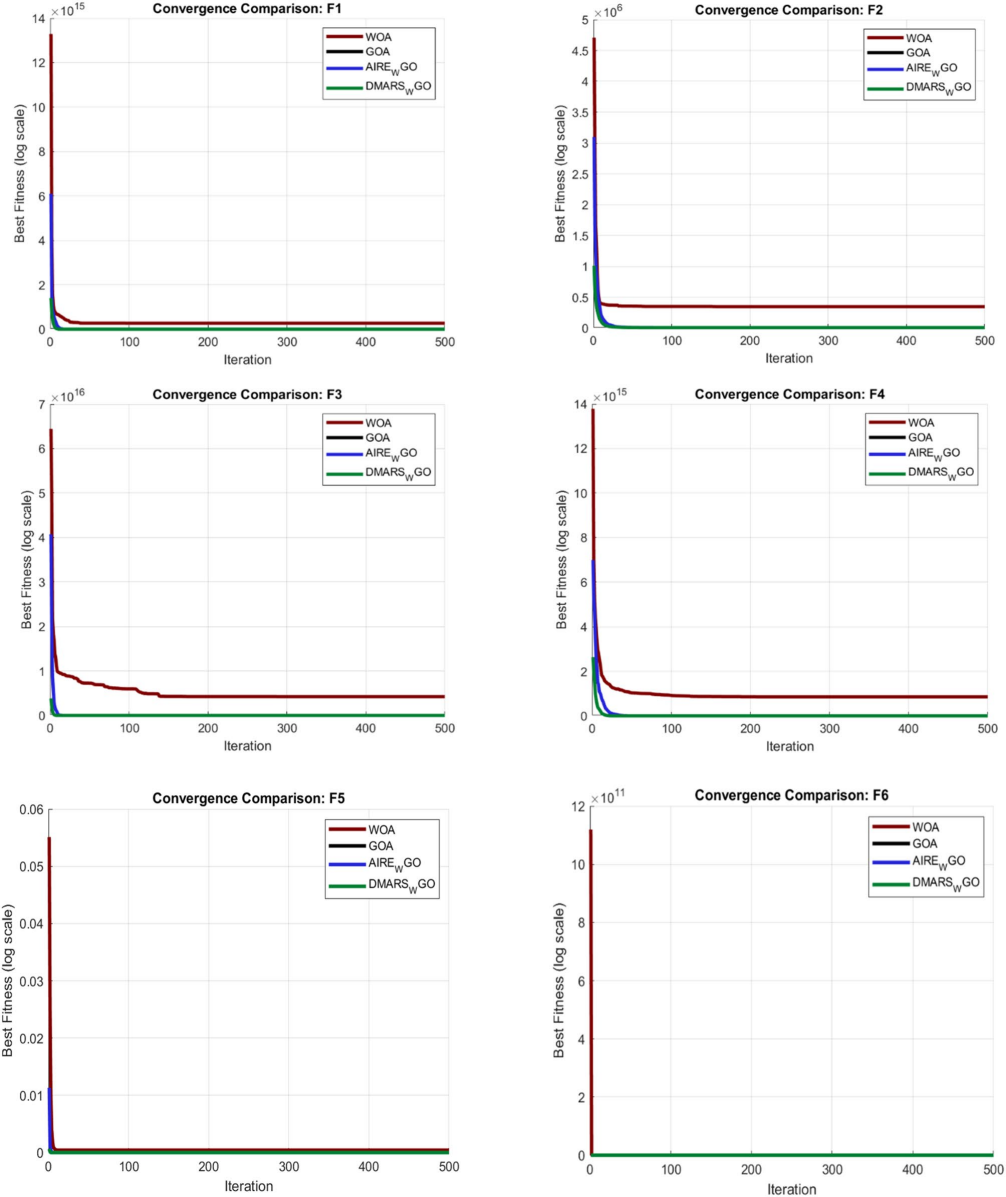
Samples of convergence plots of DMARS_WGO, AIRE_WGO, GOA and WO on 6 engineering problems.

**Fig. 19:**
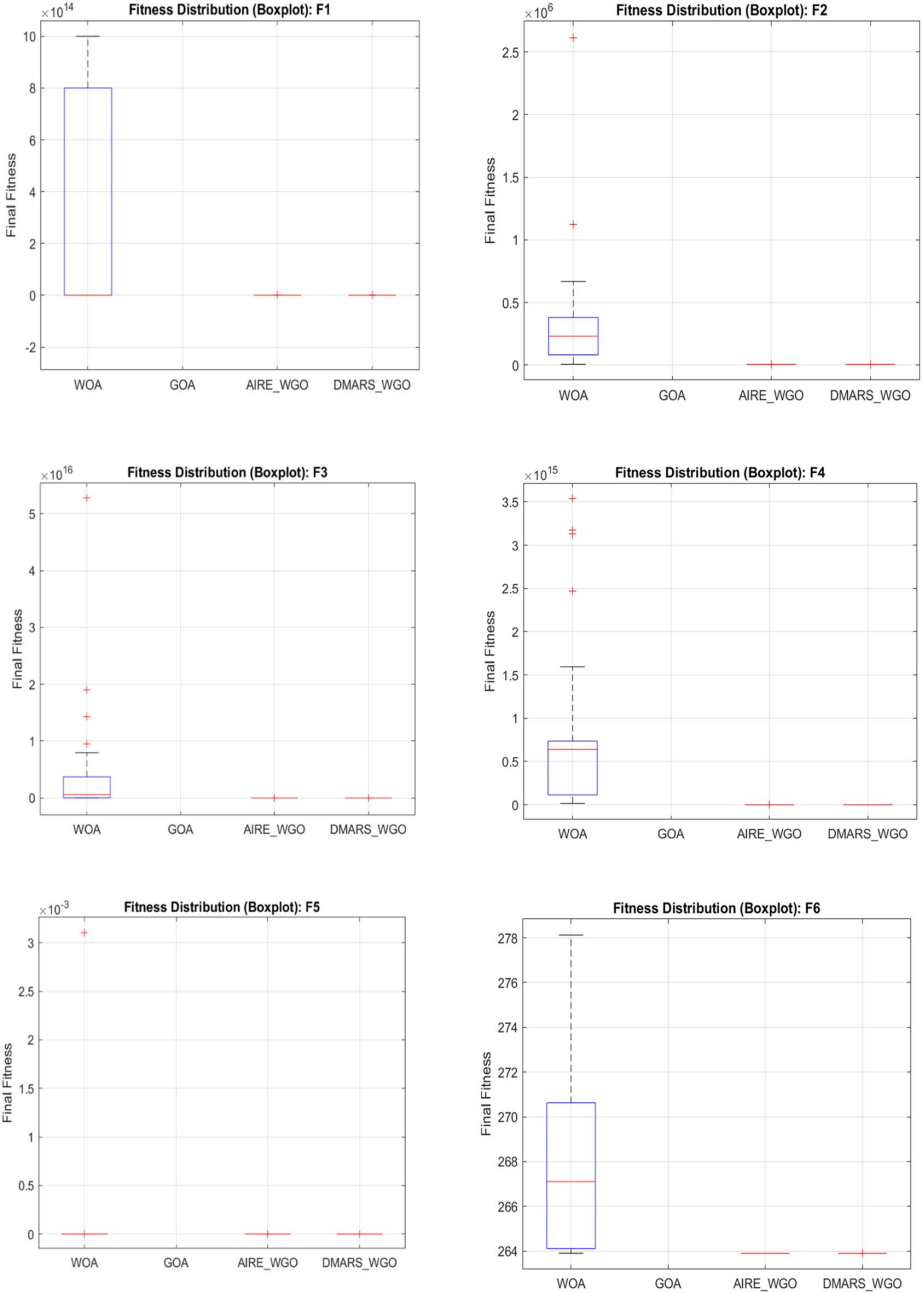
Samples of boxplot of DMARS_WGO, AIRE_WGO, GOA and WO on 6 engineering problems.

The proposed algorithms are tested on the 6 engineering problems. This comparison with AIRE_WGO, GOA, and WO highlights the consistency of DMARS_WGO across different problem complexities and benchmark generations. Table 12 summarizes the obtained best, mean, and standard deviation values, as well as the overall ranking for each problem. DMARS_WGO achieves the best mean values for all problems and it ranks first at all problems. The Wilcoxon signed-rank test (*p* < 0.05) validates that the improvements achieved by DMARS_WGO are statistically significant when compared to the other algorithms as shown as in Table 13. As illustrated in Fig. 18, the convergence plots clearly show that DMARS_WGO attains the optimal regions faster and maintains a smooth and stable convergence behavior throughout the iterative process. Figure 19 shows Boxplot comparisons of DMARS_WGO and the compared algorithms on the 6 problems and illustrates the higher stability and lower result dispersion of DMARS_WGO.

**Table 12:** Comparison of results of DMARS_WGO, AIRE_WGO, GOA and WO on 6 engineering problems.

F	Algorithm	WO	GOA	AIRE_WGO	DMARS_WGO
F1	Best	0.012665276	65,535	0.012665901	0.012666602
	Mean	2.70862E + 14	65,535	0.012730595	**0.012674626**
	Std	4.24813E + 14		0.000101254	1.28021E-05
	Rank	4	3	2	**1**
F2	Best	7022.363661	65,535	5885.339673	5885.334779
	Mean	345,933.5721	65,535	5973.923881	**5887.115886**
	Std	494,382.2804		121.842165	2.60946658
	Rank	4	3	2	**1**
F3	Best	2.376385998	65,535	1.724852309	1.724852309
	Mean	4.25165E + 15	65,535	1.724853048	**1.724852315**
	Std	1.02328E + 16		2.03235E − 06	7.74329E-09
	Rank	4	3	2	**1**
F4	Best	1.61405E + 13	65,535	733.5659603	733.7867874
	Mean	8.50764E + 14	65,535	754.8717348	**747.3330697**
	Std	9.7491E + 14		12.96997934	8.33808111
	Rank	4	3	2	**1**
F5	Best	0	65,535	3.16478E − 17	4.9224E-20
	Mean	0.000413972	65,535	2.46553E − 14	**3.03656E-16**
	Std	0.00107347		7.45994E − 14	5.34013E-16
	Rank	3	4	2	**1**
F6	Best	263.9026958	65,535	263.895852	263.8958511
	Mean	267.9462099	65,535	263.8960964	**263.8959237**
	Std	4.125805876		0.000190045	6.78831E-05
	Rank	3	4	2	**1**
Average		3.666666667	3.333333333	2	**1**
Total Rank		4	3	2	**1**

**Table 13:** The Wilcoxon rank-sum test (*p*-value) for DMARS_WGO, AIRE_WGO, GOA and WO for 6 engineering problems.

DMARS vs	WO	GOA	AIRE_WGO
F1	8.18313E − 07***	1.21178E − 12***	5.26501E − 05***
F2	3.01986E − 11***	1.21178E − 12***	1.4918E − 06***
F3	3.01986E − 11***	1.21178E − 12***	3.5708E − 06***
F4	2.95426E − 11***	1.21178E − 12***	0.003182959**
F5	1.56656E − 06***	1.21178E − 12***	1.59641E − 07***
F6	3.01986E − 11***	1.21178E − 12***	0.000104066***

*denotes statistical significance at *p* < 0.05, while multiple asterisks indicate stronger significance levels.

#### DMARS_WGO vs other algorithms

**Fig. 20:**
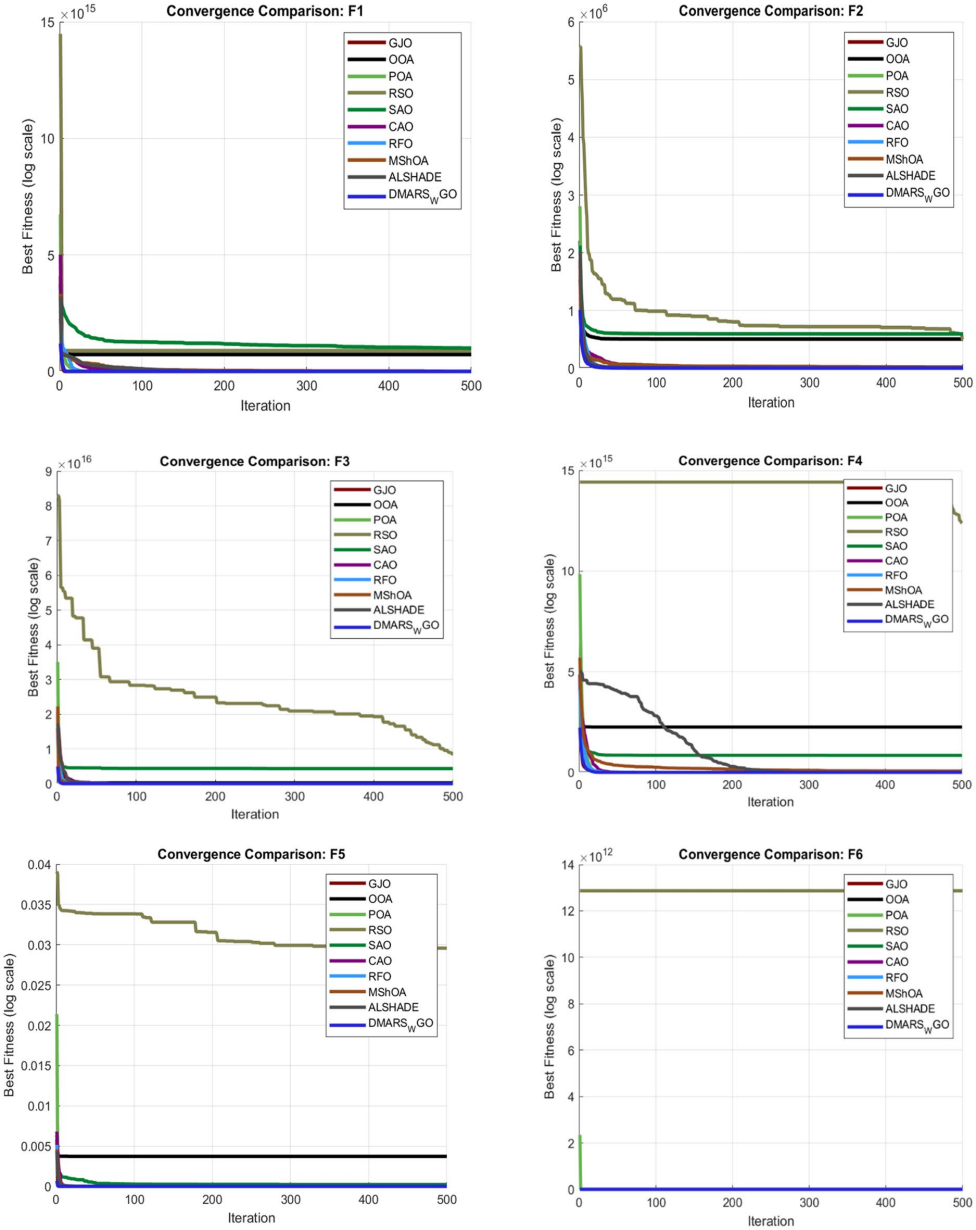
Samples of convergence plots of DMARS_WGO vs other algorithms on 6 engineering problems.

**Fig. 21:**
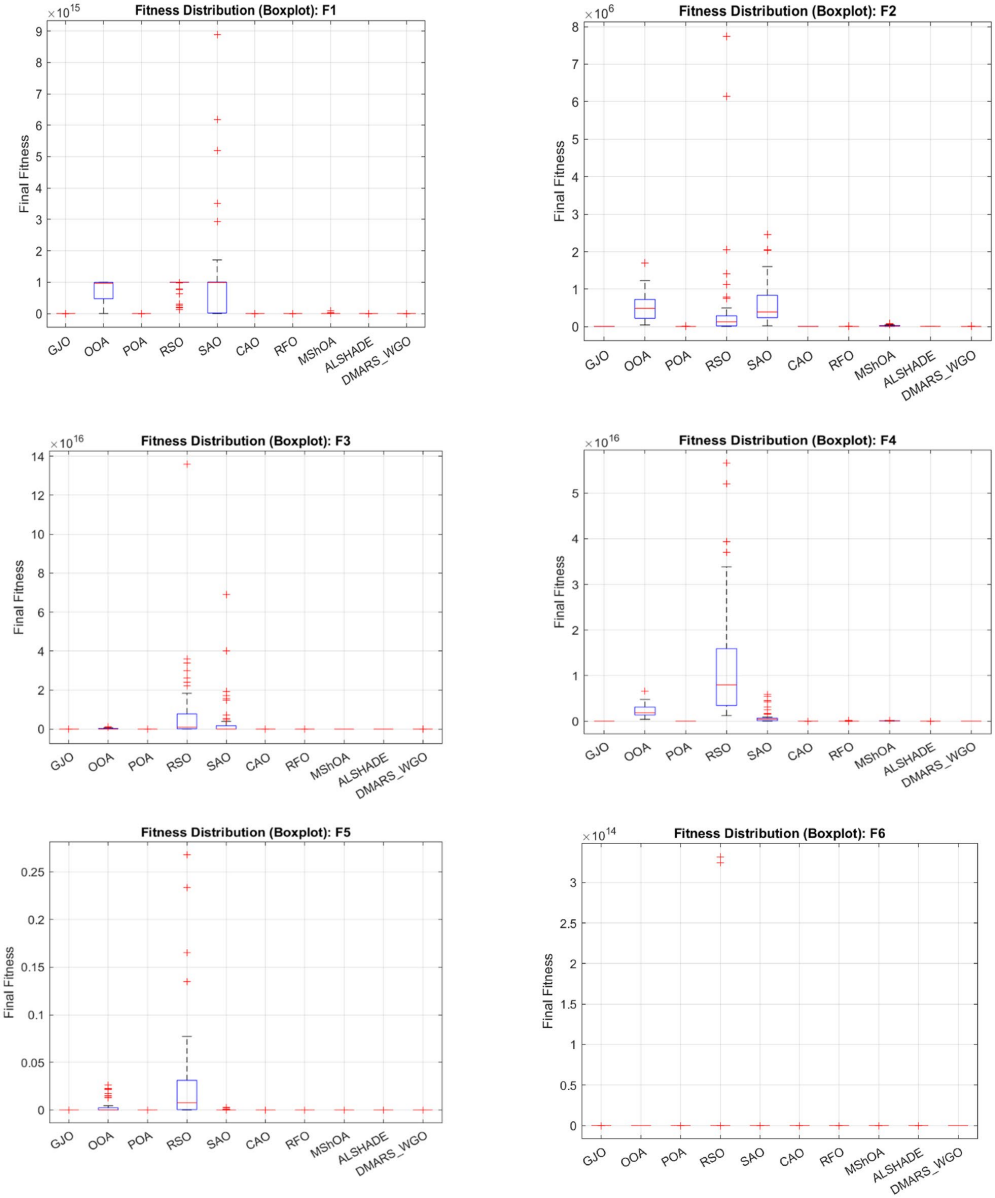
Samples of boxplot of DMARS_WGO vs other algorithms on 6 engineering problems.

The proposed DMARS_WGO is also compared with various optimization algorithms, namely GJO, OOA, POA, RSO, SAO, CAO, RFO, MShOA, and ALSHADE as done in CEC 2017 and CEC 2022. The comparative performance of DMARS-WGO and other algorithms is summarized in Table 14, which reports the best, mean, standard deviation, and rank values for each problem. DMARS_WGO achieves the best mean in all functions except (F5, F6). RFO records the best mean values on F5. POA records the best mean values on F6. The Wilcoxon signed-rank test (*p* < 0.05), shown in Table 15, indicates that DMARS-WGO achieves statistically significant improvements over the compared algorithms, validating the reliability of its results. As illustrated in Fig. 20, the convergence plots show that DMARS-WGO converges faster toward the optimal feasible regions and maintains smooth, stable progress throughout the optimization process. Similarly, the boxplot comparisons in Fig. 21 highlight its lower result dispersion and higher consistency, confirming that DMARS-WGO delivers superior convergence stability and robustness across all engineering problems.

**Table 14:** Comparison of results of DMARS_WGO vs other algorithms on 6 engineering problems.

F	Algo	GJO	OOA	POA	RSO	SAO	CAO	RFO	MShOA	ALSHADE	DMARS_WGO
F1	Best	0.012718907	0.015045898	0.012665237	1.32392E + 14	0.020693671	0.012748662	0.012665233	0.012803932	0.01272852	0.012666844
	Mean	0.012786465	7.26174E + 14	0.012754174	8.91543E + 14	9.99438E + 14	0.016918683	0.012680718	3.36015E + 12	0.013266009	**0.01267166**
	Std	0.000108758	3.64052E + 14	0.000171046	2.5526E + 14	1.67024E + 15	0.00326763	3.27845E − 05	1.29476E + 13	0.000524602	3.36525E-06
	Rank	4	8	3	9	10	6	2	7	5	**1**
F2	Best	5904.975653	44,665.4178	5885.332779	8338.739339	23,272.95598	5890.080479	5885.332774	7307.803614	7852.100464	5885.358461
	Mean	6989.969493	508,012.1033	6194.516388	496,956.1637	596,888.7767	6505.305519	6049.019617	22,705.30761	8453.909336	**5888.621289**
	Std	744.558434	344,509.7961	454.576756	1,380,404.013	551,341.9116	473.0006517	276.4168464	13,948.23178	262.8802131	6.476278224
	Rank	5	9	3	8	10	4	2	7	6	**1**
F3	Best	1.727333867	3.214399123	1.72486546	3.625678202	2.164012951	1.729260096	1.724852309	2.087710358	1.760622941	1.724852309
	Mean	1.73858984	2.10224E + 14	1.726978859	8.53615E + 15	4.34928E + 15	2.269290358	1.726481754	2.847337379	1.850605693	**1.724852316**
	Std	0.01020326	3.35473E + 14	0.002949951	2.0553E + 16	1.17029E + 16	0.935512462	0.011454075	0.392952086	0.035570261	7.55812E-09
	Rank	4	8	3	10	9	6	2	7	5	**1**
F4	Best	764.324233	3.91112E + 14	733.9301876	1.21496E + 15	1.23205E + 12	759.5345848	765.011124	4.41033E + 11	799.413349	733.6650866
	Mean	797.2639668	2.25146E + 15	763.7360021	1.23684E + 16	8.42963E + 14	814.6088886	2.97528E + 12	6.48476E + 13	978.4787229	**749.9049064**
	Std	20.70491075	1.36413E + 15	18.28121905	1.2894E + 16	1.38147E + 15	37.33806534	2.05807E + 13	5.47584E + 13	158.0844599	8.481892838
	Rank	3	9	2	10	8	4	6	7	5	**1**
F5	Best	1.65205E − 14	0	0	1.05874E − 06	1.36899E − 14	3.84953E − 19	0	8.83933E − 12	1.6776E − 14	1.40178E-20
	Mean	2.08914E − 11	0.003728322	8.33436E − 27	0.029578328	0.000225497	3.21186E − 13	**2.30054E − 32**	9.23724E − 08	5.5613E − 11	3.09477E-16
	Std	4.04583E − 11	0.007174624	3.99277E − 26	0.055690117	0.000586695	6.57001E − 13	4.36923E − 32	1.83454E − 07	1.13704E − 10	7.29544E-16
	Rank	5	9	2	10	8	4	**1**	7	6	3
F6	Best	263.8967528	263.9311913	263.8958434	264.5090648	264.1385545	263.8963016	263.8958434	263.8964631	263.9147705	263.8958509
	Mean	263.9235892	266.003171	**263.8958434**	1.28677E + 13	273.573208	264.008065	263.8958434	264.0038698	264.4181495	263.895931
	Std	0.026711609	2.230977702	5.90734E − 14	6.43306E + 13	6.697565128	0.289419171	4.72887E − 12	0.12766352	2.633863509	6.0929E-05
	Rank	4	8	**1**	10	9	6	2	5	7	3
Average		4.166666667	8.5	2.333333333	9.5	9	5	2.5	6.666666667	5.666666667	**1.666666667**
Total Rank		4	8	2	10	9	5	3	7	6	**1**

**Table 15:** The Wilcoxon rank-sum test (p-value) for DMARS_WGO Vs all other algorithms for 6 problems.

DMARS vs	GJO	OOA	POA	RSO	SAO
F1	3.30368E − 18***	3.30368E − 18***	0.006069369**	3.30368E − 18***	3.30368E − 18***
F2	4.18122E − 18***	3.30368E − 18***	1.24054E − 05***	3.30368E − 18***	3.30368E − 18***
F3	3.30368E − 18***	3.30368E − 18***	3.30368E − 18***	3.30368E − 18***	3.30368E − 18***
F4	3.30368E − 18***	3.30368E − 18***	9.39833E − 06***	3.30368E − 18***	3.30368E − 18***
F5	3.30368E − 18***	2.08735E − 11***	1.09082E − 18***	3.30368E − 18***	3.30368E − 18***
F6	3.30368E − 18***	3.30368E − 18***	1.98815E − 20***	3.30368E − 18***	3.30368E − 18***

DMARS vs	CAO	RFO	MShOA	ALSHADE
F1	3.30368E − 18***	0.103879923 ns	3.30368E − 18***	3.30368E − 18***
F2	4.9869E − 18***	0.084218233 ns	3.30368E − 18***	3.30368E − 18***
F3	3.30368E − 18***	0.003302321**	3.30368E − 18***	3.30368E − 18***
F4	3.30368E − 18***	3.30368E − 18***	3.30368E − 18***	3.30368E − 18***
F5	6.5879E − 14***	2.99132E − 18***	3.30368E − 18***	3.30368E − 18***
F6	3.30368E − 18***	3.29653E − 18***	3.30368E − 18***	3.30368E − 18***

*denotes statistical significance at *p* < 0.05, while multiple asterisks indicate stronger significance levels.

## Diversity regulation discussion

DMARS_WGO incorporates an explicit diversity-aware control mechanism. During early search stages, GOA-based exploratory dynamics promote spatial dispersion among agents, maintaining high population diversity. As promising regions are identified, the WO-based exploitation strategy gradually reduces diversity to refine convergence. In addition, the adaptive mutation operator is activated when stagnation is detected, reintroducing controlled perturbations to prevent premature collapse of the population.

Unlike static metaheuristics with predetermined exploration schedules, the dual-mode reinforcement mechanism continuously monitors diversity and improvement rate, dynamically adjusting behavioral dominance. This results in a smoother diversity decay pattern and improved robustness on multimodal and composition functions, where premature convergence is common in traditional algorithms.

## Mechanistic explanation of performance improvement

In addition to the reported empirical performance improvement, the enhanced optimization behavior of DMARS_WGO can be explained by the fact that the structural role of the dual-mode reinforcement mechanism in the DMARS regulate search dynamics. In contrast to the traditional metaheuristics, which depend on fixed operator schedules or fixed parameters adjustment, DMARS_WGO constantly measures population-based indicators, such as diversity, improvement rate, and stagnation. These cues give contextual feedback which allows adaptive control of exploration–exploitation dominion during the optimization process. Earlier in the search, greater diversity and exploration-based behavior are encouraged permitting an extensive sampling of the search space and minimizing the likelihood of a premature convergence. When stagnation or a decreasing improvement in the process is detected by the reinforcement mechanism, the behavioral dominance is dynamically altered during the intermediate iterations in order to reintroduce diversification when needed. At the last stage, the blending coefficient $\lambda_{t}$ approaches the exploitation-dominant movements and the search is biased to achieve the refined convergence to promising regions with no sudden oscillatory switching. Tabular Q-learning and DQN are further enhanced to allow a cooperative interaction that improves stability. Whereas Q-learning offers a rapid appeasement on a brief-term scale, guided by the newfound response, the DQN element allows approximations of values on a generalized level, across environmental setup arrangements. This unending mixing of these two learners softens the sudden change of policy and lessens the behavior switching volatility. Together, these processes produce a regulated and context sensitive process of exploration to exploitation, which accounts for the more fluent convergence process and performance in unimodal, multimodal, hybrid, and constrained benchmark problems.

## Parameter sensitivity analysis

In order to assess the strength of DMARS_WGO and measure the effect of the primary control parameters, a parameter sensitivity analysis was performed. As DMARS WGO incorporates several adaptive mechanisms, the parameters were grouped into categories of three: Parameters of reinforcement learning: α (learning rate), ${\upgamma }$ (discount factor), ε0 (initial exploration rate). Parameters of switching and mixing: $\lambda_{t}$ (blending coefficient control), $w_{1}$, ${ }w_{2}$, ${ }w_{3}$ (weighting coefficients). Parameters of diversity and mutation: $p_{m0}$ (initial mutation probability), η0 (mutation decay rate), and the bounds βmin, βmax, Φmin, Φmax. One-factor-at-a-time (OAT) strategy was selected: one parameter was changed at the time leaving all the other parameters at their default value. This algorithm was run 51 independent runs in each setting on a representative sample of benchmark functions (unimodal, multimodal, hybrid, and composition). The mean best fitness, standard deviation, and the Friedman ranking were used to evaluate the performance. The sensitivity results indicate that DMARS_WGO is stable in a reasonable parameter range without sharp degradation when perturbed with moderate changes. Specifically, the algorithm is less sensitive with regard to switching/blending limits, whereas the reward weight and exploration rate are the key factors that influence the exploration at an early stage. These results really prove that proposed framework is strong and can be reproduced with experimental parameters variations. The detailed parameter sensitivity ranges are summarized in Table 16.

**Table 16:** Parameter sensitivity settings.

Parameter	Range
$${\upgamma }$$	[0, 1]
α	[0, 1]
ε0	0.2, 0.4, 0.6, 0.8
$p_{m0}$	0.05, 0.1, 0.2, 0.3
$w_{1}$,${ }w_{2}$,${ }w_{3}$	± 20% each
βmin/βmax	± 20% bounds
Φmin/Φmax	± 20% bounds

## Conclusion and future work

Hybrid metaheuristic optimization algorithms have become as powerful and indispensable methods for solving complex, nonlinear, and high-dimensional engineering problems. Nevertheless, a number of these algorithms still have certain underlying constraints, such as stalling, early convergence, and inability to adapt to dynamic search topography. In this article, two smart reinforcement-based hybrid structures, are Adaptive Intelligent Reinforced Walrus-Gazelle Optimizer (AIRE_WGO) and the Dual-Mode Adaptive Reinforced Switching Walrus-Gazelle Optimizer (DMARS_WGO), were introduced in order to address such challenges and to present a new self-learning and adaptive optimization paradigm.

The initial algorithm, AIREWGO, is a variant of the classical Walrus Gazelle model, augmented with Q-learning-based behavioral control, adaptive parameter control, and diversity-sensitive mutation techniques, and can therefore perform an intelligent search between broad search and fine-grained exploitation. Based on this premise, the improved DMARS_WGO incorporates a two-mode reinforcement architecture that combines tabular Q-learning and deep Q-network (DQN) logic. This architecture enables the optimizer to switch its learning dominance dynamically by soft-switching and a dominance-adjustment mechanism, based on population diversity, rate of fitness improvement, and feedback of stagnation. Additionally, the cross-agent knowledge-sharing process will enable a process of cooperative transfer between the Q-learning and the DQN agents and significantly enhance the depth, stability, and flexibility of learning. The results of the two algorithms have been thoroughly tested on both CEC2017 and CEC2022 benchmark suites and six real-life engineering tasks. It was found that the proposed DMARS_WGO was found to be significantly more accurate, robust and scaled well than nine state-of-the-art optimizers, including GJO, OOA, POA, RSO, SAO, CAO, RFO, MShOA, and ALSHADE. Experimental validation on the CEC2017 benchmark suite demonstrated that DMARS_WGO achieved first rank in 26 out of 29 functions with an overall Friedman mean rank of 1. On CEC2022, it ranked first in 8 out of 12 functions, while on six engineering design problems it secured the best performance in 4 cases. Statistical tests on the Wilcoxon Signed-Rank test (*p* < 0.05) and Friedman mean-rank test proved that DMARS_WGO is the first in the overall performance and thus is able to balance the deep exploration and global exploration with the precision never before. Compared with the single-mode AIRE_WGO, the dual-agent DMARS_WGO demonstrated an excellent capability to self-adapt its learning policy and be stable even on extremely irregular landscapes. The combination of an adaptive control of dominance and reinforcement feedback with knowledge exchange among the agents allowed the algorithm to automatically emerge out of stagnation and seek the global optimum continuously. All these features allow DMARS_WGO to be a potent, smart, and self-developing framework to optimize complex engineering and real-life scenarios.

Though DMARSWGO has shown good results in benchmark and classical engineering problems, the method is not practical in real-world applications of engineering problems unless calibration to specific domain and incorporation with real engineering simulation environments. The sensitivity analysis of the parameters shows that the framework does not need the fine-tuning. In addition, the computational cost added by the reinforcement module is moderate as compared to that of objective function assessment. Thus, the deployment of the described framework into large-scale industrial applications on the short term was not the focus of this study; although the framework would be practically applicable in the context of realistic computational conditions.

In the future, based on the existing results, there are a number of research directions, which are technical in nature. To start with, the expansion of DMARS_WGO to multi-objective optimization would enable the assessment of the dual-mode reinforcement system in a trade-off-based search setting. Second, further testing its resilience in non-stationary environments would be to adjust the framework to dynamic and noisy optimization problems. Also, the incorporation of surrogate-assisted learning can help to cut down the computational load during costly objective appraisals. Lastly, parallel and distributed processing would be an improvement in the case of large-scale optimization processes. These extensions are logical extensions of the existing reinforcement-based architecture and offer viable directions to future research.

## Data Availability

No datasets were generated or analysed during the current study.
